# A New Megaraptoran Dinosaur (Dinosauria, Theropoda, Megaraptoridae) from the Late Cretaceous of Patagonia

**DOI:** 10.1371/journal.pone.0157973

**Published:** 2016-07-20

**Authors:** Rodolfo A. Coria, Philip J. Currie

**Affiliations:** 1 Consejo Nacional de Investigaciones Científicas y Técnicas, Gral. Roca, Argentina; 2 Universidad Nacional de Río Negro, Gral. Roca, Argentina; 3 Secretaria de Estado de Cultura de Neuquén, Museo Carmen Funes, Plaza Huincul, Neuquén, Argentina; 4 University of Alberta, Edmonton, Alberta, Canada; College of the Holy Cross, UNITED STATES

## Abstract

A skeleton discovered in the Upper Cretaceous Sierra Barrosa Formation (Turonian-Coniacian) of Neuquén Province, Argentina represents a new species of theropod dinosaur related to the long snouted, highly pneumatized Megaraptoridae. The holotype specimen of *Murusraptor barrosaensis* n.gen et n.sp. (MCF-PVPH-411) includes much of the skull, axial skeleton, pelvis and tibia. *Murusraptor* is unique in having several diagnostic features that include anterodorsal process of lacrimal longer than height of preorbital process, and a thick, shelf-like thickening on the lateral surface of surangular ventral to the groove between the anterior surangular foramen and the insert for the uppermost intramandibular process of the dentary. Other characteristic features of *Murusraptor barrosaensis* n.gen. et n. sp.include a large mandibular fenestra, distal ends of caudal neural spines laterally thickened into lateral knob-like processes, short ischia distally flattened and slightly expanded dorsoventrally. *Murusraptor* belongs to a Patagonian radiation of megaraptorids together with *Aerosteon*, *Megaraptor* and *Orkoraptor*. In spite being immature, it is a larger but more gracile animal than existing specimens of *Megaraptor*, and is comparable in size with *Aerosteon* and *Orkoraptor*. The controversial phylogeny of the Megaraptoridae as members of the Allosauroidea or a clade of Coelurosauria is considered analyzing two alternative data sets.

## Introduction

During the last decade, many new types of theropod dinosaurs have been described from the Upper Cretaceous sediments of South America. These include abelisauroids [1–12], carcharodontosaurids [13–15], dromaeosaurids [16–18] and an assortment of species whose affinities are less certain [19–20].

In 2001, the Argentinean-Canadian Dinosaur Project team collected fossils on Sierra Barrosa, 30 km northeast of Plaza Huincul, Neuquén Province, Argentina. The rocks of this locality represent the last stratigraphic sequence of Upper Cretaceous beds of the Neuquén Group, and yield a unique association of terrestrial vertebrates including skeletons of turtles, crocodiles, saurischian dinosaurs, mammals and footprints of birds [21–24]. During the course of that expedition, a partial skeleton of a meat-eating dinosaur was recovered. The holotype specimen (MCF-PVPH-411) of *Murusraptor barrosaensis* n. gen. et sp. was approximately 6.5 meters long when it was alive, and had a proportionally long and low skull, small teeth, and highly pneumatized bones.

In a recent years, the Megaraptora [25],a clade of medium-sized, large-clawed and highly pneumatized theropods, was built to include the South American species *Aerosteon riocoloradensis* [26], *Megaraptor namunhaiquii* [27] and *Orkoraptor burkei* [28], together with the Australian *Australovenator wintonensis* [29], and the Japanese *Fukuiraptor kitadaniensis* [30]. Subsequently, the record of megaraptorans has been steadly nurtured by reports of the existence of their remains from Central Patagonia, Brazil and Australia [31–34] and our anatomical knowledge of them has been substantially expanded by the descriptions of more informative specimens [35–36].

The enigmatic nature of this group has been a matter of discussion since the description of the first megaraptoran, *Megaraptor namunhaiquii* [27]. The interpretation of this taxon, based on its taxonomic relationships has migrated from one as an intriguing coelurosaur [27], to one as a basal Tetanurae with uncertain affinities [8], and finally to be considered the type genus of the Megaraptora [25], which was defined as the most inclusive clade comprising *Megaraptor namunhaiquii* but not *Chilantaisaurus tashiukouensis*, *Neovenator salerii*, *Carcharodontosaurus saharicus* or *Allosaurus fragilis* [25]. This is a stem-based taxon which, despite its broadly accepted and consensual validity, still presents some debate regarding their phylogenetic relationships within Theropoda. More recently [37] the clade Megaraptoridae was defined as a stem-based clade including all theropods closer to *Megaraptor namunhuaiquii* than to *Fukuiraptor kitadaniensis*, *Chilantaisaurus tashiukouensis*, *Neovenator salerii*, *Carcharodontosaurus saharicus*, *Allosaurus fragilis*, *Baryonyx walkeri*, *Tyrannosaurus rex*, and *Passer domesticus*, and involving within the clade, all South American forms plus *Australovenator*.

Currently, the position of the megaraptorids among theropods is the subject of two alternative hypothesis: 1) as derived allosauroid neovenatorids [25,38–39], and 2) as tyrannosauroid coelurosaurs [35,37]. These different approaches of the phylogenetic relationships of this clade, although they both agree in the internal taxonomic composition, radically differ in regard the ancestry of the group. These contentious phylogenetic hypotheses on both sides of the allosauroid-coelurosaur dichotomy are far from resolution, as it has been recognized in the most recent contributions [34,40].

*Murusraptor barrosaensis* is one of the most informative megaraptorids known, having preserved the posterior elements of the skull and several postcranial elements. Although the goal of this contribution is not the reexamination of the phylogenetic relationships of the megaraptorids, the specimen MCF-PVPH-411 of *Murusraptor* yields an important amount of new anatomical information that can be useful for future and deeper phylogenetical treatment of the clade.

### Institutional Abbreviations

MCF-PVPH, Museo Carmen Funes, Paleontologia de Vertebrados, Plaza Huincul, Neuquén, Argentina; MCNA, Museo de Ciencias Naturales y Antropologicas J.C. Moyano, Mendoza, Argentina; MUCPv-CH, Museo de la Universidad Nacional del Comahue, El Chocón collection, Neuquén, Argentina.

## Materials and Methods

### Paleontological Ethics Statements

All necessary permits were obtained for the described study, which complied with all relevant regulations. The holotype specimen was collected under permits obtained by RAC from the Dirección General de Patrimonio Cultural [Subsecretaría de Cultura, de la Provincia del Neuquén] for work conducted in the Sierra Barrosa, Provincia del Neuquén during 2001/2002.

The holotype specimen (MCF-PVPH-411) of *Murusraptor barrosaensis* gen et sp nov. described in this paper is housed at public and permanent repository of the Museo Carmen Funes of the city of Plaza Huincul, Neuquén Province, Argentina.

### Nomenclatural Acts

The electronic edition of this article conforms to the requirements of the amended International Code of Zoological Nomenclature, and hence the new names contained herein are available under that Code from the electronic edition of this article. This published work and the nomenclatural acts it contains have been registered in ZooBank, the online registration system for the ICZN. The ZooBank LSIDs (Life Science Identifiers) can be resolved and the associated information viewed through any standard web browser by appending the LSID to the prefix "http://zoobank.org/". The LSID for this publication is: urn:lsid:zoobank.org:act: 05EA2F7C-68AF-4E07-9171-3432AC3B80C2. The electronic edition of this work was published in a journal with an ISSN, and has been archived and is available from the following digital repositories: PubMed Central, LOCKSS.

## Results and Discussion

### Systematic Paleontology

Dinosauria Owen, 1842

Theropoda Marsh, 1881

Tetanurae Gauthier, 1986

Megaraptora Benson, Carrano, Brusatte, 2010

Megaraptoridae Novas, Agnolin, Ezcurra, Porfiri, Canale 2013

*Murusraptor barrosaensis* new genus, new species

#### Etymology

“*Murus*” is a Latin term for “wall”, referring to the discovery of the specimen in the wall of a canyon; “*barrosaensis*” alludes to Sierra Barrosa, the locality where it was collected.

#### Holotype

**Fig 1:**
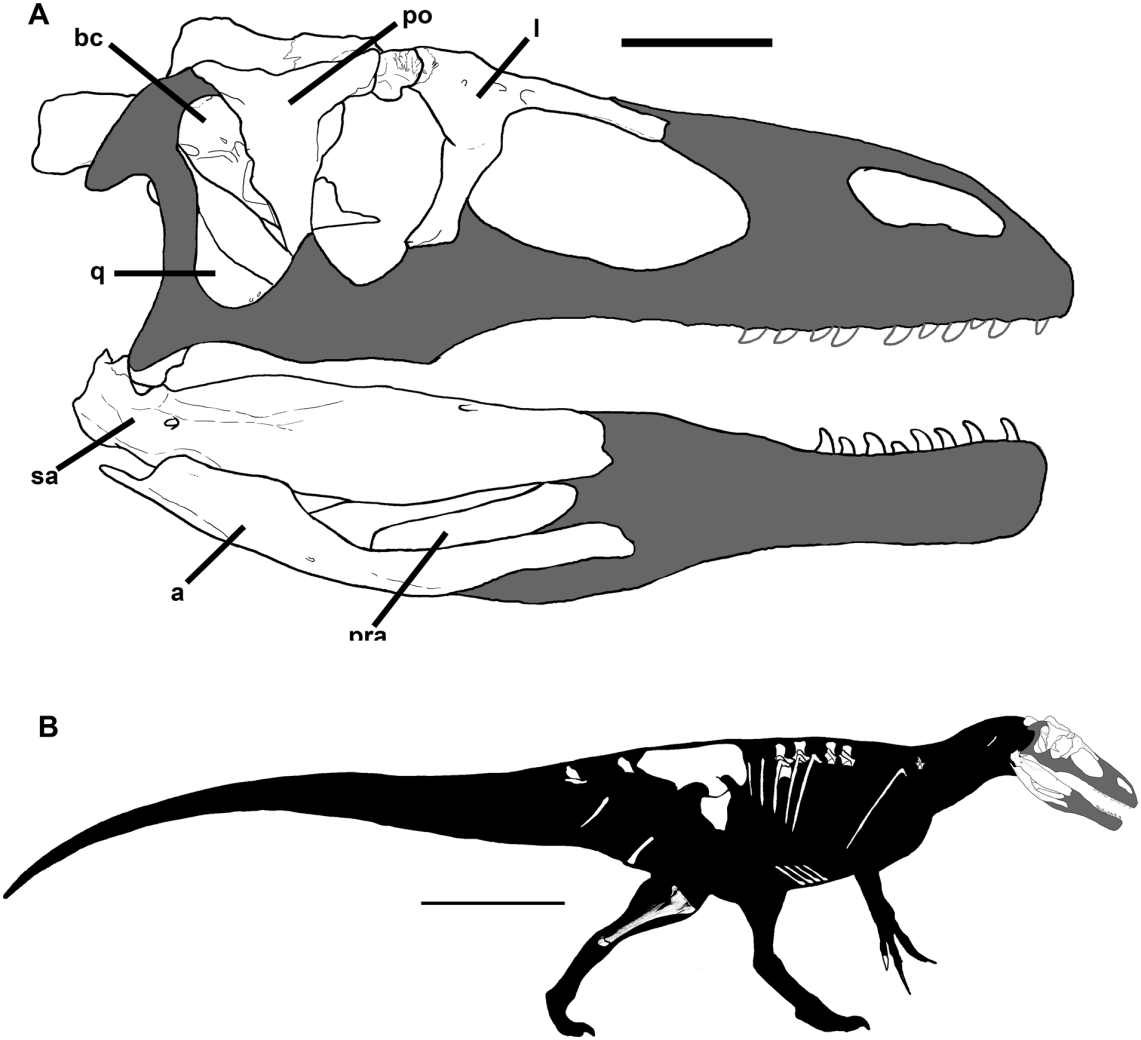
A) Skull reconstruction of *Murusraptor barrosaensis*, MCF-PVPH-411. B) Body reconstruction of *Murusraptor barros*aensis, MCF-PVPH-411. Both illustrations show recovered elements in white. Scale bars: A = 10 cm, B = 1 m.

Partial skeleton (Museo Carmen Funes MCF-PVPH-411) includes a complete braincase, lacrimal, prefrontal, postorbital, quadrate, pterygoid, ectopterygoid, teeth, twelve vertebrae, eleven thoracic ribs, one haemal arch, several gastralia, a manual ungual, complete left ilium, part of right ilium, proximal ends of the pubes, distal ends of the ischia, the right tibia, and a calcaneum (Fig 1).

#### Locality and horizon

**Fig 2:**
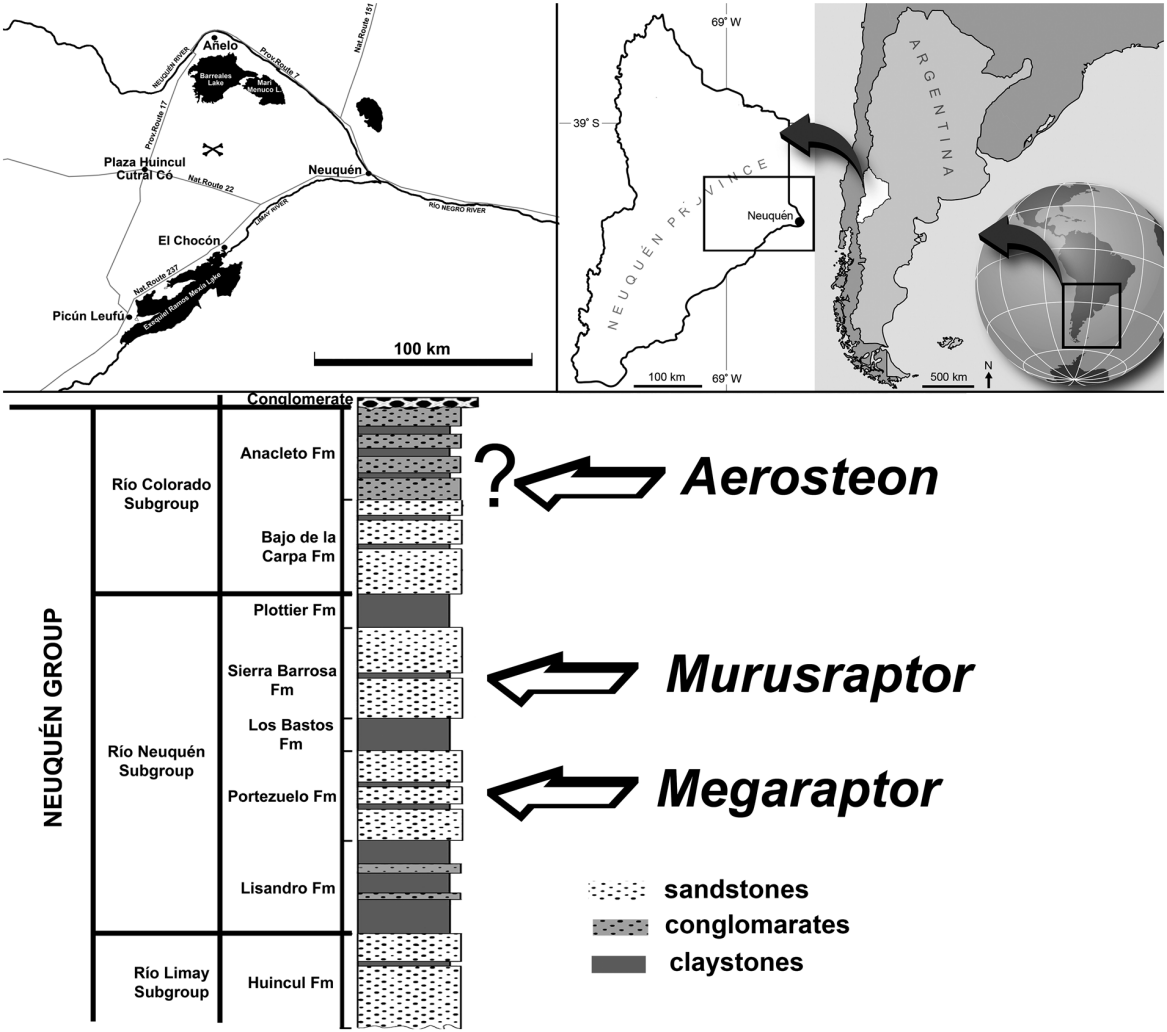
Location and geological context of the holotypeof *Murusraptor barrosaensis*, MCF-PVPH-411. A) Star marks collecting locality of holotype. B) Stratigraphic table and geologic section indicating the provenance of the megaraptorins recorded in the Neuquén group (modified from [41]).

Sierra Barrosa, northeast of Plaza Huincul, Neuquén Province, Argentina. S 38° 50.7001’, W 68° 50.3256’ (WGS84). Sierra Barrosa Formation (Coniacian), Río Neuquén Subgroup, Neuquén Group, Upper Cretaceous [41] (Fig 2).

#### Diagnosis

*Murusraptor barrosaensis* is unique in having anterodorsal process of lacrimal longer than height of preorbital process, and a thick, shelf-like thickening on the lateral surface of surangular ventral to the groove between the anterior surangular foramen and the insert for the uppermost intramandibular process of the dentary. Two other characters are only known in *Murusraptor*; sacral ribs hollow and tubelike; short ischia distally flattened and slightly expanded dorsoventrally. These characters are equivocal because they are unknown in other members of the clade. Also, the following combination of diagnostic characters was obtained after running the phylogenetic analysis using TNT [42]: Character 95, basipterygoid processes of the basisphenoid located anteroventrally, with basisphenoid recess opening posterodorsally (also present in coelophysids); Character 98, basisphenoid with a shallow embayment indentation between basal tubera and basipterygoid processes (also present in *Cryolophosaurus* and basal theropods); Character 216, rather straight chevrons (reversal to the plesiomorphic condition).

### Description

**Fig 3:**
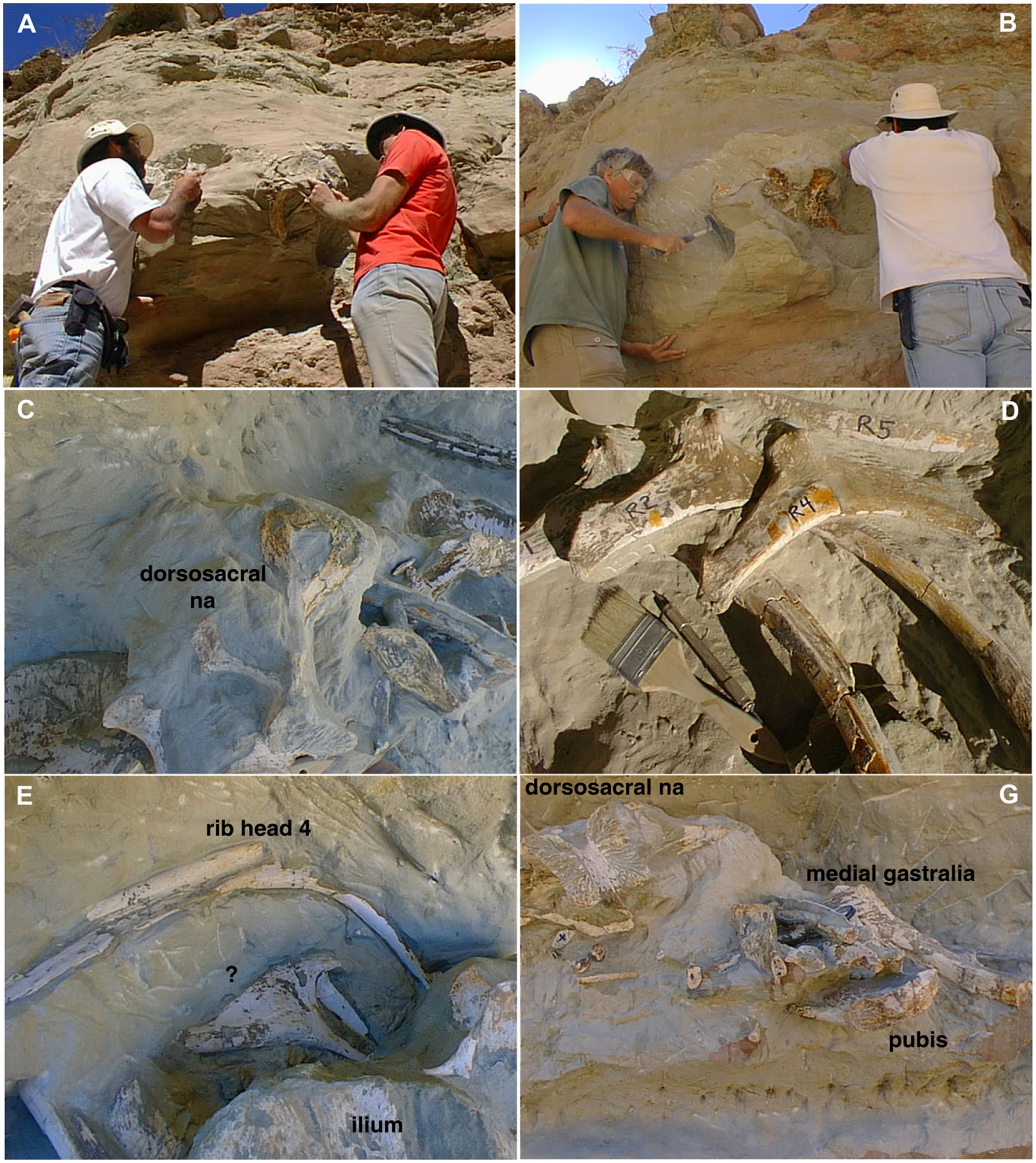
Field photos of the excavation of MCF-PVPH-411 (*Murusraptor barrosaensis*). A and B, the authors excavating the right ilium. C-F, different appendicular elements in their original burial positions before collection.

The holotype specimen of *Murusraptor barrosaensis* (MCF-PVPH-411) was found preserved in a sand-filled channel deposit (Fig 3).

It was mostly disarticulated, although the bones had remained closely associated with each other, and included both large and small elements. Most collected ribs were in natural sequence parallel to each other, suggesting the specimen was partially articulated when buried. Clay nodules were mixed amongst the bones. The orientations of the ribs and pubes, and the distribution of bones suggest that the animal was lying on its right side. Bones were stacked in as many as four layers. The right ilium was standing vertically upside down. The complete left ilium is the only element that was predepositionally broken; the preacetabular blade had snapped across the top of a large dorsal vertebra, and was separated slightly from the rest of the ilium. Incompleteness of the other bones is erosional. Several bones appear to have been bored by dermistids [43–44], which left characteristic trails of osseous fragments in the sediments. Parts of the skeleton showed pathologic damage.

#### Skull

None of the bones from the anterior parts of the skull or mandibles was recovered, although 31 teeth were found. The remaining cranial bones (especially the pterygoid) suggest that the skull was elongate, and probably had a narrow snout. This is a conclusion that had been reached independently for *Megaraptor* [35], whereas there are reports on the gracility of the dentary of *Australovenator* [29,36].

**Fig 4:**
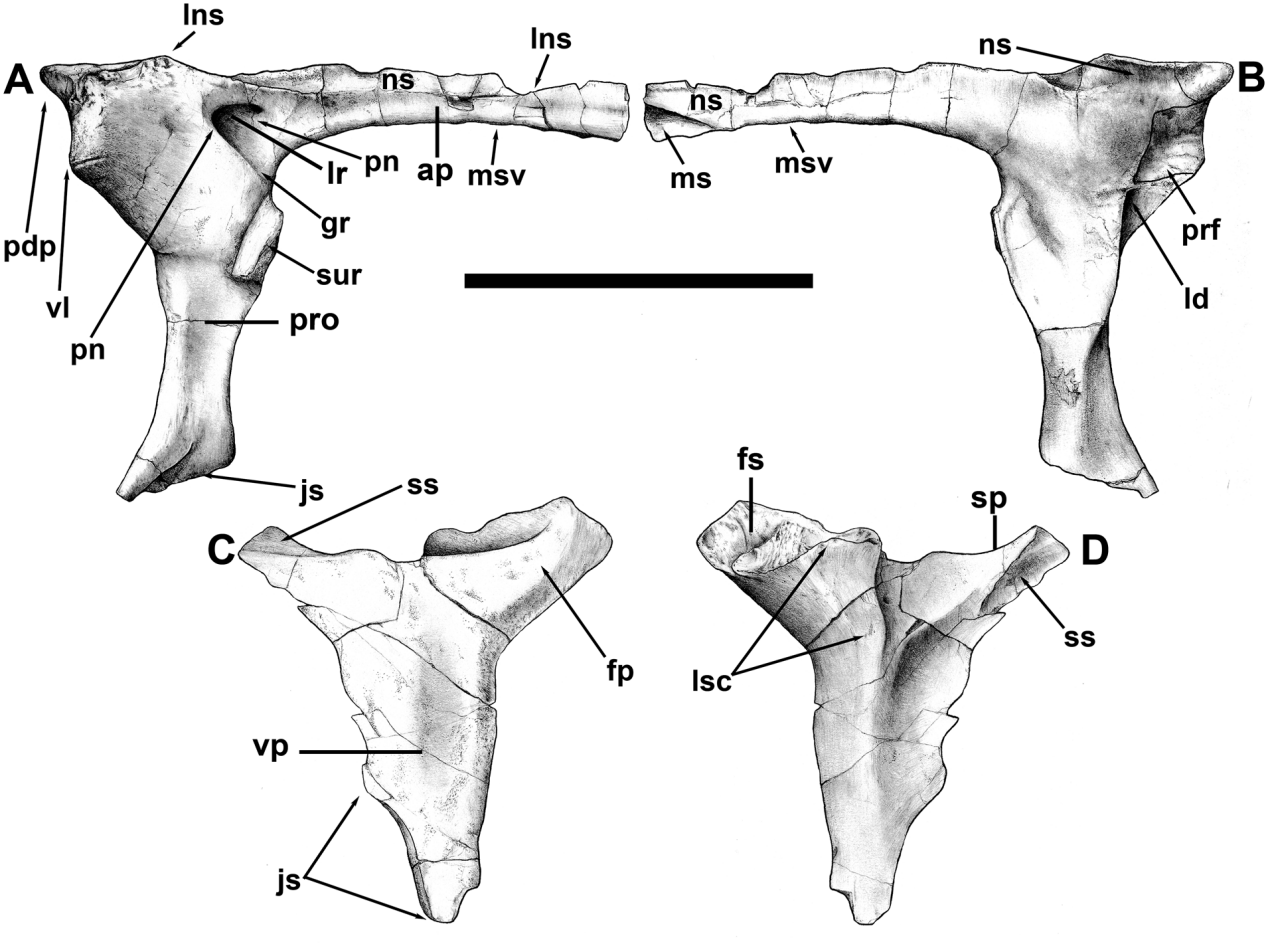
Right lacrimal and postorbital of *Murusraptor barrosaensis*, holotype, MCF-PVPH-41. Lacrimal in lateral (A) and medial (B) views. Postorbital in lateral (C) and medial (D) views. Abbreviations: ap, anterodorsal process; fp, frontal process; gr, groove from pneumatopores to antorbital fenestra; js, jugal suture; ld, lacrimal duct; lns, nasal suture limit; lr; lacrimal recess; lsc, laterosphenoid contact; ms, maxillary suture; msv, posterior limit of ventral contact with posterodorsal process of maxilla; ns, nasal suture; pdp, posterodorsal process; pn, pneumatopores; prf, prefrontal contact; pro, preorbital process; sp, squamosal process; ss, squamosal suture; sur, anterior tip of surangular; vl, ventral limit of prefrontal in lateral view; vp, ventral process. Scale bar: 10 cm.

The triradiate right lacrimal is almost complete (Fig 4A and 4B). It is “T” shaped (unlike most theropods in which the lacrimal is shaped like an inverted “L”), although the posterodorsal process is relatively short. In most theropods the anterodorsal and preorbital processes are subequal in size, whereas in some theropods, including abelisaurids, the anterodorsal process is significantly shorter than the preorbital process.

Both preorbital and anterodorsal processes are gracile, fragile and thin. The preorbital process, measured from the top of the bone, is 127 mm high as preserved, and is perpendicular to the anterodorsal process as in most theropods. The lacrimal as preserved measures 171 mm from the back of the bone, which is at least 30% longer than the dorsoventral length. The anterodorsal process, measured from the back of the lacrimal recess, is about 10% longer than the height of the preorbital process. This suggests the snout in *Murusraptor* was long, low, as in *Megaraptor* [35]. The posterodorsal corner of the antorbital fenestra was as high as the dorsal margin of the orbit as in most theropods. A relatively small (13.5 mm long, 4 mm high) lacrimal recess connects to the antorbital fenestra by means of a shallow, smooth-surfaced canal with elongate, posterodorsally inclined ridges. Pneumatopores within the lacrimal recess extend anteriorly into the anterodorsal process and posteriorly into the thickest part of the bone above the orbital margin. The posterior margin of the preorbital process is concave in lateral view, and formed the anterior margin for the orbit. This bar is less than 10 mm thick mediolaterally at all levels, and there is no ventromedial reinforcing bar of bone the way there is in most theropods. The preorbital process differentiates ventrally into two sheets of bone for its contact with the jugal. The posterolateral process follows the orbital margin and overlaps the jugal, whereas the anteroventral sheet would have been overlapped laterally by the jugal as in most other theropods. Similar to abelisaurids [45], there is nothing to indicate that the antorbital fossa extended onto this part of the preorbital bar as it does in carcharodontosaurids, tyrannosaurids and the majority of theropods.

The posterodorsal corner of the lacrimal is lateromedially thick, and the rugose lateral surface curves around onto the dorsal surface of the skull. A longitudinal groove isolates a posteromedial dorsal surface for the overlapping contact of the nasal. This surface is at the same level as the floor of the nasal suture of the frontal. The lacrimal does not contribute to either a preorbital horn (such as found in *Albertosaurus*, *Allosaurus*, *Baryonyx* and other theropods) or a prominent anterior ridge that extends onto the nasals (like those of *Daspletosaurus* or *Giganotosaurus*). The posterior margin of the posterodorsal region of the lacrimal has ridges and projections that plug into complementary structures on the anterodorsal surface of the prefrontal. A posterior, finger-like extension of the lacrimal on the dorsolateral edge of this crescentic sutural surface plugs into a depression in the prefrontal, and the mesial edge of the suture continues onto the medial surface of the lacrimal as a butt-joint for the anteroventral process of the prefrontal. This anteroventrally tapering contact terminates just above the lacrimal duct. The posterodorsal edge of the medial surface of the lacrimal in front of the prefrontal has a 15 mm long, triangular contact with the frontal.

Below the prefrontal suture of the lacrimal, the posteromedial margin of the preorbital bar is indented. This depressed area is penetrated by one (possibly two) lacrimal ducts, which emerge on the anteromedial surface of the preorbital bar near its junction with the anterodorsal ramus.

The anterodorsal process of the lacrimal has a pronounced, elongate dorsomedial sutural surface for the nasal. At the front, the lacrimal expands slightly dorsoventrally. Below this region is a ventral suture, tapering posteriorly, for contact with the maxilla. There is another tapering sutural contact on the medial surface in this region, presumably for another process of the maxilla. The anterodorsal process curves anteromedially in dorsal view, showing that the snout narrowed somewhat in front of the orbits.

**Fig 5:**
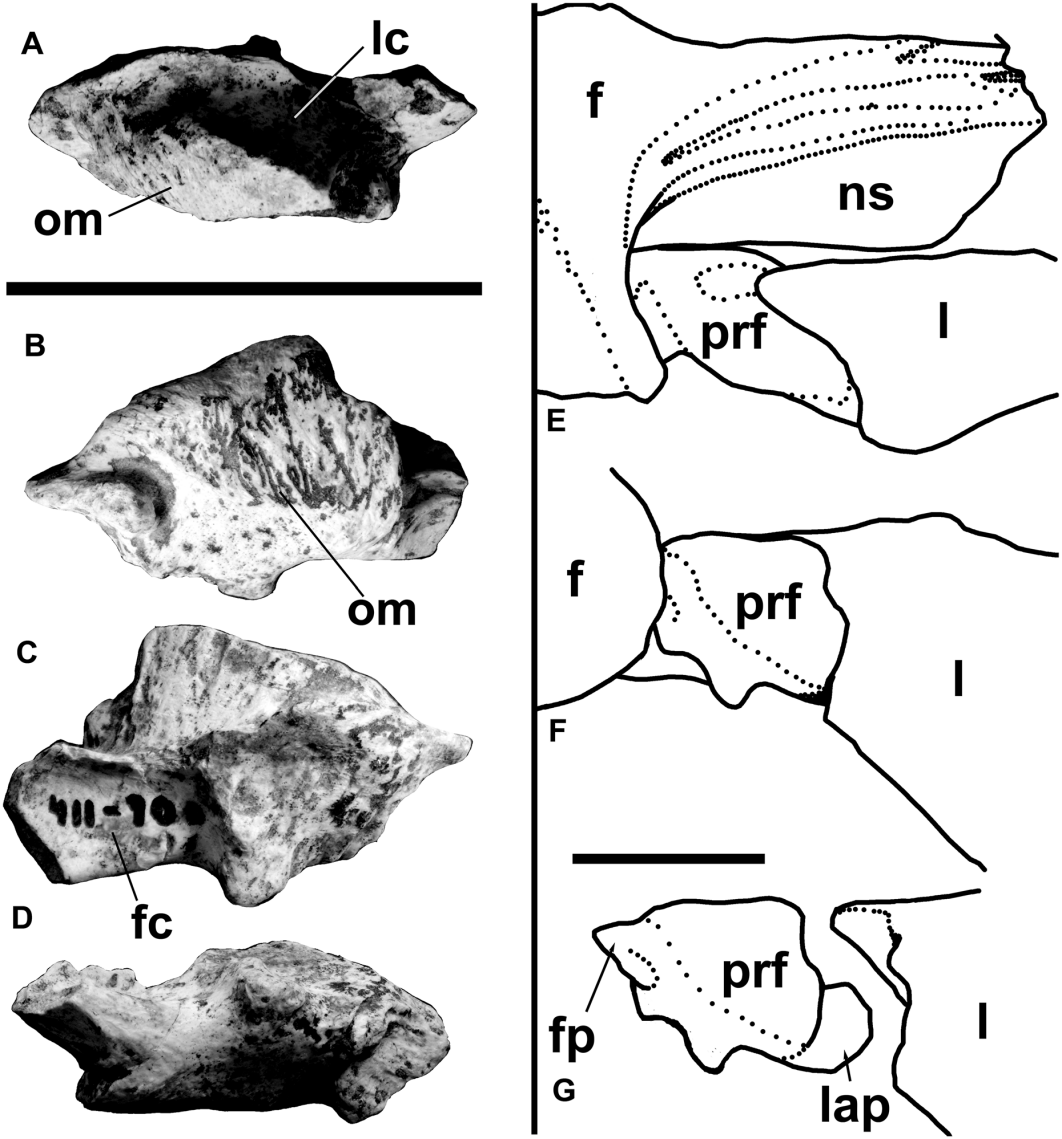
Right prefrontal of *Murusraptor barrosaensis*, holotype, MCF-PVPH-411. Prefrontal in dorsal (A), lateral (B), medial (C) and ventral (D) views. Prefrontal in articulation with frontal and lacrimal in dorsal (E) and lateral views (F). G) lateral view of prefrontal separated from the lacrimal. Abbreviations: f, frontal; fc, frontal contact; fp, frontal process of prefrontal; l, lacrimal; lap, lacrimal process of prefrontal; lc, lacrimal contact; ns, nasal suture of frontal; om, orbital margin; prf, prefrontal. Scale bars: 10 cm.

The prefrontal (Fig 5) is a small, separate element that contacts the lacrimal, nasal and frontal bones. The prefrontal is a separate element as it is in many of the Triassic and Jurassic theropods, including *Acrocanthosaurus* [46], *Allosaurus* [47], *Baryonyx* [48], *Coelophysis* [49], *Dilophosaurus* [50], *Herrerasaurus* [51], *Irritator* [52], *Sinraptor* [53] and *Zupaysaurus* [54–55]. Amongst more advanced theropods, the prefrontal seems to fuse with the lacrimal in most families, with the notable exceptions of ornithomimids and tyrannosaurids, both of which retain the more primitive condition of separate prefrontal wedged between the lacrimal, frontal and nasal.

Although it is similar in size and shape to the prefrontals of *Baryonyx* [48] and *Irritator* [52], there is no evidence for a postnasal fenestra such as has been inferred for these genera. The rugose lateral surface of the prefrontal forms part of the orbital margin above which the prefrontal would have been covered by the nasal. The prefrontal has a deep trough on its dorsal surface to embrace the lacrimal. A thin process extends anteroventrally along the medial surface of the preorbital bar of the lacrimal. The distal end of this process, although incomplete, would have extended almost to the lacrimal duct. The prefrontal of *Aerosteon* also has a short ventral process [26]. Posteriorly, the prefrontal has a complex system of contacts for the frontal. A thin, tapering posterior process separated by a deep groove from the main part of the prefrontal, plugs into the frontal. This is similar to at least some tyrannosaurids, which can also have a distinct process that plugs into a socket in the frontal [56]. Medially there is a tall, flat, almost vertical, parasagittal contact for the nasal process of the frontal.

The postorbital is complete except for the distal tip of the ventral process (Fig 5C and 5D). It is almost identical to the postorbital of *Orkoraptor* [28]. The bone has an upturned frontal process as in dromaeosaurids [57–58], although it is different in that the squamosal process rises just as high as the postorbital process. The frontal suture is complex and elongate as in tyrannosaurids and other large theropods. The dorsal surface of this process is excavated to form the anterolateral floor of the supratemporal fossa. The squamosal process has a dorsolateral, anteriorly tapering suture for the anterodorsal prong of the squamosal, and a larger ventromedial suture for the anteroventral prong of the squamosal. The ventral process is relatively long, above the top of the anteroventrally sloping suture for the jugal. The outer surface of the postorbital is smooth, whereas the inner surface is reinforced by a Y-shaped system of ridges. Fundamentally, it is the same as the postorbital of *Aerosteon* [26]. However, the contact of the postorbital with the braincase of *Murusraptor* demonstrates that what they interpreted in *Aerosteon* as the articular surface for the frontal includes also the contact with the laterosphenoid.

The braincase is intact and all of the bones are tightly sutured together. However, most of the sutures are still visible, indicating that this was not a fully mature animal. Still, it is larger and more mature than the braincase of an immature *Megaraptor* recently described [35]. The juvenile *Megaraptor* braincase is not coossified with the frontal, and is not as complete. Furthermore, it appears that the specimen is about 25% smaller than MCF-PVPH-411. Nevertheless, there are many similarities that show the close relationship between these animals.

**Fig 6:**
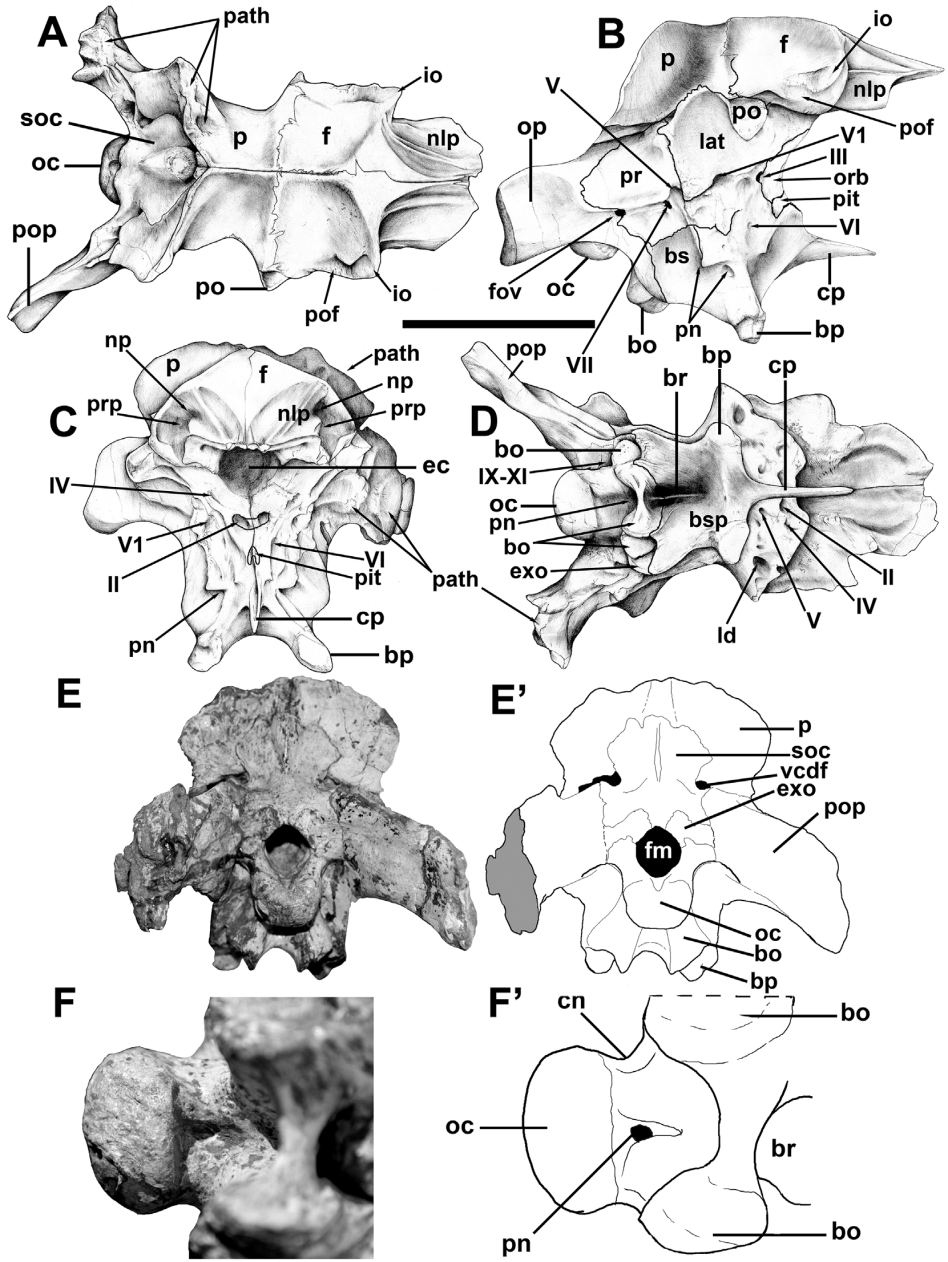
Braincase of *Murusraptor barrosaensis*, holotype, MCF-PVPH-411. Braincase in dorsal (A), lateral (B), anterior (C), ventral (D) and posterior (E,E’) views; occipital condyle in ventral view, photography (F) and line drawing (F’). Abbreviations: bo, basioccipital; bp, basipterygoid process; bs, basisphenoid; bsp, basipterygoid; br, basisphenoidal recess; cn, condylar neck; cp, cultriform process; ec, endocranial cavity; exo, exoccipital-opisthotic; f, frontal; fm, foramen magnum; fov, fenestra ovalis; io, interorbital slot between prefrontal/lacrimal and postorbital; n/p, nasal/prefrontal contact; lat, laterosphenoid; np, nasal pit; oc, occipital condyle; op, opistotic; orb, orbitosphenoid; p, parietal; path, pathology; pit, pituitary fossa; pn, pneumatopore; po, postorbital contact; pof, postorbital suture of the frontal; pop, paraoccipital process; pr, prootic; prp, prefrontal pit; soc, supraoccipital; vcdf, vena capitis dorsalis foramen; II, second cranial nerve exit; III, third cranial nerve exit; V, trigeminal (fifth cranial nerve) exit; V1, ophthalmic branch of the trigeminal exit; VI, sixth cranial nerve exit; IX-XI, exit for ninth to eleventh cranial nerves. Scale bar: 10 cm.

The circular foramen magnum of MCF-PVPH-411 is rimmed by basioccipital, exoccipitals and supraoccipital, and has a diameter of 25 mm. The same measurement in the juvenile *Megaraptor* is about 19 mm [35].The occipital condyle is kidney-shaped in distal view, 35 mm wide, and 25 mm high on the midline. It is formed mostly by the basioccipital, which also takes part in the floor of the neural canal. Many of the cranial bones at the back of the braincase on the left side are pathologic, and as a consequence the skull is asymmetrical in posterior view. This is especially true for the nuchal crest of the parietal, the prootic, and the opisthotic-exoccipital. There are two holes anteroventral to the nuchal crest, which might conceivably be puncture wounds from the teeth of another theropod. The left paroccipital process is pathological, and is 40% shorter (Fig 6D) than the right one. However, it is 31 mm mediolaterally thick distally, compared with a maximum distal thickness of 12.8 mm on the right side. The right prootic tapers posteriorly to overlap the base of the opisthotic. However, the pathological left prootic-opisthotic contact is an open window, up to 15 mm across, into the otic region. The distal end of the prootic had not ossified at the time of death, and the surfaces of the prootic and opisthotic adjacent to this pathological opening were pitted and grooved. The animal clearly had a serious infection within the left adductor fossa adjacent to the braincase.

The frontals are 111 mm long as preserved (Fig 6A–6C). Each one is about 54 mm wide between the interfrontal suture and the interorbital slot (between the contacts with the postorbital and lacrimal). The lateral margin of the frontal behind the interorbital slot is parallel to the midline of the skull, giving it a quadrangular shape. In shape, each is remarkably similar to an isolated frontal recovered with the juvenile *Megaraptor* [35], although it is only about two thirds the length and width of MCF-PVPH-411. All of the characteristic landmarks are shared by the frontals of the two specimens, although the juvenile *Megaraptor* frontal narrows between the orbits to about 85% of its maximum width posteriorly, whereas the two widths are the same in *Murusraptor*. The change in shape could be attributed to their difference in size, however, as ontogenetically the frontals of juveniles are triangular in tyrannosaurids, but more quadrangular in adults [56].

Anterior to the orbital slot, the frontal of MCF-PVPH-411 narrows abruptly to about 35 mm, and stays this wide almost to its anterior extent. Most of the dorsal surface of the anterior part of the frontal was covered by the nasal, and this sutural surface is continuous laterally with a nearly vertical plate of bone (Fig 6A and 6B) that contacted the prefrontal and lacrimal bones. This region of the bone is relatively thinner in the juvenile *Megaraptor* [35]. Only a thin, anteriorly tapering section of the frontal would have been exposed dorsally. The nasal-lacrimal-prefrontal sutures in MCF-PVPH-411 terminate posteriorly (Fig 6A–6C) in a dorsoventrally tall (35 mm laterally) face. An anteriorly facing pit in this wall undercuts the dorsal surface of the frontal medial to the orbital slot, and housed the posterior end of the prefrontal. This is similar to the condition in tyrannosaurids. A second more medial pit presumably received a posterolateral process from the nasal. The extensive nasal suture, defined posteriorly by the high wall of bone, is reminiscent of the frontals of crested duckbilled dinosaurs, and suggests that there may have been a nasal crest in this dinosaur. The wall of bone extends above the lacrimal and prefrontal, which also suggests that this crest may have covered the dorsal surfaces of these two bones above the orbit. However, it should be pointed out that the juvenile *Megaraptor* [35] also shows a disparity in height of the dorsal surfaces of the frontal anterior and posterior to the ridge that defines the front of the supratemporal fossa. The disparity is not as pronounced in the juvenile specimen, but that could be ontogenetically controlled. The nasal of the juvenile *Megaraptor*, however, seems to be uniformly thick along its length and shows no sign of developing a crest.

The frontal of MCF-PVPH-411 is dorsoventrally thick anterior to the adductor fossa, and is 35 mm thick on the midline. A ridge extends anterolaterally from this thick area to the orbital notch, forming a rather poorly defined border of the temporal fossa. The fossa is shallow but extensively covers the back of the frontal. On the midline, the frontals form a broad, low and poorly defined ridge that extends posteriorly into the sagittal crest of the parietal. The postorbital suture is complex, overlapping the posterodorsal margin of the frontal posteriorly, and plugging into a laterally facing socket anteriorly, as in tyrannosaurids. There is a low ridge along the almost transverse frontoparietal suture on the floor of the adductor fossa that is most pronounced medially. In ventral view (Fig 6D), there is a transversely-ridged sutural surface along the medial side of the orbital ridge. This extends anteromedially into a depression that marks the extent and shape of the unpreserved sphenethmoid.

The parietals are unfused, and are 33 mm long along the midline, which is less than a third the length of the frontal. The paired parietals are 100 mm in transverse width along the frontal-laterosphenoid sutures, 54 mm at their narrowest point between the supratemporal fenestra, and 120 across the maximum expanse of the nuchal crest. The supratemporal fossae of the paired parietals approach the midline but are separated by a narrow but low ridge. The parietals form the broad nuchal crest, which is the same height as the sagittal ridge between the supratemporal fossae, and is extensively exposed on the occiput above the paroccipital processes. Neither the sagittal ridge nor the nuchal crest resembles the tall, equivalent structures in tyrannosaurids.

In lateral view, the interdigitating contact between the laterosphenoid and prootic inclines posterodorsally from the trigeminal foramen. It meets the nuchal crest of the parietal anterior to the parietal-supraoccipital contact (Fig 6B). The laterosphenoid formed the anterior and part of the dorsal margin of the major, lateral opening of the trigeminal nerve. There is an extensive dorsolateral facet for the postorbital. Anteromedial to the facet is a deeply rugose, interdigitating sutural contact with the frontal. A deep pit, the bottom of which seems to be penetrated by a foramen, possibly for the fourth cranial nerve, invades the anteroventral surface of the postorbital process. Anteroventrally, the laterosphenoid forms a thick, sculptured ridge in front of the trigeminal foramen. There is no indication of a groove extending forward for the ophthalmic branch of the trigeminal. Instead, it seems that this branch was encased in the bone as in troodontids tyrannosaurids and birds [53]. Not surprisingly, a large foramen exits the braincase anterior to this ridge, and presumably carried the ophthalmic branch.

Although there are no evident sutures, there is a medial orbitosphenoid ossification anterior to the paired laterosphenoids. In anterior view (Fig 6C), this partially separates the foramina for the orbital nerves and forms the floor of a single medial olfactory exit. The ossification is formed by a pair of bones, which are still separate between the openings for cranial nerves I and II. Posteriorly, the orbitosphenoid forms the anterior margin of the opening for cranial nerve III, and ventrolaterally it roofs over the pituitary fossa. Dorsolaterally, it forms the anterior margin of a small opening that may be the exit for the fourth cranial nerve.

The prootic forms the dorsal and posterior margins of the trigeminal foramen, and the dorsal and anterior margins of the fenestra ovalis. The prootic extended posterodorsally for a short contact with the parietal and then tapers distally along the anterior face of the paroccipital process (exoccipital-opisthotic).

The exoccipital-opisthotic complexes (otoccipitals) of MCF-PVPH-411 are separated from each other by the supraoccipital above the foramen magnum and by the basioccipital below. This is also the case, at least for the separation by the supraoccipital, in the juvenile *Megaraptor* specimen [35]. The paroccipital process is relatively wide (extends 85 mm laterally from the midline of the skull on the right side) and shallow (43 mm distally). It turns slightly downwards distally, but the distal end is level with the occipital condyle. The ninth to eleventh cranial nerves, and at least two branches of the twelfth cranial nerve emerge through two foramina in a depression (paracondylar recess) between the occipital condyle and the paroccipital process on the left side. On the right side, an extra foramen with a diameter of 2 mm emerges from the exoccipital some 14 mm lower. There is no evidence of pneumatopores in the paracondylar depression. Like most theropods, the exoccipital extends ventrally until it is even with the lowest level of the basal tuber.

The basioccipital composes the largest part of the occipital condyle and makes a narrow contribution to the floor of the foramen magnum. Like the juvenile specimen of *Megaraptor*, there is a deep excavation of the condylar neck of the dorsal surface of the occipital condyle [35].The ventral surface of the condylar neck has a deep depression (the subcondylar recess) that is pierced by a medial pneumatopore (Fig 6E and 6F) as in the juvenile specimen of *Megaraptor* [35], carcharodontosaurids [14] and some tyrannosaurids [59]. Anteroventral to the condylar neck, the basioccipital forms a nearly vertical concave wall between the supporting columns of the basal tubera. The almost perpendicular relationship between the condylar neck and the basal tubera is different from the situation in carcharodontosaurids and sinraptorids [14], in which the angle is obtuse. The width across the basal tubera is 58 mm, which is transversely wider than the diameter of the occipital condyle as in the juvenile specimen of *Megaraptor* [35], dromaeosaurids [60], troodontids [58] and tyrannosaurids [61]. Ventrally, the basal tuber is unusual in that it is notched to form a pair of facets, one vertical and the other facing mostly ventrally (Fig 6D). A notch may have been present in the juvenile specimen of *Megaraptor* (Figure 7 in [35]) but the preservation does not appear to be good enough to show the pair of facets. A narrow process extends laterally to the outer margin of the basisphenoid on the left side, but appears to have been lost on the right side to expose the ridges of the sutural contact with the basisphenoid. A plate of bone between the basal tubera forms the posterior wall of the basisphenoid. This extended anterodorsally to form part of medial roof of the basisphenoidal recess (Fig 6D), completely encircling a pair of pneumatic foramina extending dorsoposteriorly into the body of the basioccipital.

The cultriform process of the basisphenoid-parasphenoid complex extends 55 mm anterior to the prominent basipterygoid processes. Twenty millimeters below the paired medial openings for the optic nerves, the dorsal surface of the basisphenoid is notched to form the posterior, lateral and ventral walls of the pituitary fossa. The trough-like dorsal surface of the process presumably clasped the cartilaginous interorbital septum. There is a shallow cultriform trough on the posteroventral surface of the process, which is different than the extensive depression in *Sinraptor* [53]. The basipterygoid processes are continuous across the midline in an anteroposteriorly thick, ventrally concave ridge. This forms the 35 mm high anterior wall of the basisphenoid recess. The recess is approximately twice as wide as it is anteroposteriorly long, and opens ventrally and only slightly posteriorly. It also extends posterodorsally into a pair of pneumatic foramina that invade the interior of the basioccipital.

The supraoccipital makes up 5.5 mm of the dorsal margin of the foramen magnum. The dorsal median ramus of the supraoccipital is constricted between the openings for the vena capitis dorsalis, but expands dorsally into an anteroposteriorly thick (19 mm) process with a transverse diameter of 16 mm. The width of the expansion is less than those of either the foramen magnum or the occipital condyle. The parietal extends 22 mm above its suture with the top of the supraoccipital, but the surfaces of both bones suggest there was a cartilaginous extension. Lateral to the midline ridge and above the exit for the vena capitis dorsalis, the supraoccipital tapers laterally. Ventrolateral to the level of the foramen for the vena capitis dorsalis, the lateral process of the supraoccipital extends 15 mm along the dorsal border of the paroccipital process.

**Fig 7:**
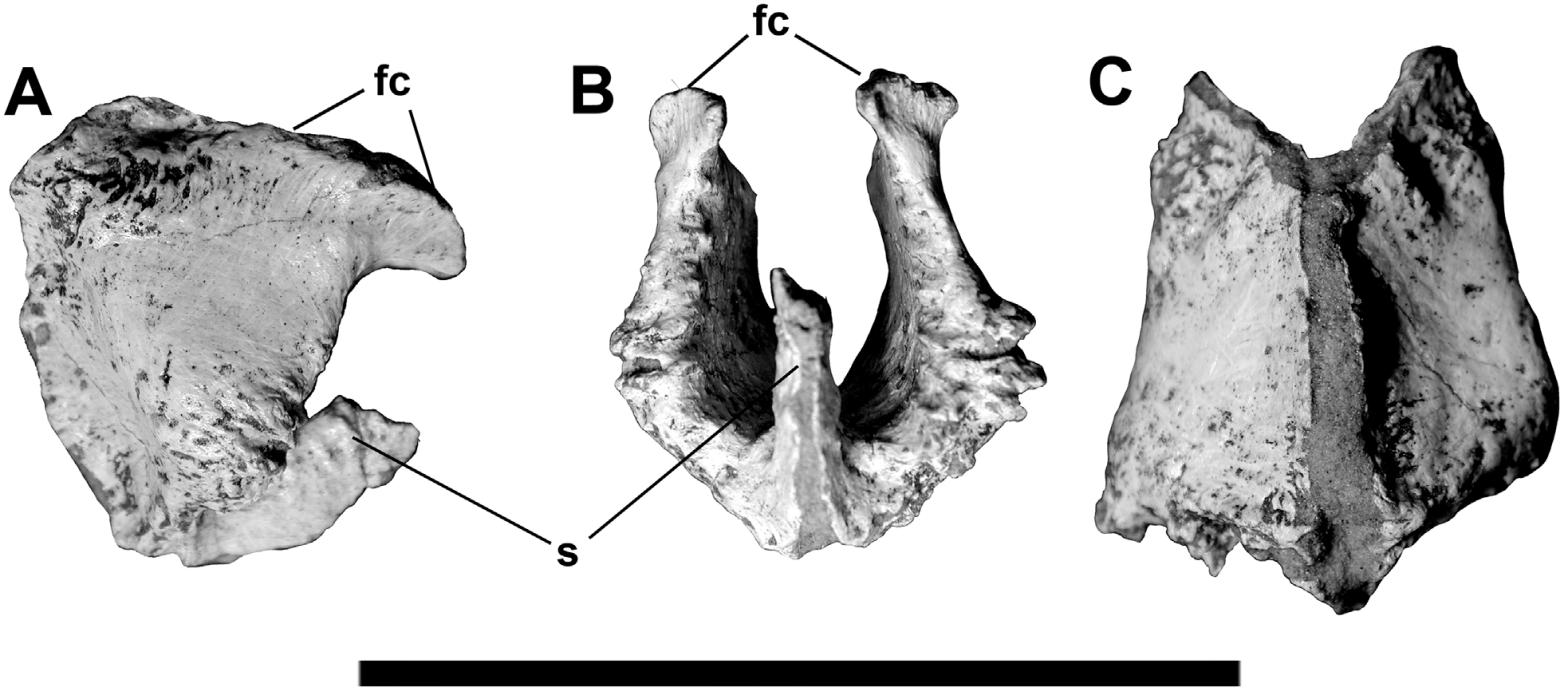
Sphenethmoids of *Murusraptor barrosaensis*, holotype, MCF-PVPH-411 in right lateral (A), anterior (B) and ventral (C) views. Abbreviations: fc, frontal contact; s, medial septum. Scale bar: 10 cm.

Both sphenethmoids were recovered together, although the midline suture between them had opened up ventrally (Fig 7). They contact a rugose ridge and the depression located on the anteroventral surface of the frontals. When they are are in contact with the paired frontals, there is a 12 mm (at closest point) gap between the sphenethmoids and the frontal/laterosphenoid/orbitosphenoid junction. There is a 25 mm tall (12 mm long anteroposteriorly) process on the midline separating the lower part of the olfactory bulbs. It is fused posteriorly, but the midline suture is visible anteriorly.

**Fig 8:**
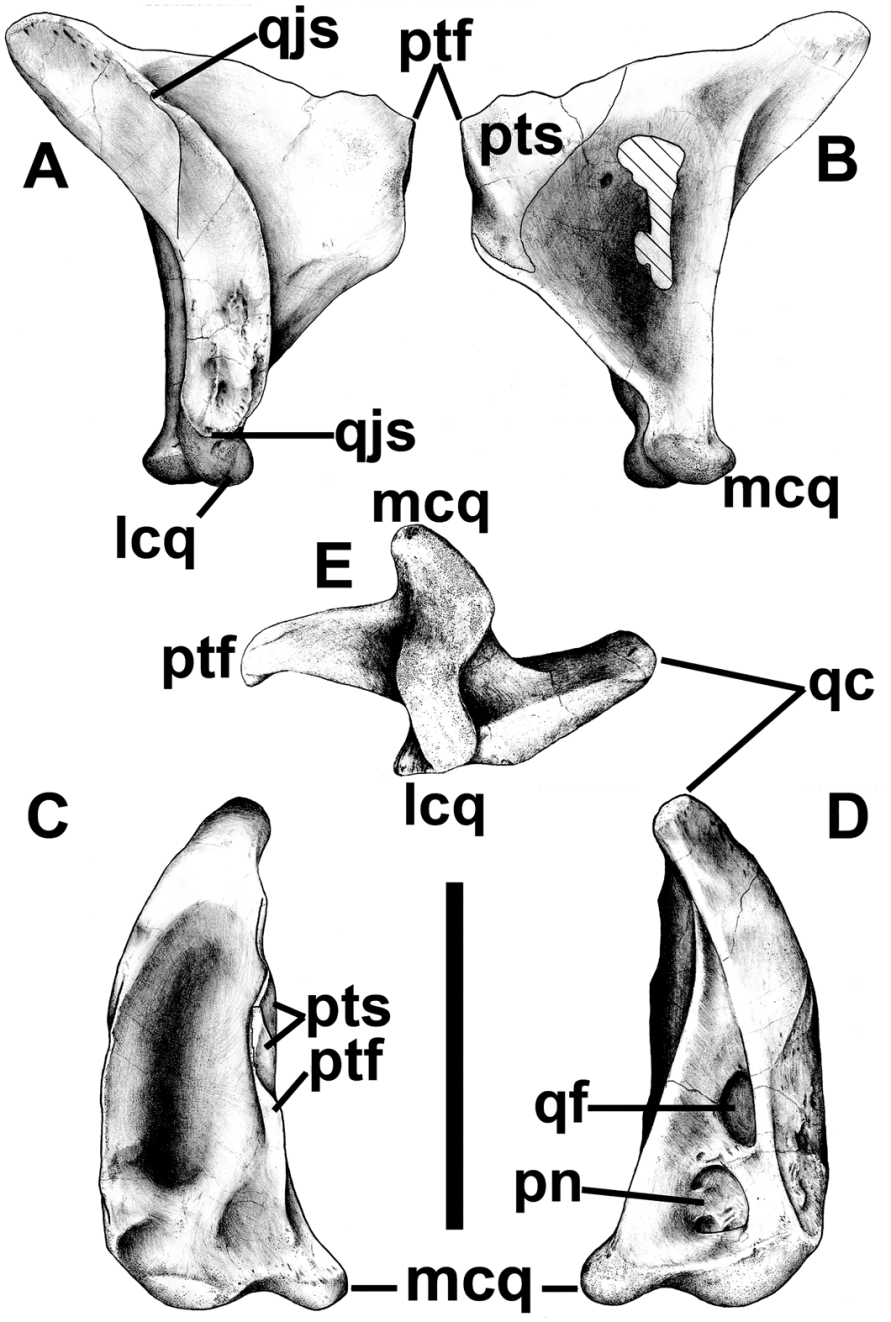
Quadrate of *Murusraptor barrosaensis*, holotype, MCF-PVPH-411 in lateral (A), medial (B), anterior (C), posterior (D) and distal (E) views. Abbreviations: lcq, lateral condyle of quadrate; mcq, medial condyle of quadrate; pn, pneumatopore; ptf, pterygoid flange; pts, pterygoid suture; qc, quadrate cotyle; qf, quadrate foramen; qjs, quadrate-jugal suture. Scale bar: 10 cm.

With a height of 149 mm, the quadrate is taller than the lacrimal (Fig 8). It is almost indistinguishable from that of *Aerosteon* [26] (Sereno et al. 2008), although the latter is 10% taller [26]. The quadratic foramen is completely surrounded by the quadrate, and is separated from the quadratojugal by a 5 mm thick bar of bone. This is similar to the condition in allosauroids where the quadratic foramina/fenestrae are virtually surrounded by the quadrates in *Acrocanthosaurus* [62], *Allosaurus* [47] and *Sinraptor* [53]. In contrast, the quadratic fenestra is bordered laterally by the quadratojugal in dromaeosaurids and tyrannosaurids. It is a relatively large opening (22.3 mm high), comparable with a tyrannosaurid quadratic fenestra in relative size, but relatively smaller than a dromaeosaurid one. The quadratojugal suture is more than 100 mm tall, and clearly indicates that the quadratojugal was tall and almost certainly contacted the squamosal. Unlike *Carnotaurus* and mature tyrannosaurids, there is no indication of fusion between quadrate and quadratojugal. The ventral part of the suture for the quadratojugal is concave and has at least two ridges. The anteroposterior length of the pterygoid flange is approximately 70% of its height. The quadrate is pneumatic like those of carcharodontosaurids and tyrannosaurids. The pneumatopore is on the posterior surface of the quadrate ventral to the quadratic fenestra. It is 17 mm wide and 19 mm tall; internally, it is subdivided into at least three more pneumatopores that extend the pneumatic cavity dorsally into the main shaft of the quadrate, ventrolaterally into the interior of the lateral articular condyle, and ventromedially into the medial condyle. The medial surface of the pterygoid process is deeply concave and seems to have housed a large air sack. Many holes pass through the pterygoid process here, although most of them may represent incomplete ossification of the thin wall. Anteroventrally, the medial surface of the process has a distinct sutural surface, reinforced by a thickening of the ventral margin, for its lower contact with the pterygoid. The pterygoid overlapped the medial surface of the quadrate above this distinct surface, and extended along most of the dorsomedial margin of the pterygoid process. Ventrally the two condyles are oriented anteromedially, and are separated by a relatively deep intercondylar groove.

**Fig 9:**
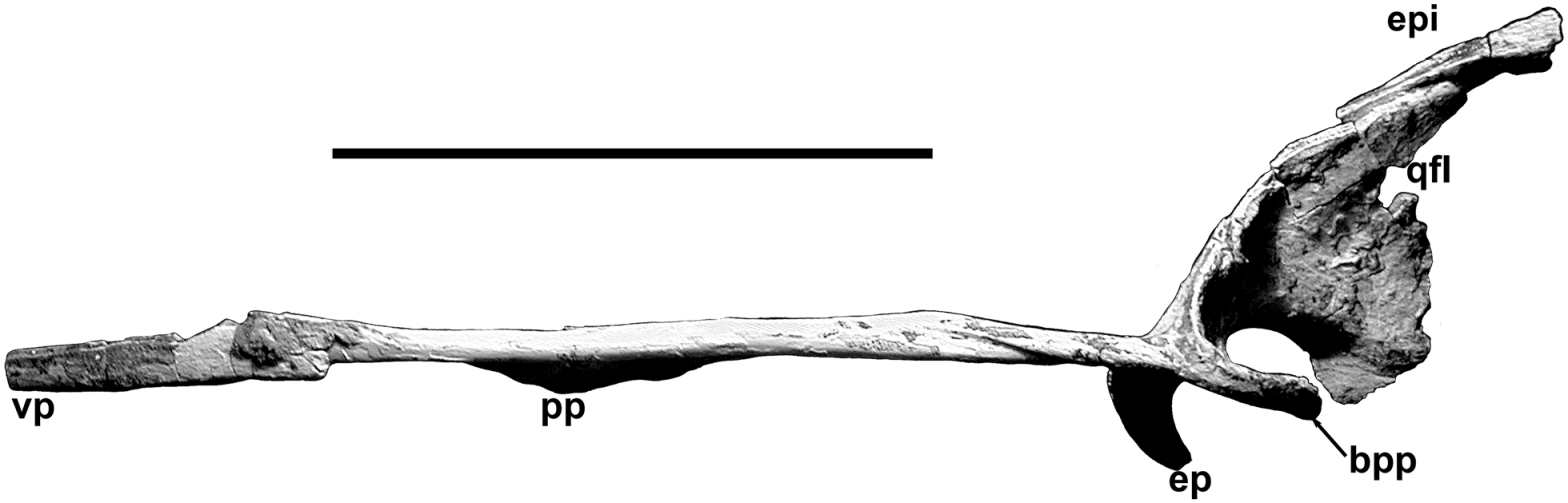
Pterygoid of *Murusraptor barrosaensis*, holotype, MCF-PVPH-411 in lateral view. Abbreviations: bpp, basipterygoid process; ep, ectopterygoid process; epi, epipterygoid; pp, palatal process; qfl, quadrate flange; vp, vomerine process. Scale bar: 10 cm.

Both pterygoids were recovered, and the right one is complete (Fig 9). It is a thin, delicate, complex structure. From the anterior end of the vomerine process, it extends 310 mm to the basipterygoid process, and 370 mm to the back of the quadrate process. The elongate, tapering anterior palatal ramus probably did not meet the opposite pterygoid on the midline, but would have been held in position anteriorly by the vomers. Ventral to the midline, the palatal processes of the pterygoids diverge ventrolaterally. There is a long, pronounced squamose suture with the palatine. The dorsolateral surface has a posteriorly tapering depression that probably is the sutural surface for the palatine. Anteriorly, this suture continues for most of the length of the pterygoid. As in *Sinraptor*, there was only a narrow region separating the palatine from the ectopterygoid. Here the pterygoid contributes several centimeters to the margin of the postpalatine fenestra. The base of the ectopterygoid (ventral) process has a lateral sutural surface for contact with the ectopterygoid. Ventrally, the ectopterygoid contact is restricted to the anterior margin of this ramus, but appears to have extended onto the anterior part of the medial surface as well. The quadrate ramus is a thin, curved sheet of bone, which is strengthened anteriorly by a medial ridge of bone that extends dorsally from the basipterygoid articulation. Ventrally, there is an 8 mm wide shelf of bone on the lateral surface beneath the contact with the quadrate. On the medial surface at the base of the quadrate ramus, there is a finger-like projection that folded around the basipterygoid process of the basisphenoid-parasphenoid complex. The curved dorsal margin of the quadrate ramus is notched for the contact with the epipterygoid, which appears to be lying against the posterolateral edge in this specimen.

Part of the right epipterygoid appears to be preserved close to its original position, but lies along the dorsal margin of the quadrate process of the right pterygoid (Fig 9). It is a slender, laterally compressed bone with cross-sectional diameters of 2.8 by 6 mm.

**Fig 10:**
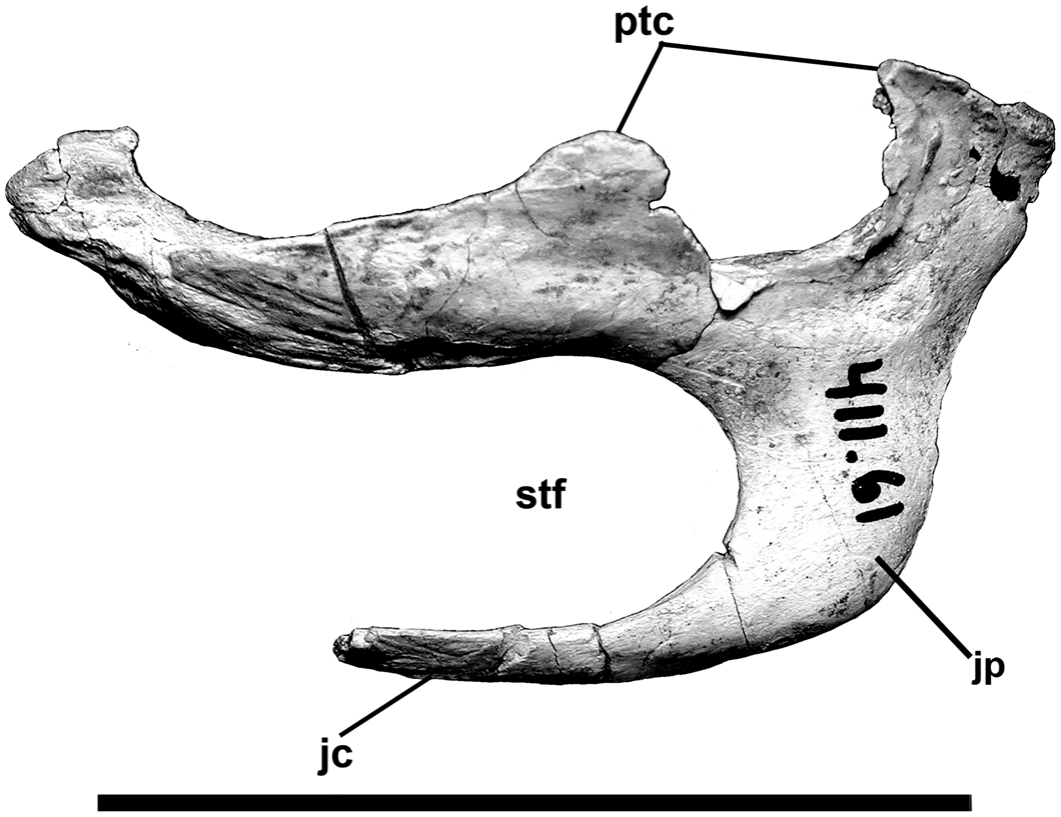
Ectopterygoid of *Murusraptor barrosaensis*, holotype, MCF-PVPH-411 in dorsal view. Abbreviations: jc, jugal contact; jp, jugal process; ptc, pterygoid contact; stf, subtemporal fenestra. Scale bar: 10 cm.

The elongate right ectopterygoid is 110 mm long, has a maximum width of 72 mm, and a jugal process that is 26 mm (Fig 10). The bone is relatively flat in contrast with the inflated, pneumatic ectopterygoids of large tyrannosaurids [56], but resembles those of most other theropods [53]. The ventral surface houses a deep well defined depression that extends through a small pneumatopore into the base of the hooked jugal process.

**Fig 11:**
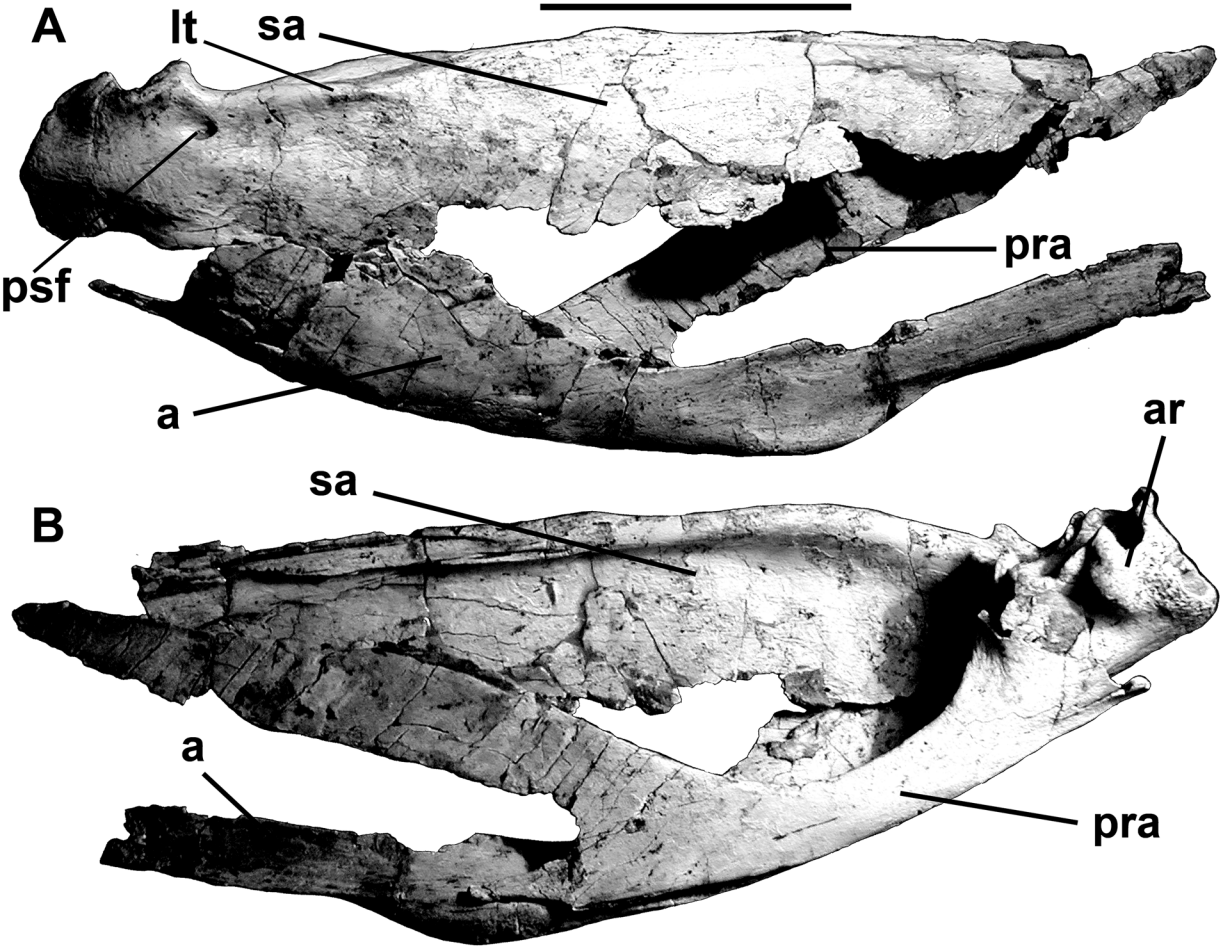
Articulated postdentary elements of *Murusraptor barrosaensis*, holotype, MCF-PVPH-411 in lateral (A) and medial (B) views. Abbreviations: a, angular; ar, articular; lt, lateral thickening; pra, prearticular; psf, posterior surangular foramen sa, surangular. Scale bar: 10 cm.

The bones from the posterior half of the jaw were recovered. Their elongate and lightly built structures suggest that the same would have been true for the anterior bones that were not found. When the recovered bones are put in articulation, the jaw is 120 mm deep at the level of the adductor fossa (Fig 11). In theropods, the dentary is usually about the same length as the surangular, which suggests the lower jaw was about 70 cm long. The shapes of the surangular, prearticular and angular indicate that the external and internal mandibular fenestrae were relatively large. The external mandibular fenestra was at least 12 cm long based on the finished margins of the angular.

**Fig 12:**
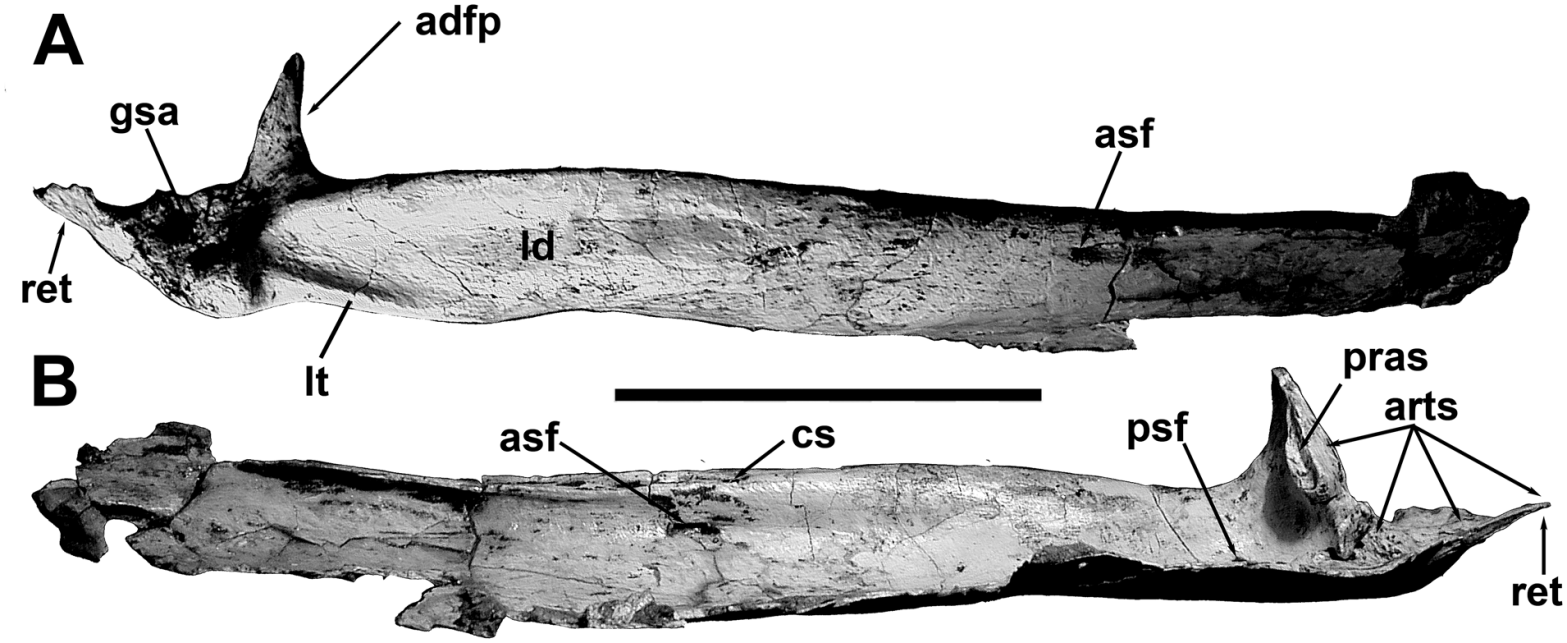
Surangular of *Murusraptor barrosaensis*, holotype, MCF-PVPH-411in dorsal (A) and medial (B) views. Abbreviations: adfp, posterior wall of the adductor fossa; arts, articular suture; asf, anterior surangular foramen; cs, back of coronoid suture; gsa, glenoid contribution of surangular; ld, longitudinal depression for insertion of the M. adductor mandibulae externus; lt, lateral thickening; pras, prearticular suture; psf, posterior surangular foramen; ret, retroarticular process. Scale bar: 10 cm.

The 370 mm long right surangular is complete (Fig 12), although the anterior tip is still attached to the lacrimal (Fig 4). The anterior surangular foramen (for cutaneous branches of the inferior alveolar nerve, [63] opens anteriorly into a shallow groove (Fig 12A) as in *Allosaurus*, *Monolophosaurus*, *Sinraptor* and other theropods. There is an extensive squamose suture for the angular along the outside of the thin ventral margin anteroventral to the glenoid (Fig 11A). The posterolaterally oriented posterior surangular foramen (7 mm diameter) is anterolateral to the glenoid, beneath a powerful lateral ridge; the ridge forms the lateral margin of the dorsal longitudinal depression for insertion of the *M*. *adductor mandibulae externus*. There is an elongate, immovable squamose suture for the coronoid along the anteromedial surface of the ridge. Posteriorly, the ridge extends into a medial process that delimits the posterior margin of the adductor fossa, contacts the prearticular medially, and forms part of the extensive sutural surface for the articular. Posterodorsally, the surangular makes a small contribution to the ridge separating the two depressions of the glenoid, and is shallowly depressed for its contact with most of the lateral condyle of the quadrate. The surangular extends as far posteriorly as the articular.

**Fig 13:**
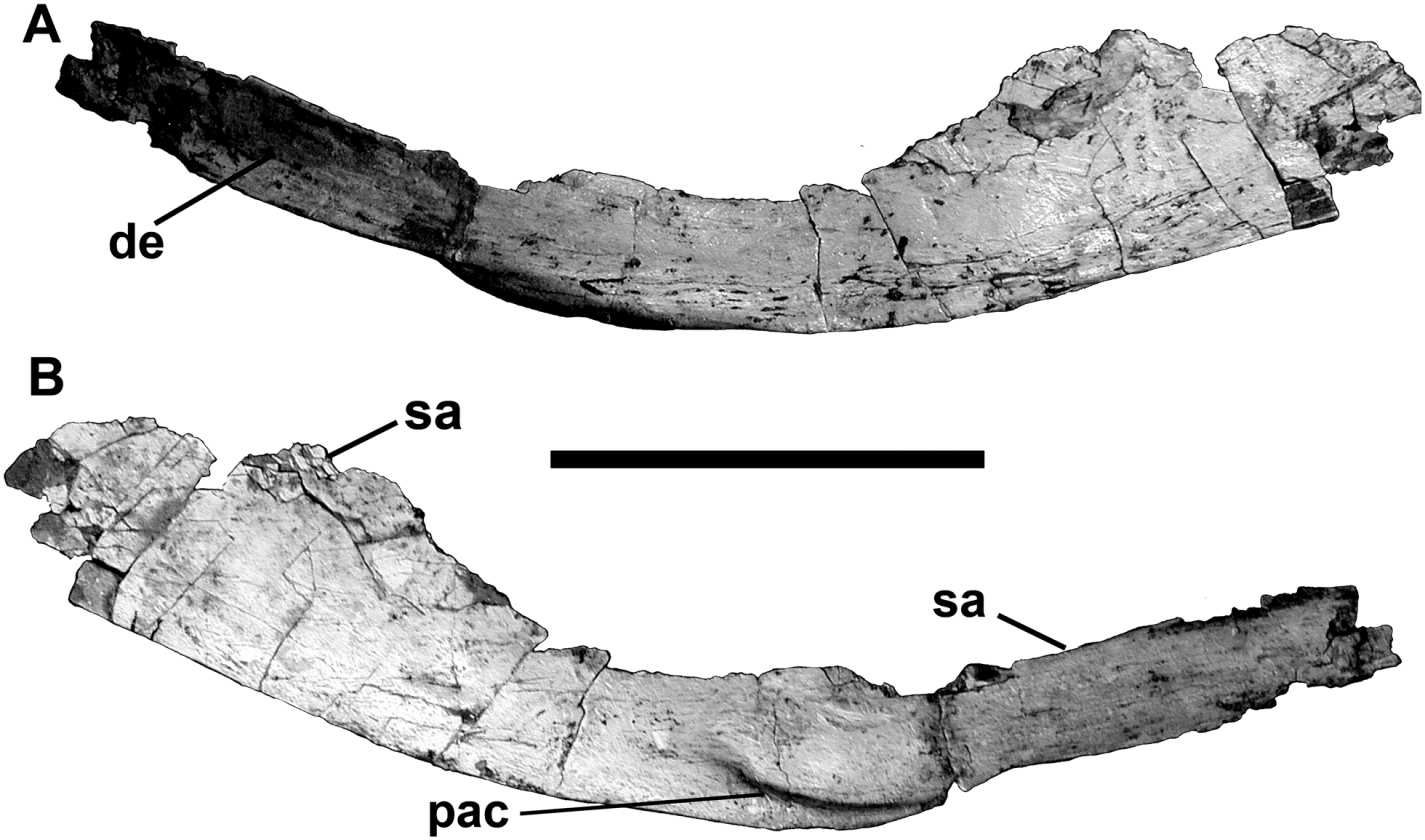
Angular of *Murusraptor barrosaensis*, holotype, MCF-PVPH-411 in lateral (A) and medial (B) views. Abbreviations: de, contact for dentary; pac, prearticular contact; sa, contact for surangular. Scale bar: 10 cm.

The angular is missing the tips of the anterior and posterior ends, but was approximately the same length as the prearticular (Fig 13). It is strengthened by a thick ventral margin for most of its length. Almost half of the bone would have been overlapped laterally by the dentary, but the contact surface is smooth, and sliding movement would have been possible (Fig 13A). The ventral edge of the angular is thickest where it forms the ventral margin of the jaw behind this point. On the medial surface of this region, there is an anteriorly tapering sutural contact for the front of the lower anterior ramus of the prearticular. Posteriorly, the angular laterally overlaps the surangular. Radiating ridges and grooves on the sutural surfaces show that this contact was immovable. Like most theropods, there is no other contact between the angular and surangular. The posterior end of the angular probably ended anterior to the level of the articular bone as in most theropods other than *Allosaurus*.

**Fig 14:**
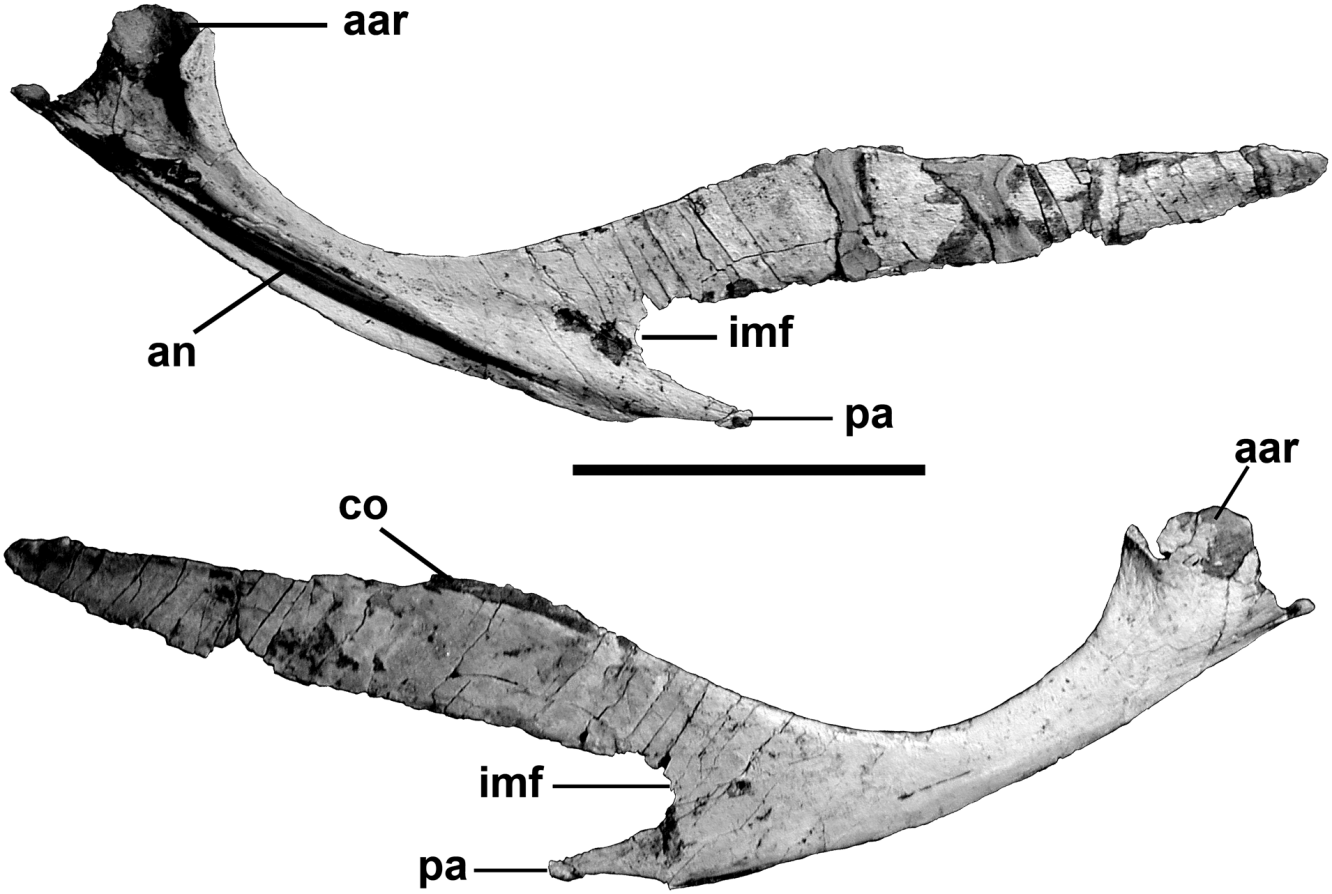
Prearticular of *Murusraptor barrosaensis*, holotype, MCF-PVPH-411in lateral (A) and medial (B) views. Abbreviations: aar, articulation for the articular; an, contact for the angular; co, contact for the coronoid; imf, posterior margin of the internal mandibular fenestra; pa, process for articular. Scale bar: 10 cm.

Posteriorly, the 365 mm long prearticular is strengthened by a ventromedial ridge that wraps around the medial surface and bottom of the articular (Fig 14). A posterolateral ridge wraps around the front of the anteromedial rim of the medial glenoid and contacts the medial process of the surangular. The angular rests in a longitudinal trough on the lower side of the shelf, but anteriorly this opens up ventrally and the prearticular loses its exposure on the lateral surface of the mandible. The lateral surface has extensive contacts with the articular and the postadductor process of the surangular (Fig 14A). Anteriorly the prearticular divides into two processes, one dorsal and one ventral to the internal mandibular fenestra (internal mandibular foramen [47]). The division in itself is not unusual, although the fenestra must have been quite large. The exact size, however, cannot be determined in the absence of the splenial. The ventral prong tapers to a point, and presumably contacts the angular anteriorly, whereas the dorsal prong is elongate and relatively slender. A longitudinal depression close to the anterodorsal margin is probably the contact for the coronoid.

**Fig 15:**
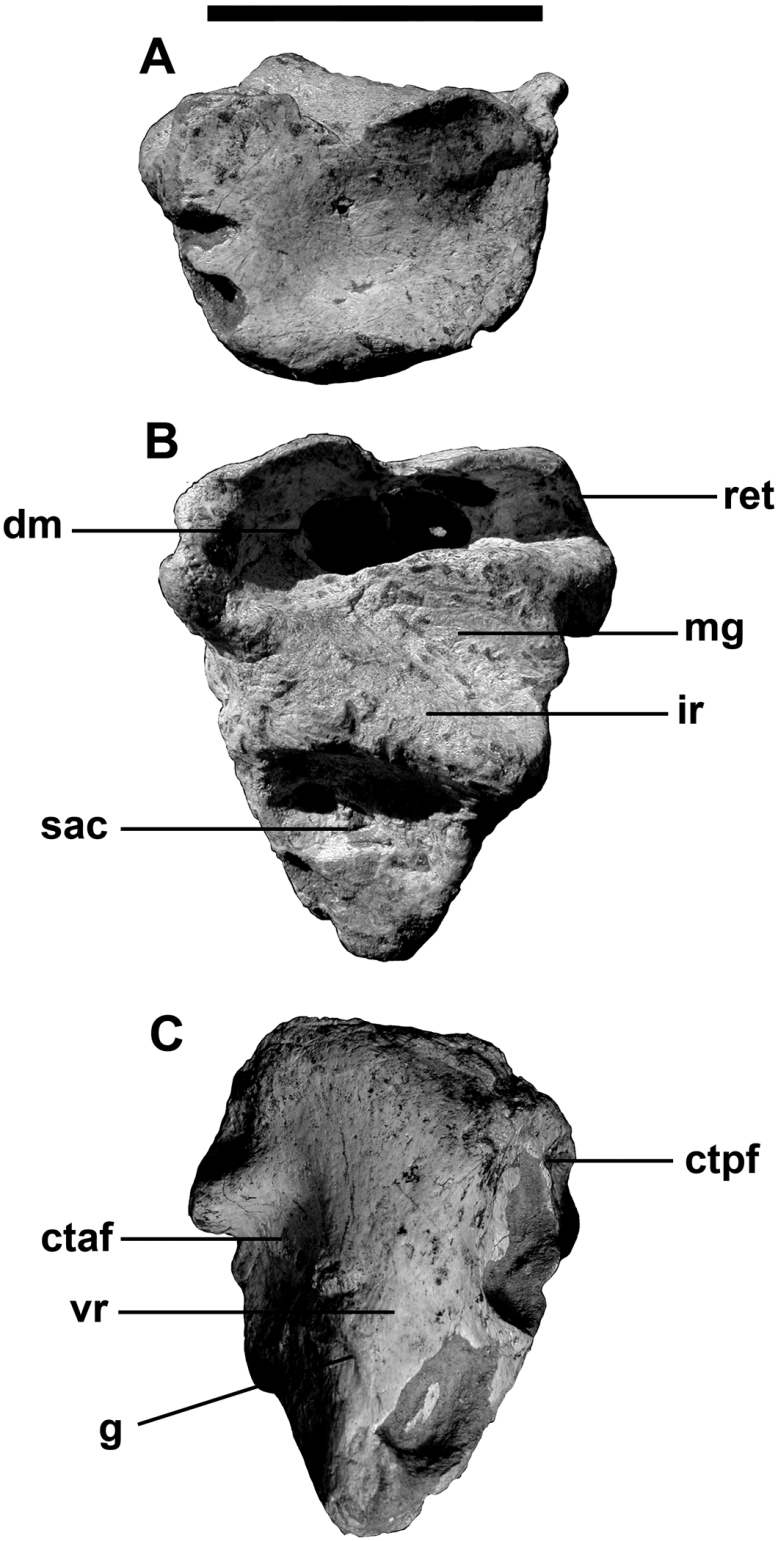
Left Articular of *Murusraptor barrosaensis*, holotype, MCF-PVPH-41. Articular in posterior (A), dorsal (B) and ventral (C) views. Abbreviations: ctaf, *chorda tympani* anterior foramen; ctpf, *chorda tympani* posterior foraman; dm, insertion area for *M*.*depressor mandibulae*; g, vascular groove; ir, interglenoid ridge; mg, medial glenoid; ret, retroarticular process; sac, surangular contact; vr, ventral ridge. Scalebar: 10 cm.

The medial glenoid and most of the interglenoid ridge for the jaw articulation are found on the triangular articular (Fig 15). The bone is somewhat more elongate than those of *Allosaurus* [47,64] and tyrannosaurids [65–67], even though the retroarticular process is less than a centimeter in length. The insertion for the *M*. *depressor mandibulae* occupied the dorsal surface of the retroarticular process, and continued anterodorsally onto the smooth, slightly concave posterior surface of the articular. Ventrally, the articular narrows between the surangular and prearticular posteriorly, and has a loose sutural contact with the lateral surface of the prearticular anteriorly. This ventral ridge is thick as in *Allosaurus*, and does not extend as far forward. Posteromedial to the interglenoid ridge behind the medial glenoid, there is a deep depression that contains the small posterior foramen of the *chorda tympani*, and three large pneumatic openings (Fig 15B). The whole body of the articular seems to have been filled by pneumatic sinuses and the thin walls of the bone have collapsed in at least three places on the contact surface for the surangular. These are not pneumatopores as they do not continue across the contact to invade the surangular. The thin walls have also collapsed to expose the sinus system along the medial edge of the *M*. *depressor mandibulae* insertion. Pneumatic articulars are also known in tyrannosaurids. The anteromedial surface of the articular is lightly scarred for its contact with the prearticular. The anterior foramen for the *chorda tympani* and posterior condylar artery [68] exits the articular near the posterior margin of this suture, and continues anteroventrally between the two bones in a groove on the sutural surface of the articular.

#### Dentition

Thirty-one teeth were recovered amongst the bones of the skeleton, although twenty-six are complete enough to provide measurable data (Table 1). Comparison of the teeth recovered suggests that there are many gaps in the sequence, and that there would have been many more functional teeth in the jaws of the animal when it was alive. Four premaxillary teeth were reported and as many as 17 maxillary teeth in the juvenile *Megaraptor* specimen (MUCPv 595) [36], which would give a total tooth count of about 80 teeth. Similarly, the dentary of the holotype of *Austalovenator wintonensis* has 18 alveoli [29,36], which suggests that the total number of teeth would have been at least 76.

**Table 1:** Tooth measurements of *Murusraptor barrosaensis*.

	Total length	Crown height	Root height	Crown base width*	Crown base length*
MCF-PVPH-411-39	47**	15**	32**	7.5	13.9
MCF-PVPH-411-41	--	15**	--	5	--
MCF-PVPH-411-42	50.3**	20.4**	29.9**	7.26	13.52
MCF-PVPH-411-43	28.22**	15	13.22**	5.5	9
MCF-PVPH-411-45	34**	14	20**	unprepared	11.5
MCF-PVPH-411-46	53.51**	49.79	3.48**	unprepared	14.8
MCF-PVPH-411-47 Premaxillary?	--	10.20	--	unprepared	6.2
MCF-PVPH-411-50	82.1	29	53.1	7.58	16.04
MCF-PVPH-411-51	—	21.08**	--	7.46	15
MCF-PVPH-411-52	51.15	14	37,15	5.06	9
MCF-PVPH-411-53	23**	15.5**		5	9.52
MCF-PVPH-411-54	--	12.3	--	3	5.8
MCF-PVPH-411-55	--	24**	--	~9.5	~13
MCF-PVPH-411-56	54.04**	17.46	36.58**	unprepared	13.4
MCF-PVPH-411-57	--	18	--	5	7.8
MCF-PVPH-411-64	54.01	20.05	33.96	8.12	15.7
MCF-PVPH-411-65	43**	15.5	27.5**	unprepared	10.2
MCF-PVPH-411-66	46.8	18.12	27.96**	7.48	9.2
MCF-PVPH-411-77 Premaxillary?	54.9	15.30	39.6	6.18	7.5
MCF-PVPH-411-78	60.6	23.01	37.59	unprepared	13.4
MCF-PVPH-411-79	55.28	19.04	35.88	unprepared	13.7
MCF-PVPH-411-81 Premaxillary?	--	14.32	--	4.8	7**
MCF-PVPH-411-85	23.32	13.02	10.3**	unprepared	11
MCF-PVPH-411-86	--	14.5	--	4	5.5
MCF-PVPH-411-87	--	12.28**	--	4.42	8**
MCF-PVPH-411-88	60	20.7		6.9	13.02

* = at the cervix dentis level (tooth neck);

** = preserved; measurements in mm.

**Fig 16:**
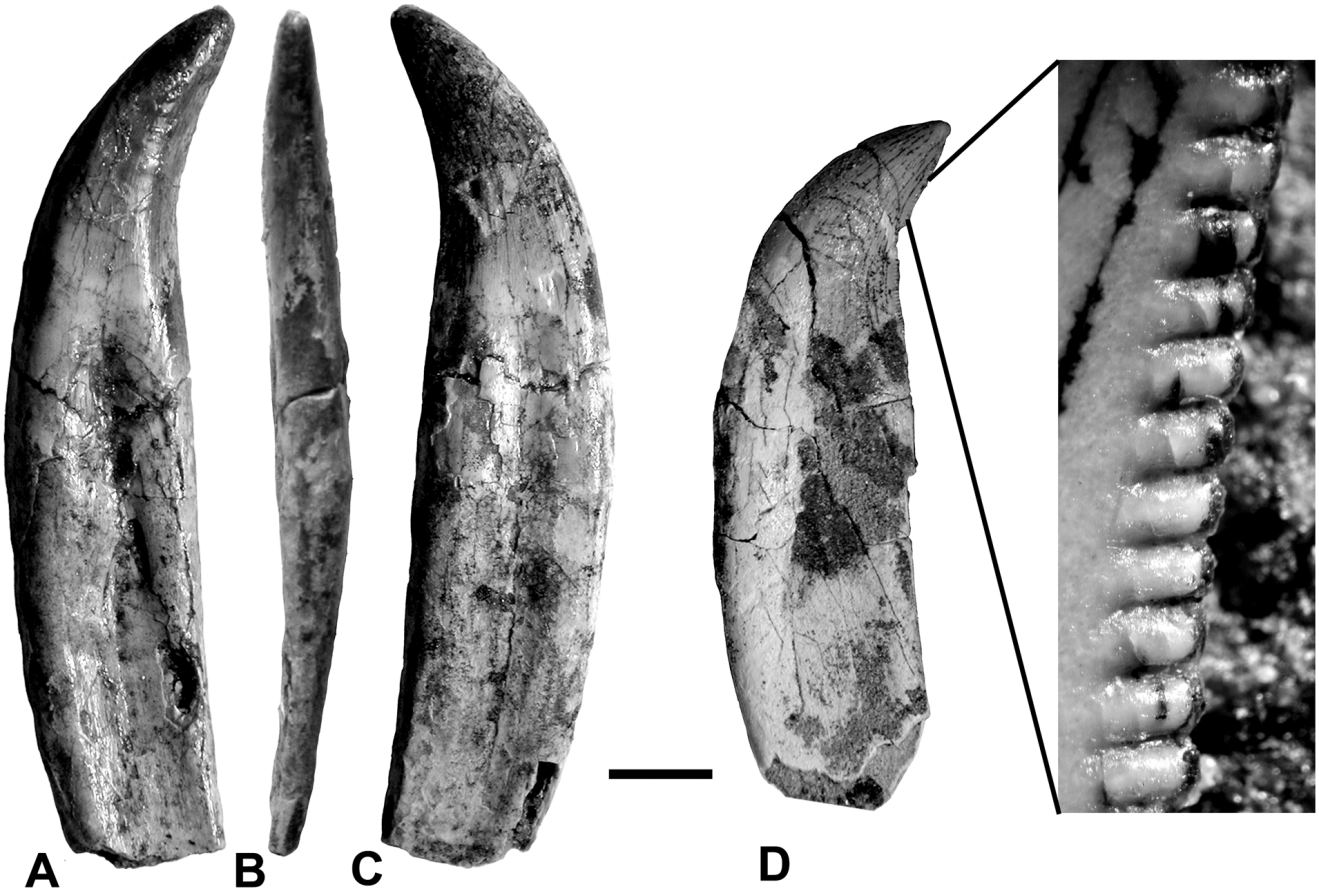
Maxillary teeth of *Murusraptor barrosaensis*, holotype, MCF-PVPH-411. Left? maxillary tooth MCF-PVPH-411-50 in mesial (A), posterior (B) and labial (C) views. Maxillary tooth MCF-PVPH-411-79 in mesial view (D) and close-up of posterior denticles. Scalebar: 1 cm.

For the estimated size of the animal (See S3 and S4 Files), these are all relatively small, the tallest one (MCF-PVPH-411.50) having a crown height of 29 mm (Fig 16A–16C).

The first teeth of megaraptorids that were unquestionably associated with skeletal material were from *Orkoraptor* [28], and these are comparable in size and morphology with those of *Murusraptor*. Teeth associated with the juvenile specimen of *Megaraptor* are also relatively small, with the maximum crown height of a maxillary tooth reported as 17 mm [35]. *Australovenator* teeth [29,36] are also similar in size (the largest crown height is 25 mm) and outline, although there are some differences in the denticulation. The relatively small ratio of the tooth to animal size can also be found in some spinosaurids and troodontids.

The largest tooth collected (MCF-PVPH-411.50) has a crown height 2.5 times the height of the smallest crown recovered (MCF-PVPH-411.54). Most collected teeth have roots, show the same morphology, and are preserved in the same way. Therefore it is assumed that all of them belong to the holotype specimen of *Murusraptor barrosaensis*.

The serrations are relatively small, and on first glance it appears that most of the teeth lack denticles on the anterior carina. This is also the case for *Megaraptor* [35] and *Orkoraptor* [28]. However, wherever the tip is preserved (MCF-PVPH-411.50/64/66/78/79/88), there are 6–10 serrations, usually completely worn down. The exceptions include MCF-PVPH-411.87 (which is probably a premaxillary tooth with a flat lingual surface, convex labial surface and large serrations on both sides of the tooth). The carinae are generally on the midline of the tooth, which makes it difficult to distinguish labial from lingual sides. There are exceptions, like MCF-PVPH-411.50 and MCF-PVPH-411.66, where the carinae extend far down the anterolingual margins of the teeth, although even in these teeth the denticles were restricted to the distal tips. This is even true for MCF-PVPH-411.77, which appears to be either a premaxillary or anterior dentary tooth (J-shaped in cross section). However, another premaxillary tooth with an almost identical crown (MCF-PVPH-411.52) has small serrations that extend far down the carina towards the root.

Sereno et al. [26] identified one tooth as *Aerosteon* because of its proximity to the rest of the associated skeleton. Although it falls within the size range of the cheek teeth of *Murusraptor*, this shed tooth is clearly from an abelisaurid [37].

#### Postcranium

*Axial Skeleton*. Twelve incomplete vertebrae were collected, and include dorsals, sacrals and proximal caudals. Most of these are neural arches only, which were not fused to the centra and disassociated easily after death. An isolated centrum and one particular neural arch probably belong together, even though they were found separated in the quarry by more than a meter.

There are six dorsals. Comparison with *Allosaurus* [47], *Piatnitzkysaurus* [3], *Sinraptor* [53] and *Megaraptor* [35] suggests that these are the 12th, 16th, 17th, 18th, 20th, and 21st presacrals. There is enough variability in theropods that some of these numbers may be off by one position, but the sequence is still probably correct.

**Fig 17:**
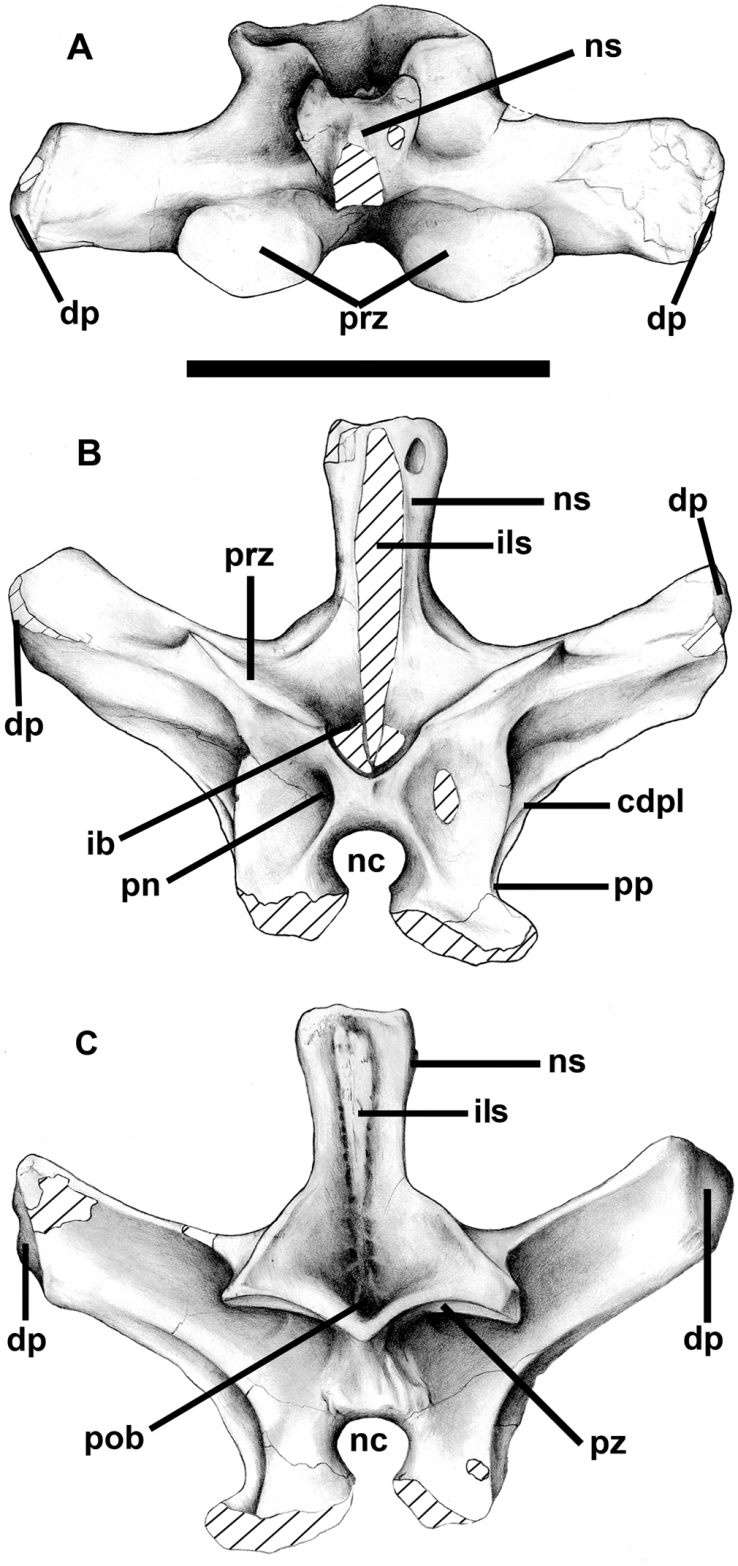
Twelfth presacral neural arch of *Murusraptor barrosaensis*, holotype, MCF-PVPH-411 in dorsal (A), anterior (B) and posterior (C) views. Abbreviations: cdpl, centrodiapophyseal lamina; dp, diapophysis; il, interspinus ligament scar; nc, neural canal; ns, neural spine; pob, postspinal basin; pn, pneumatopores; pp, parapophysis; prz, prezygapophisys; pz, postzygapophysis. Scale bar: 10 cm.

The twelfth presacral neural arch has a short, posterodistally curving neural spine. The spine is wider (32 mm) than anteroposteriorly long (23 mm), and the interspinous ligament scars extend almost the entire height on both anterior and posterior surfaces. The neural canal has a diameter of 22.7 mm. Anterolaterally the ventral margin of the neural arch forms the upper margin of the short-stalked parapophysis. The distance between the outer margins of the anterior zygapophyses is a third wider than the same measurement across the posterior zygapophyses. The kidney-shaped articular surfaces are 49 mm wide by 23.5 mm long, and the articular surface of each prezygapophysis inclines 35 degrees from horizontal (Fig 17B). The anterior zygapophyses are separated on the midline but there is no hypantrum. The prespinal basin has two large (15 mm high), triangular pneumatopores on either side of the anterior interspinous ligament scars. Below each prezygapophysis on the anterior face is another large, 15 mm high pneumatopore. Each prezygapophysis is on the anteromedial margin of the diapophysis.

A ridge extends posteroventrally to join the anterior centrodiapophyseal lamina, which splits near the convergence to form an elongate, slit-like fossa. Two infradiapophyseal laminae diverge ventrally, one converging with the posterodorsal corner of the centrum and the other with the parapophysis. A large infraprezygapophyseal fossa penetrates deep into the interior of the neural arch. The infradiapophyseal fossa also extends into the core of the bone with two pneumatic foramina on the right side, and one on the left. It is 77 mm across the posterior zygapophyses, and each articular facet is 34 mm across and 26 mm anteroposteriorly. The large disparity in width between the anterior and posterior zygapophyses is also present in the 12^th^ presacral of *Allosaurus* [47]. In contrast with the prezygapophysis, which has a flat surface, the ventral surface of the postzygapophysis is transversely concave in posterior view (Fig 17C). The medial margins of each facet meet on the midline and form a shelf that expands ventrally but does not develop into a distinct hyposphene. Above this shelf is a deep postspinal basin with two pneumatopores that invade the root of the neural spine. The interior of the neural arch is highly pneumatized, and at the top of the neural spine and at the ends of the diapophyses where the bone is thinnest, it collapsed to expose chambers.

**Fig 18:**
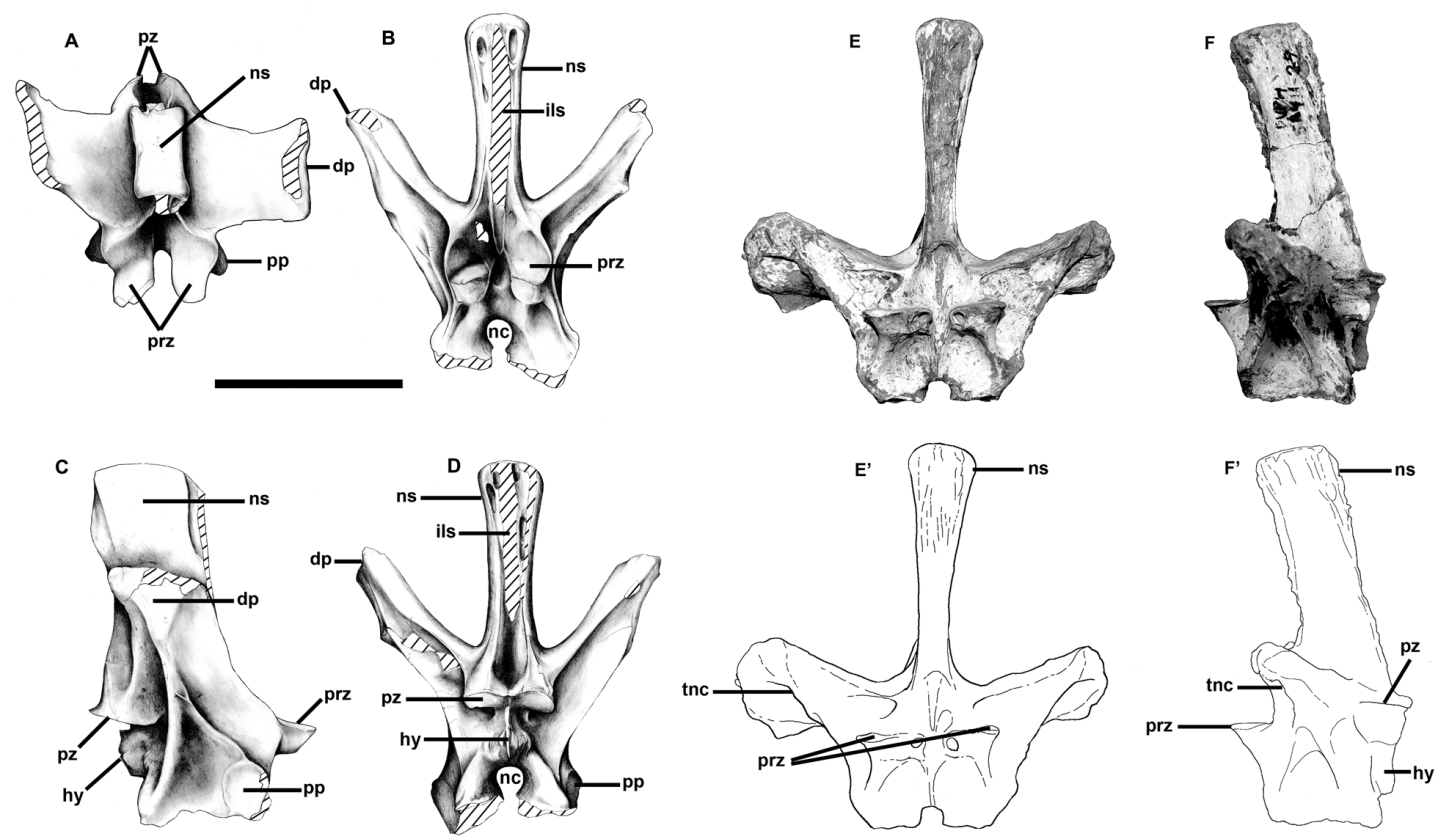
Neural arches of the sixteenth presacral and the third or fourth sacral vertebra of *Murusraptor barrosaensis*, holotype, MCF-PVPH-411. Sixteenth presacral vertebrae in dorsal (A), anterior (B), lateral (C) and posterior (D) views. Third or fourth sacral vertebra in (E-E’) anterior and (F-F’) lateral views. Abbreviations: dp, diapophysis; hy, hyposphene; ils, interspinous ligament scar; nc, neural canal; ns, neural spine; pp, parapophysis; prz; prezygapophyses; pz, postzygapophyes; tnc, tube-like neumatic canal. Scale bar: 10 cm.

The sixteenth presacral neural arch (Fig 18A–18D) has a 160 mm tall (from the top of the neural canal to the distal end), rectangular neural spine. The spine is anteroposteriorly longer (62 mm) than wide (29 mm), and distally the interspinous ligament scars extend anteriorly and posteriorly beyond the lateral surfaces of the spine. The width across the diapophyses would have been 160 mm, although each extends dorsolaterally 80 mm from the base of the neural spine. The neural canal has a diameter of 19 mm. The width is 53 mm across the anterior zygapophyses, and 49 mm across the posterior zygapophyses. Most of the parapophysis is on the neural arch, although the lower margin would have been on the centrum. The prezygapophyses are separated on the midline by a slot (Fig 18A), and the articular surfaces are continuous with anteromedially-facing hypantral articulations. The hyposphene is on a 22 mm high midline projection above the neural canal, and is separated from the postzygapophyses by a notch that is best seen in lateral view (Fig 18C). Pneumatopores invade the interior of the neural arch through the infraprezygapophysial fossa, both infradiapophysial fossae, and the infrapostzygapophysial fossa. Like most pneumatic structures, these are asymmetrical in distribution. For example there are four large (with diameters of up to 9 mm) pneumatopores in the left infrapostzygapophysial fossa, but only one on the right side. Two accessory pneumatopores invade the base of the left diapophysis, which has collapsed at the distal end to expose the pneumatic sinus inside.

The seventeenth presacral is essentially the same as the 16^th^, except that it is a little larger (neural spine height is 175 mm, the distance across the transverse processes is 180 mm), the parapophysis has moved entirely onto the neural arch, and the hypantrum is better defined. The prezygapophyses are separated on the midline, and each is supported by a pedicel with laminae extending ventromedially to the margin of the neural canal and ventrolaterally to intersect with the ventral end of the anterior infradiapophyseal lamina. The wall of the left prezygapophyseal pedicle is invaded by a sinus that is continuous with two small pleurocoels that extend into the body of the neural arch. Presumably this sinus is normally within the body of the pedicel and is not exposed externally, but in this case the wall was either resorbed or had collapsed post-mortem. The postzygapophyses are horizontal and form a continuous surface across the midline. In lateral view, they are divided from the hyposphene by a notch.

These trends continue onto the 18^th^ presacral, which has a 185 mm tall neural spine. The hypantrum is well-developed, but the prezygapophyses of this vertebra contact each other on the midline. The joint only functioned because there was a slot separating the hyposphene from postzygapophyses on the previous vertebra. The slot between the postzygapophyses and the hyposphene is closed by a strut of bone in the eighteenth vertebra. All of the pneumatic openings are confluent in the region between the roof of the neural canal and the base of the neural spine.

The twentieth presacral neural spine extends 200 mm above the neural canal. The lamina that connected the diapophysis and parapophysis, and divided the infradiapophysial fossa into two in more anterior vertebrae, has been reduced to a ridge. The postzygapophysis is a large triangular articulation (38 x 45 x 50 mm) that is continuous medially with the hyposphene.

Both the neural arch and centrum of the 21^st^ presacral were recovered. The entire vertebra is 360 mm tall, and its greatest width across the transverse processes is 210 mm. The centrum is 82 mm long, and the posterior intercentral articulation is 122 mm tall (bottom of neural canal to ventral surface of articulation) and 115 mm wide. The centrum has a large (20 mm long) pneumatic pleurocoel on the right side, separated by a posterodorsally oriented ridge from a much smaller posterior foramen, which is presumably for a blood vessel. Both of these features are also seen on the left side of the centrum, although the pneumatopore is somewhat smaller and the nutritive foramen is subdivided into 3 or 4 smaller foramina. The neural spine is thicker distally than any of the other preserved vertebrae, and is anteroposteriorly 78 mm long, and 55 mm wide across the top. The hypantrum is a well-defined triangular articulation on the medial side of the prezygapophysis. The margins of the prezygapophysial articulation are also well-defined as they are bordered by a low, sharp-edged ridge. Below the hyposphene three vertical ridges, which are poorly defined in more anterior vertebrae, form the margins for two depressions of unknown function. There is only a single infradiapophysial lamina on each side.

The sacrum is incomplete, but parts of three sacral vertebrae were collected. At least two of the vertebrae were coossified. Based on the positions of the transverse processes and sacral rib attachments, these are homologous with the first and second sacrals of *Allosaurus*. The neural arch of another sacral vertebra (Fig 18E and 18F) is more difficult to interpret, however. The anteriorly inclined transverse processes suggest it might be a caudosacral. The fact that the top of the transverse process apparently contacted the ilium directly is also consistent with it being the fifth sacral. However, there is a clear parapophysis anteroventral to each diapophysis and a long sloping contact with the sacral rib, which makes its identification as a caudosacral untenable; it is considered here as the third or fourth sacral neural arch. The dorsosacrals of *Allosaurus* on the other hand often have vestigial ribs below transverse processes that attach directly to the ilia [47].

The first true sacral centrum is well preserved, and is fused to both its neural arch and to the second sacral centrum. The sutures are still visible except where fusion between the sacral centra was complete ventrally. Unfortunately, most of the neural arch and second sacral were destroyed by erosion before the specimen was found. The anterior zygapophysis and hypantrum are similar to those of the 23^rd^ presacral. In contrast with *Allosaurus*, there is no parapophysis. The transverse process has become greatly expanded distally. Even though it is only partially preserved, the lateroventrally inclined sutural surface for the sacral rib is 71 mm high and 51 mm long. There is a large 27 x 14 mm pneumatopore in the infradiapophysial fossa. Another, slightly smaller pneumatopore penetrated the neural arch ventral to the prezygapophysial-diapophysial lamina. The internal structure of the neural arch, as exposed by erosion, has camellate pneumatic structure. The centrum is 110 mm long, and the anterior intervertebral articulation is 115 mm tall and 106 mm wide. The waist of the centrum is transversely only 51 mm below the pleurocoel. The pneumatopore is huge, being 35 mm long by 17 mm high on the right side, and slightly lower on the left side. The interior of the centrum is completely hollow (camerate) and it is possible to see right through the vertebra. The anterior and ventral walls of the centrum are less than 10 mm thick. Not much of the second sacral centrum is preserved. However, even though at least half of the lower margin of the bone is preserved, the internal structure is cancellous. This suggests that the second sacral centrum might not have been pneumatic, although it is more likely that this is just the bony support found at the end of all of the sacrals.

The third or fourth sacral neural arch was not fused with its centrum, nor with any of the sacral vertebrae (Fig 18E and 18F). However, the transverse process extends laterally, and slightly anterodorsally, 140 mm from the midline to directly contact the ilium. The distal end of the diapophysis expands to 74 mm, and the articulation for the ilium arches over a huge pneumatic fossa in the powerful lamina connecting the diapophysis and parapophysis. The suture for the sacral rib extends all the way from the parapophysis along this lamina, and encircles the pneumatic fossa. The tube-like pneumatic canal between the transverse process and the sacral rib has an anterodorsal-posteroventral diameter of 28.7 mm on the left side, and 19.2 mm on the right side. Because the height of the pneumatopore entering the ilium through the third sacral suture on the right ilium is larger (29 mm) than the one entering the fourth sacral suture (16 mm), it is possible that this is the third sacral neural arch rather than the fourth. However, the inclination and structure of the lamina between the parapophysis and diapophysis is more similar to that of the fourth sacral of *Allosaurus* [47]. It is therefore not possible to determine with certainty whether this is the third or fourth sacral neural arch. The dorsal surface at the end of the diapophysis is deeply pitted, presumably by pneumatic internal camerate structure. The transverse process is a tube that connected the sinuses in the interior of the neural arch to those of the ilium. Pneumatopores enter the neural arch anterodorsal to the left prezygapophysis, ventral to both prezygapophysial-diapophysial laminae, below both diapophyses, anteroventral to both postzygapophyses, and on either side at the base of the neural spine. The neural spine is the tallest (251 mm) of the vertebrae collected, and inclines somewhat anteriorly. The distal end is shorter (61 mm) and narrower (47 mm) than that of the 21^st^ presacral. Pneumatopores border the intervertebral ligament scars on both the front and back of the neural spine. The ligament scars extend all the way to the base of the neural spine in both the pre- and post-spinal fossae. The relatively shallow hypantrum is on the medial margin of a ridge that supports the prezygapophysis. Holes on the anterior edge of the hypantrum may be where extremely thin bone covering internal pneumatic sinuses collapsed. The hyposphene is continuous with the medial edge of the articular surface of the postzygapophysis. The ventral edge of the hyposphene is supported by a complex series of laminae. The largest is on the midline, and extends to the dorsal margin of the neural canal. This separates the two depressions that have been mentioned in connection with the 21^st^ vertebra, although the depressions are larger and deeper in the more posterior vertebra. Laminae separate this pair of depressions from a more ventrolateral pair, and another more dorsal pair. The prezygapophyseal articulations are horizontal, whereas the postzyapophseal surfaces are inclined at about 30 degrees above horizontal.

The neural spine of what was identified as the first caudal vertebra is high, and relatively thin (12 mm transversely through the base of the neural spine). Both anteriorly and posteriorly, the scarred bone for the interspinous ligaments extends to the top of the neural spine in lateral view. The transverse process is oriented mostly laterally, but inclines somewhat posterodorsally. If restored, the vertebra would be more than 290 mm across the diapophyses. The posterior zygapophysis in posterior view is inclined at an angle of about 45 degrees. The articulating surface, which is 40 mm tall by 26 mm long, is continuous ventromedially with the laterally facing hyposphene.

**Fig 19:**
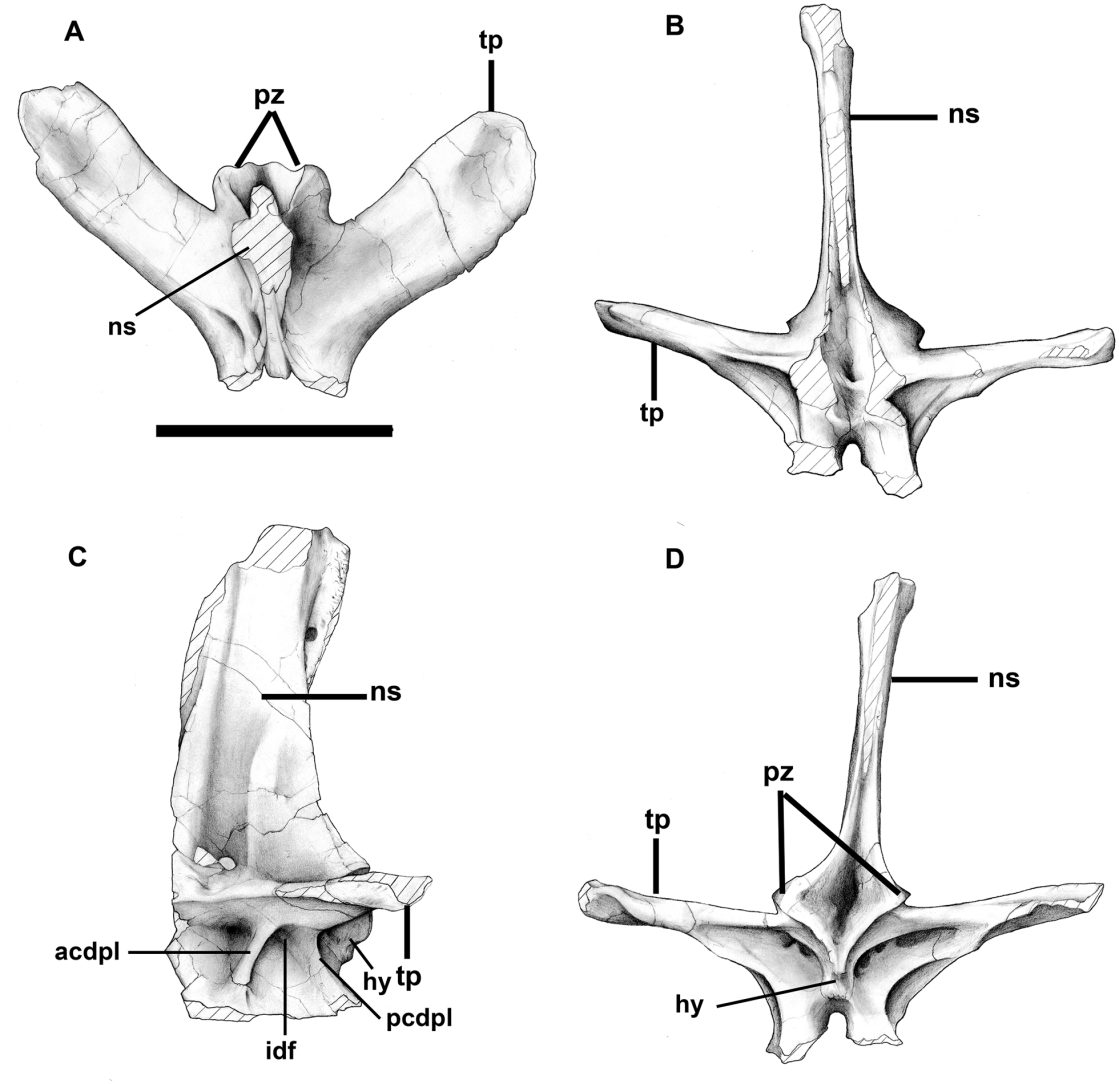
Anterior caudal neural arch of *Murusraptor barrosaensis*, holotype, MCF-PVPH-411in dorsal (A), anterior (B), lateral (C) and posterior (B) views. Abbreviations: acdpl, anterior centrodiapophyseal lamina; idf, infradiapophyseal fossa; ns, neural spine; pcdpl, posterior centrodiapophyseal lamina; pz, postzygapophyses; tp, transverse process. Scale bar: 10 cm.

A more complete neural arch (“Caudal A”) is from the anterior part of the tail and has a high neural spine that is tilted somewhat posteriorly (Fig 19). Each of the posterolaterally inclined, horizontal transverse processes is supported by a pair of ventromedially diverging centrodiapophysial laminae, anterior and posterior respectively. A large pneumatopore penetrates the medial wall of the infradiapophysial fossa. Deep fossae posteromedial to the posterior infradiapophysial laminae continue to the interior of the neural arch through two large pneumatopores on the left side, and four on the right (Fig 19D). Broad vertical grooves on the lateral surfaces of the neural spine terminate ventrally in 9.8 mm diameter pneumatopores (one per side) that communicate with the interior of the bone. The posterior zygapophyses are inclined at right angles to each other, and there is a well-developed hyposphene.

A more posterior but less complete neural arch (“Caudal B”) is essentially the same. The neural arch is lower, but the span of the transverse processes would have been about the same. Pneumatopores are not present on the lateral surface in the base of the neural spine, but do penetrate the walls of the three pneumatic fossae beneath the transverse process. Hyposphene and hypantrum are present.

As in *Murusraptor*, the neural spines of the two known anterior caudals in *Megaraptor* (MUC-PV 341) are rectangular and vertical, and thicken distally into lateral, knoblike processes.

There is an anterior cervical rib from the right side with a long, slender shaft. The capitulum is missing, but the tuberculum is similar to those of the cervicals of *Allosaurus*. The anterolateral process is also broken off, so its extent is unknown.

Ten dorsal ribs were recovered from the right side, plus two from the left side. All of these ribs include at least the heads, but isolated sections of shaft probably represent additional dorsal ribs.

**Fig 20:**
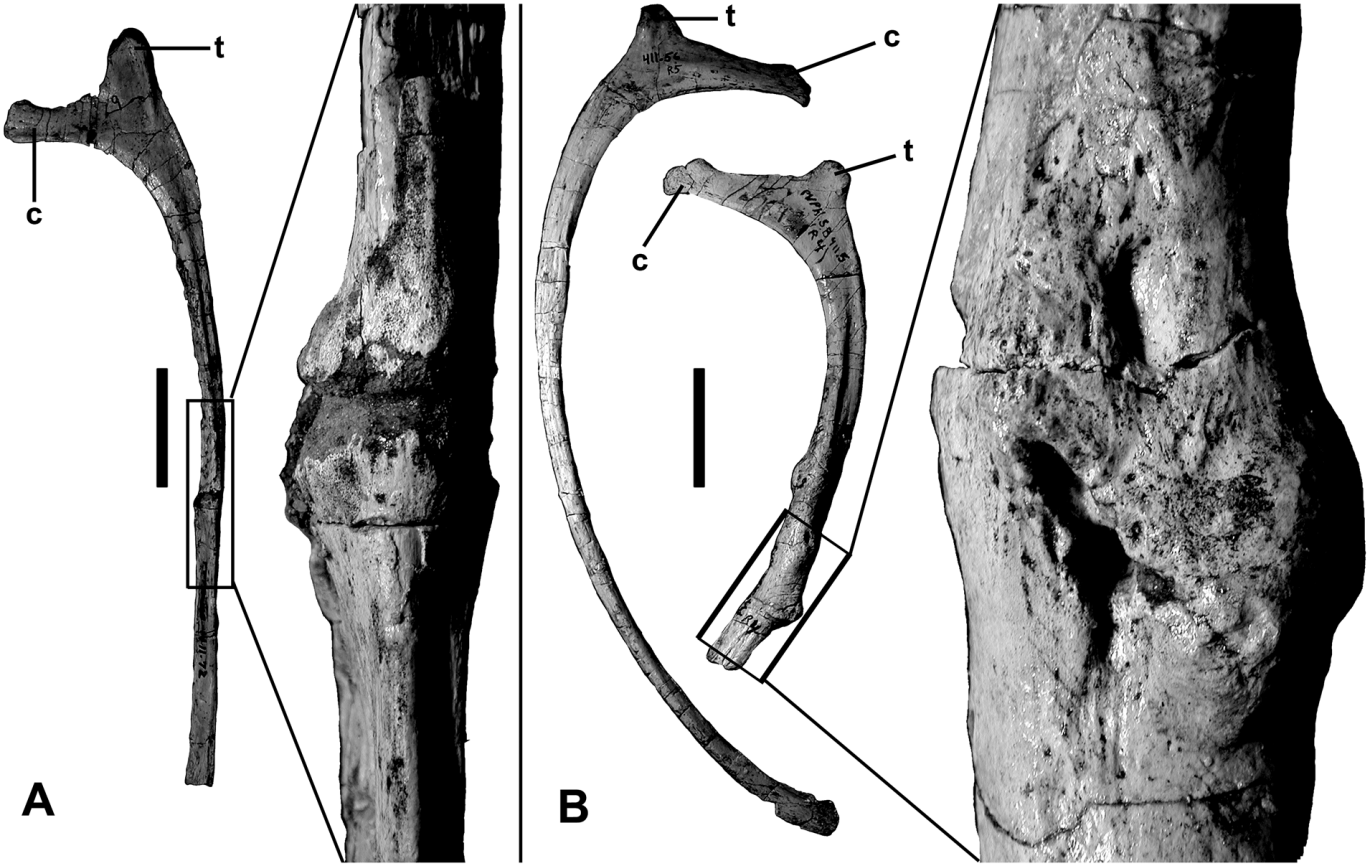
Presacral ribs of *Murusraptor barrosaensis*, holotype, MCF-PVPH-411 in anterior views. A) 11^th^ presacral rib with close-up of pathological area. B) 17^th^ presacral ribs with close-up of pathological area. Abbreviations: c, capitulum; t, tuberculum. Scale bar: 10 cm.

The presumed eleventh presacral rib (first dorsal rib) is long (640 mm on the outside curve, 631 mm when measured straight from the outside margin of the tuberculum to the distal end) and almost straight (Fig 20A). The shaft is relatively broad and flat (24 mm by 11 mm near the distal end).

The distal end expands somewhat and probably contacted a sternal element. There is a pathology 390 mm from the tuberculum and 230 mm from the distal end of the 11^th^ right rib. The bone is inflated on both sides of what appears to be an oblique pseudoarthrosis. The 15^th^ presacral rib from the right side has a bony callosity 195 mm from the tuberculum where the bone presumably broke and rehealed. On the 16^th^ right presacral rib, there is a callosity 330 mm from the tuberculum.

The 17^th^ presacral ribs are known from both sides of the body (Fig 20B). The left one is 710 mm long in straight-line measurement, and 820 mm measured along the outside curve. At midshaft, the rib measures 22.6 mm anteroposteriorly. The distal end expands to 35.5 mm but is thin (9 mm). It has an expansion 260 mm from the tuberculum that looks like a healed injury (perhaps a cracked rib). At the same distance from the head on the right rib, there is a severely pathological callosity that is deeply pitted. The pathological bone expands to a diameter of 40 mm around what appears to be an oblique fracture. The affected area continues for about 150 mm farther down the shaft where there is a second apparent break with a piece of bone extending anteriorly 14 mm beyond the anterior margin of the shaft. The shaft is misaligned and swollen in this area, and there is one large 9 mm broad pit probably for the escape of puss, which suggests it was infected.

At least four of the ribs were found in sequence. From the 18th presacral rib backwards, all dorsal ribs have round, ventrolaterally opening pneumatopores with diameters greater than 10 mm penetrating the posteromedial surface of the head below the tuberculum. Each of the 19^th^, 20^th^, 21^st^ and 22^nd^ presacral ribs also has a pneumatopore on the anterolateral side, almost directly opposite the medial pneumatopore. These pneumatopores open dorsomedially across the webbing between the capitulum and tuberculum.

The 18^th^ dorsal rib is from the left side and shows no sign of trauma. The 19^th^ rib from the right side has a small expansion, possibly representing a healed crack. The shaft of this rib is badly distorted, but it appears to be post-depositional damage. The 20^th^ rib on the right side has two pathological expansions in the shaft, one 150 mm from the tuberculum, and the smaller one 220 mm down the shaft. The 21st presacral ribs from both right and left sides taper consistently distally, and show no signs of pathologies. In cross-section, the rib shafts are T-shaped distal to the heads, and L-shaped farther down the shaft. The shaft of the 22^nd^ rib tapers ventromedially (like all of the other ribs), and ventroposteriorly, which gives the head a twisted appearance.

The 23^rd^ presacral rib lacks the capitulum. It has a relatively large and rugose tubercular articulation. The shaft curves strongly distal to the head and becomes relatively straight and even curves in the opposite direction distally. The distal end of the tapering shaft is only 6 mm in diameter. There are no pneumatopores in the depression posterodistal to the tuberculum as there are in all of the other posterior dorsal ribs. It seems that this rib, which would have been positioned medial to the front of the ilium, was not pneumatic.

The single chevron recovered with MCF-PVPH-411 is a simple blade-like structure 238 mm along its axis that curves down and backward. The absolute and relative size of the haemal canal is large (24.8 mm wide by 61.8 mm high), which indicates that it is from an anterior position in the tail. It is closed above the haemal canal. The laterally compressed haemal spine appears to have sloped posteroventrally at an angle of about 45 degrees from the axis of the tail. In lateral view, ventral to the haemal canal on the dorsoposterior surface of the haemal spine is a posteriorly projecting, tab-like process on the midline. The haemal spine is damaged distally, but would have been expanded at the end.

**Fig 21:**
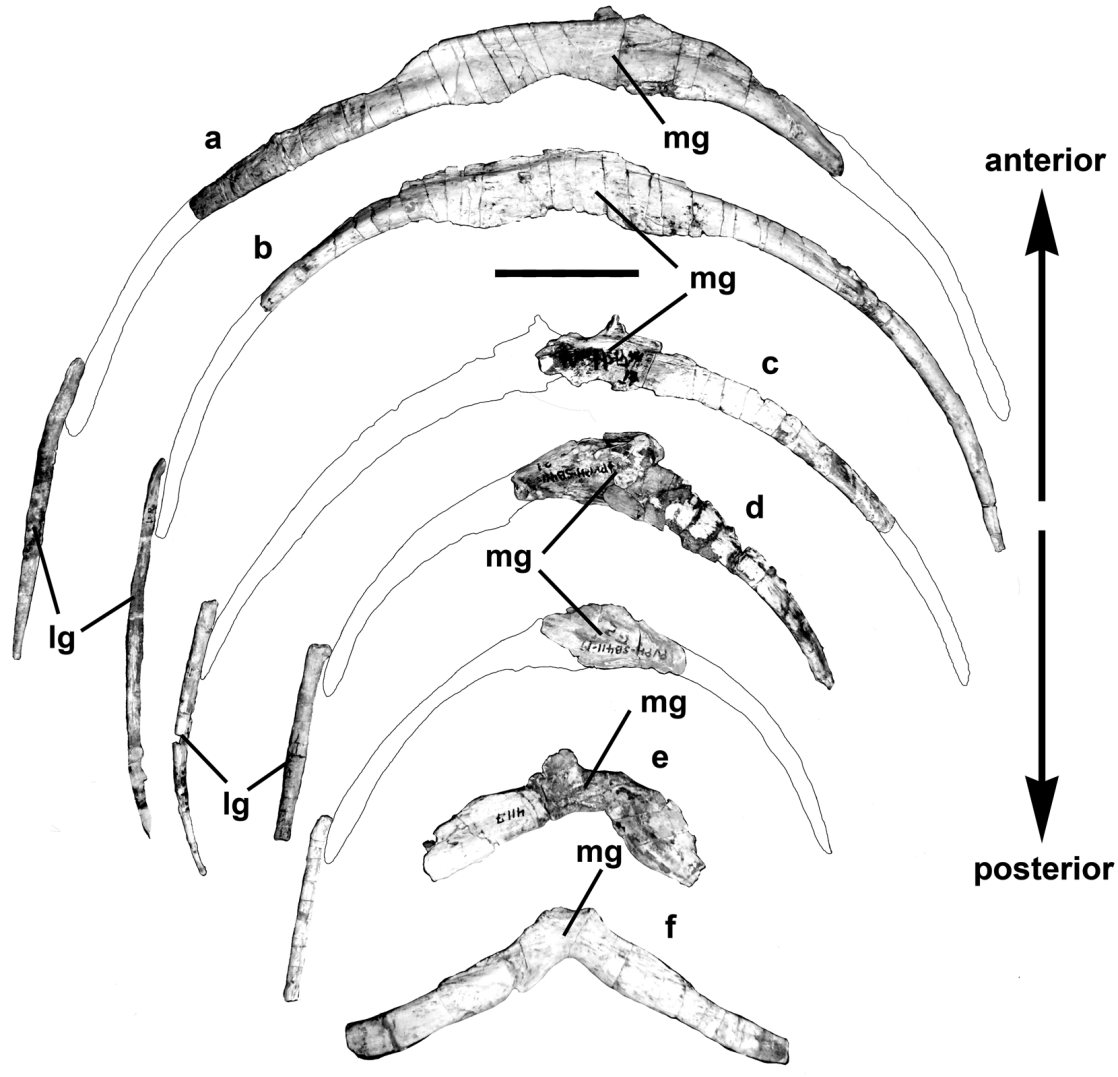
Gastralia of *Murusraptor barrosaensis*, holotype, MCF-PVPH-411 in ventral view. a-c, anteriormost elements; d-f, most posterior elements. Relative positions are tentative as well as reconstructed areas. Scale bar: 10 cm.

Medial and lateral gastralia were found mixed in with the rest of the skeleton (Fig 21). They include the medial heads of at least ten (six from the left side, four from the right) of the medial gastralia, three pairs of which are fused together into V-shaped median structures. Two of the fused pairs have the median gastralia meeting at relatively obtuse angles (about 150 degrees) and probably represent the most anterior sets [69]. However, the third better preserved fused set meets in a sharpen angle (around 120 degrees), which suggests it has a more posterior position in the gastralia basket. The tapering shafts of all of these sets are relatively thick, and are comparable in diameter with the dorsal ribs. Close to the midline, the gastralia have expanded (up to 45 mm anteroposteriorly), spoon-like ends for their complex crisscrossing contacts with the gastralia from the opposite side of the body. The widths of the gastralia show that the girth of the chest exceeded 700 mm. The shafts are grooved laterally for contact with the lateral gastralia. Although none of the lateral gastralia is complete, they are all much thinner and seem to have been shorter than the medial ones.

Fused medial gastralia are also reported in *Aerosteon* [26]. Unlike the condition in *Murusraptor*, the medial gastralia of *Aerosteon* are fused with the lateral gastralia, and are pneumatic.

#### Appendicular skeleton

**Fig 22:**
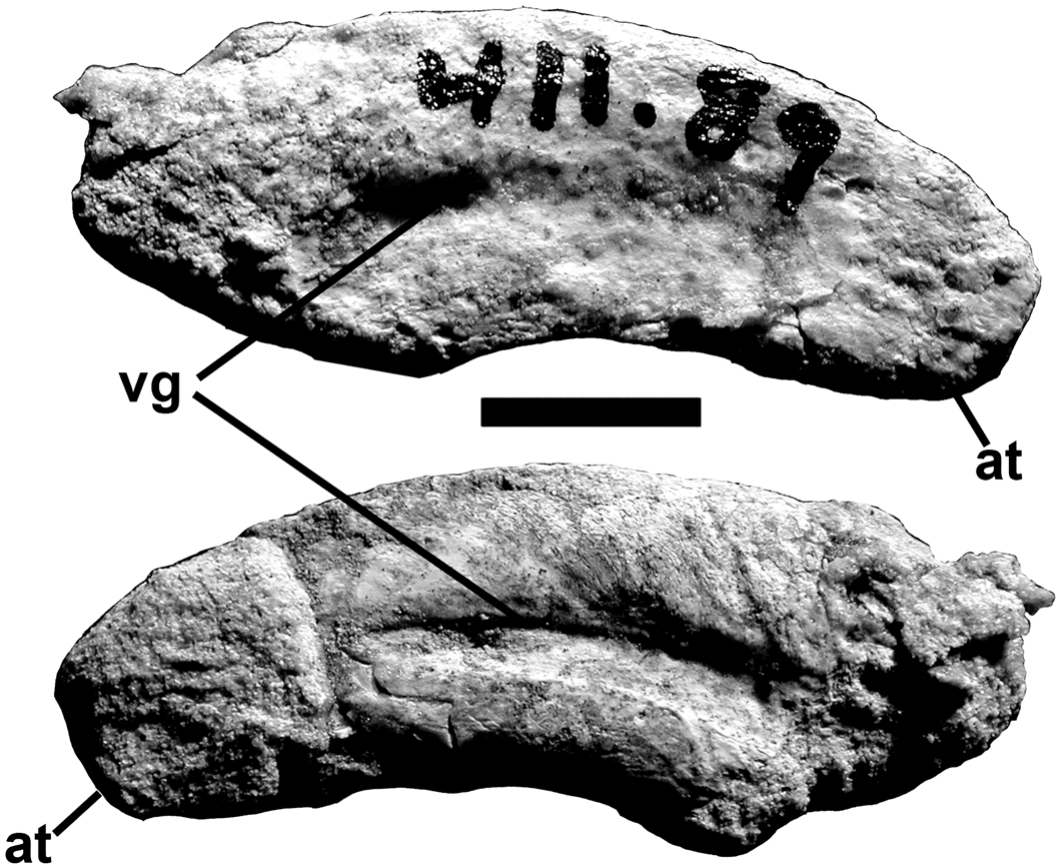
Manual ungual of *Murusraptor barrosaensis*, Holotype, MCF-PVPH-411 side views. Abbreviations: at, anterior tip; vg, vascular groove. Scale bar: 1 cm.

A single, poorly preserved ungual appears to be a small, laterally compressed manual ungual with sharp dorsal and ventral margins (Fig 22). At its thickest point it is 6.2 mm wide, and distally it narrows to 2 mm. There are no flexor or extensor tubercles, and the height of the proximal articulation is 17 mm high. The ungual is 42 mm long from the top of the proximal articulation to the distal end, although the tip is not preserved and the estimated length is probably close to 62 mm. Shallow vascular grooves along each side of the ungual are more or less symmetrical. The size is consistent with the third manual ungual phalanx of *Megaraptor* (MUC-PV 341 in [8]).

**Fig 23:**
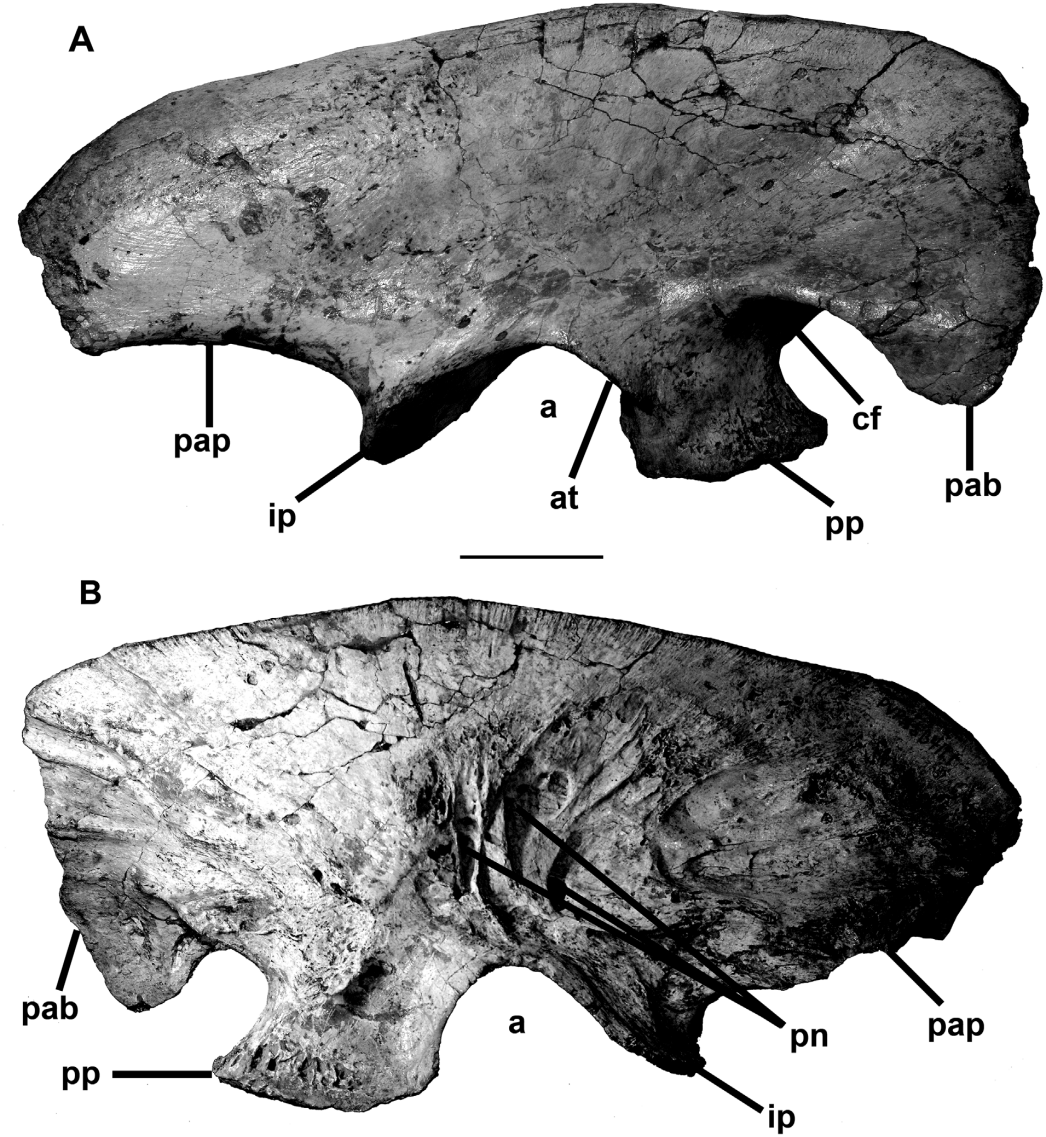
Ilium of *Murusraptor barrosaensis*, holotype, MCF-PVPH-411 in lateral (A) and medial (B) views. Abbreviations: a, acetabulum; at, antitrochanter shelf; cf, cuppedicus fossa; ip, ischial peduncle; pab, preacetabular blade; pap, postacetabular process; pn, pneumatopores; pp, pubic peduncle. Scale bar: 10 cm.

*Pelvic girdle*. The nearly complete, right ilium is 750 mm long (Fig 23). The left ilium is represented by most of the preacetabular blade. When viewed laterally, the entire dorsal margin of the ilium is shallowly convex (Fig 23A). It has a height (top of acetabulum to dorsal edge) to length ratio of 0.34, which is higher [70] than the same index in either *Ceratosaurus* (0.28) or *Torvosaurus* (0.29), but is similar to *Allosaurus* (0.34), *Giganotosaurus* (0.36), *Sinraptor* (0.36), and most other advanced carnosaurs of large size. It is slightly less than the height to length ratio (0.39) of the ilium in *Aerosteon* [26]. Overall length compared with maximum height (top of ilium to bottom of pubic peduncle) is similar to the majority of theropods, and falls on the regression line (S3 and S4 Files) for these comparative measurements in 78 theropod skeletons, including *Ceratosaurus* and *Torvosaurus*. The acetabulum is 20 cm long (anteroposteriorly) at its lateral margin. The anteroposteriorly elongate pubic peduncle is much deeper than the ischial peduncle. It is more vertical than in most carnosaurs, based on the angle between the anteroposterior axis of the ilium and the axis of the pubic peduncle (it is inclined 15 degrees anterior of perpendicular, compared with 36 degrees in *Allosaurus*, 22 degrees in *Mapusaurus*, 37 degrees in *Sinraptor* and 32 degrees in *Torvosaurus*). Corresponding with this reduced inclination of the pubic peduncle, the preacetabular notch is also wider than in other carnosaurs [70]. The pubic peduncle ends ventrally in a rugose, anteroposteriorly elongate sutural surface that is 160 mm long and less than half as broad. The proportions are the same in *Aerosteon*, although the length in that animal is 20 cm [26].

The lateral surface of the ilium is smooth, and in vertical section, is convex above and behind the acetabulum. Anterodorsal to the pubic peduncle and posterodorsal to the ventral, hook-like margin of the preacetabular blade, the lateral surface is concave. A distinct, tab-like antitrochanteric shelf overhangs the anterolateral margin of the acetabulum.

The preacetabular blade hooks ventrally, and somewhat laterally, anterior to the preacetabular notch, the ventral margin almost reaching the level of the pubic suture. The vertical anterior margin is shallowly scalloped. Each of the scallops represents the anterior margin of a ridge on the medial surface (Fig 23B). On both of the ilia, there are five of these ridges, the upper three of which are oriented roughly anteroposteriorly. The fourth ridge curves posteroventrally to become the wide cuppedicus shelf that is continuous with the anterior margin of the pubic peduncle. The dorsal surface of this ridge includes part of the suture for the transverse process of the first sacral vertebra. The rest of the suture extends onto the vertical medial surface of the preacetabular blade above this ridge. The fifth ridge is the most poorly defined and curves ventrally on the lateral wall of the cuppedicus fossa.

On the ventromedial edge of the postacetabular blade (Fig 23B), a ridge arises from the base of the ischial peduncle and extends posterodorsally to form the medial edge of a trough (about 8 cm across) for the caudofemoralis brevis musculature. In contrast with *Ceratosaurus* [71], the brevis shelf is relatively narrow as in *Allosaurus*, *Sinraptor* and other carnosaurs. The medial surface of the iliac blade has five sets of rugose sutures for the sacral ribs (Fig 23B), of which the second sacral (first true sacral) attachment extends well down the pubic peduncle and the fourth sacral attachment covers much of the medial surface of the base of the ischial peduncle. The attachment points for the first, third, fourth and fifth sacral transverse processes are also very conspicuous.

The ilium is unusual in that it is highly pneumatized. There are pneumatopores and pleurocoelic-like openings within the attachment points for the second and third sacral ribs and the third and fourth transverse processes. As pointed out in the section on vertebrae, these openings are continuous with canals that pass through the sacral ribs from the vertebrae. There are more than a dozen pneumatic openings on the medial surface of the ilium, the largest of which has a diameter of more than 2.5 cm. Similar to *Mapusaurus* [72], there are also what appear to be pneumatic openings within the brevis fossa. Unlike the condition in the carcharodontosaurids, however, the openings are at the back of the shelf. They pass forward from an oval sinus 15 cm long by 5 cm broad, breaking up anteriorly into a system of longitudinal canals separated by thin laminae of bone. It is possible that this sinus was completely enclosed within the ilium by a thin layer of bone that has collapsed. Broken and eroded surfaces on other parts of both ilia show the internal structure of the ilium is similarly composed of relatively large anastomizing canals and chambers that are remarkably different from the normal, non-pneumatic cancellous structure of other bones in this specimen. The frothy structure within the pubic peduncle and the preacetabular and postacetabular alae suggests a complex internal pneumatic system that would have made the ilium very light. No records of similar degree of pneumatization in the ilium have been described for any theropod species other than the related genus *Aerosteon* [26].

**Fig 24:**
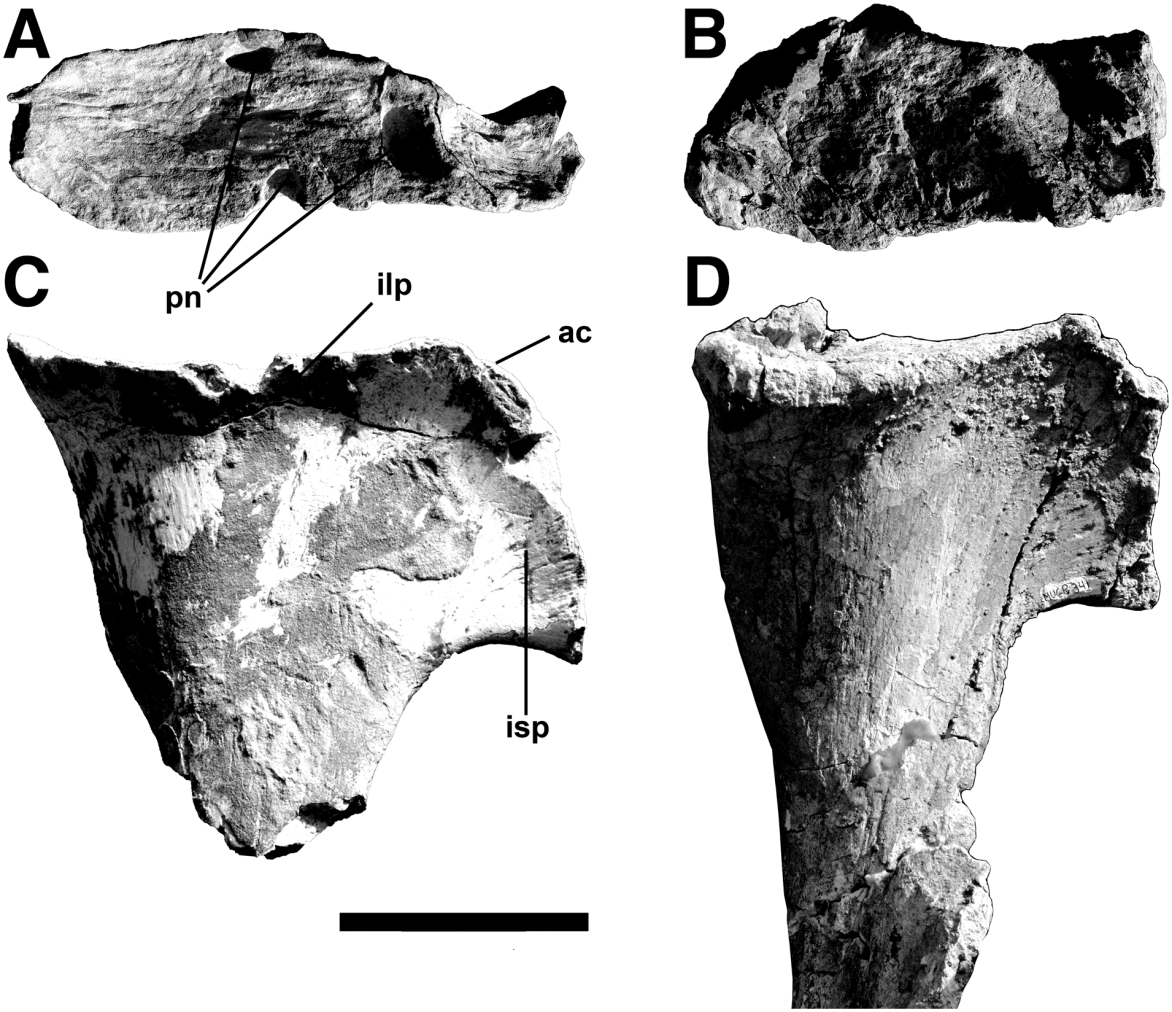
Pubis of *Murusraptor barrosaensis*, holotype, MCF-PVPH-411 (A, C) compared with the pubis of *Megaraptor namunhuaiquii* MUCPv 341 (B, D) in proximal (A, B) and lateral (C, D) views. Abbreviations: ac, acetabulum; ilp, iliac peduncle; isp, ischial peduncle; pn, pneumatopores. Scale bar: 10 cm.

Only the proximal portions of both pubes were recovered (Fig 24A–24C). However, the positions and orientations of the proximal regions were mirror-imaged when recovered from the rocks, showing that the distal ends were fused. As preserved, the shafts of the pubes were separated by 66 mm, and the ischial peduncles were separated by 108 mm. The iliac peduncle is large—the sutural surface for the ilium is almost 18 cm long with a maximum mediolateral width of 7 cm. The concave surface is heavily ridged, and seems to be penetrated by three pneumatopores with diameters of up to 1.5 cm. The distributions of the three openings are not symmetrical between the left and right pubes, and those on the left side appear to be associated with a large, smooth walled pit that might have been a pneumatic sinus in the suture between the ilium and pubis. If these are indeed pneumatic features, then they do not appear to have penetrated very deeply into the pubis because the eroded end of the pubic shaft is solid bone. The iliac suture is separated from the contact surface for the ischium by a relatively small acetabular concavity, which is 6.5 cm long (anteroventral to posterodorsal axis) laterally and 5.5 cm long medially. The ischial suture is almost perpendicular (92 degrees) to the iliac suture, and is 9 cm high by 4 cm wide. Even though the shaft of the pubis is only partially preserved, it was clearly oriented downwards from the longitudinal axis of the ilium. There is no distinct obturator foramen in the pubis.

**Fig 25:**
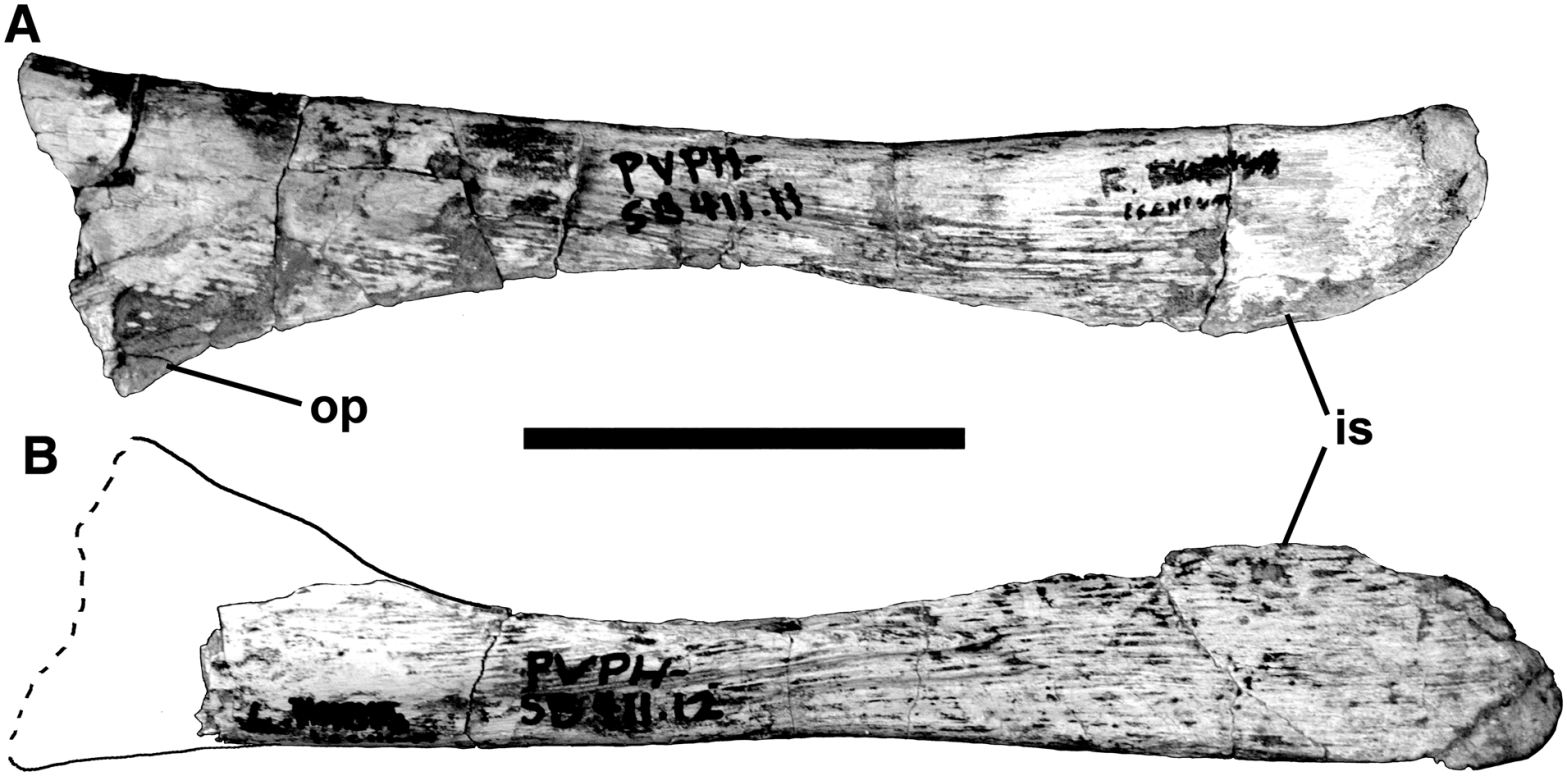
Ischia of *Murusraptor barrosaensis*, holotype, MCF-PVPH-411. Left ischium (A) and right ischium (B) in medial views. Abbreviations: op, obturator process; is, ischial symphysis. Scale bar: 10 cm.

Neither ischium is complete, although the base of the obturator process was recovered and there is no notch separating it from the distal part of the shaft (Fig 25). What is preserved suggests that the ischium was relatively short, that it contacted its neighbor distally, and that it only expanded slightly at the distal end.

**Fig 26:**
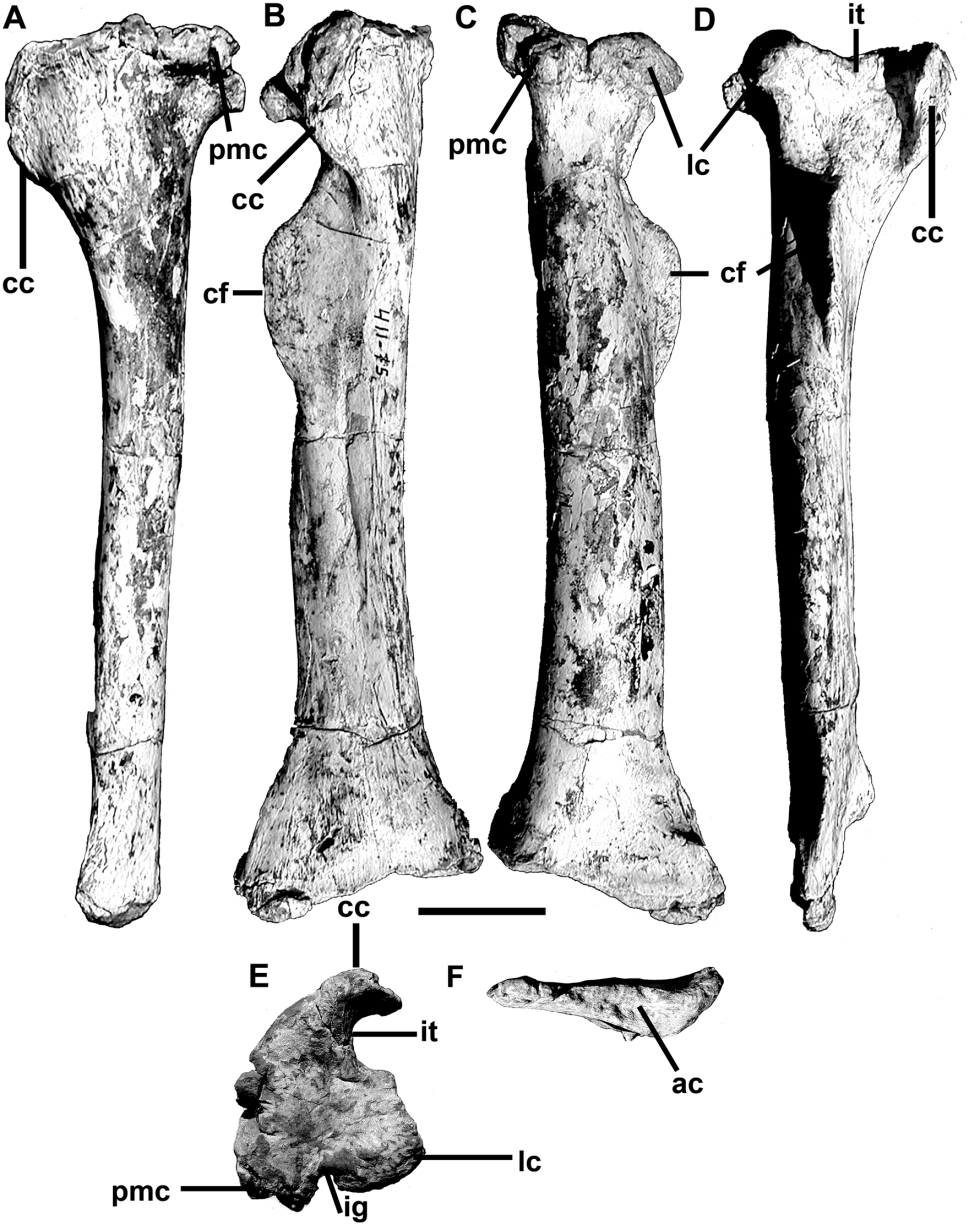
Tibia of *Murusraptor barrosaensis*, holotype, MCF-PVPH-411 in medial (A), anterior (B), posterior (C), lateral D), proximal (E) and distal views. Abbreviations: ac, astragalar contact; cc, cnemial crest; cf, crista fibularis; ig, intercondylar groove; it, incisura tibialis; lc, lateral condyle; pmc, posteromedial condyle. Scale bar: 10 cm.

A complete right tibia is beautifully preserved (Fig 26). It is 690 mm long, which is the size of a typical theropod in comparison with iliac length.

The proximal width of the tibia is 180 mm, the distal width is 195 mm, and the minimum shaft diameter is 58 mm anteroposteriorly (at that level, the shaft is 84 mm mediolaterally). The midlength circumference of the shaft is 232 mm, and the circumference to length ratio is 30, showing that the tibia is more gracile than those of *Ceratosaurus* [73], *Sinraptor* [53], and *Torvosaurus* [70], and falling within the range expected of *Allosaurus* [47]. The paired lateral and posteromedial articulations on the proximal end of the tibia are deeply separated by the posterior intercondylar groove as those of *Allosaurus* and *Torvosaurus*. The articular surface of the lateral (outer, fibular) condyle continues onto the lateral surface as a laterodorsally oriented articulation for the fibula. The fibular articulation extends anterolaterally onto a small triangular process that overhangs the incisura tibialis but does not form a continuous ridge with the crista fibularis. Between this process and the cnemial crest is a deep, smooth surfaced incisura tibialis that is anteroposteriorly shorter than the lateral condyle. The cnemial crest is powerfully developed and the anterior edge extends laterally as a strong vertical ridge, but as in *Allosaurus* it is noticeably less pronounced than those of abelisaurids [7,74] and *Ceratosaurus* [64]. Two vertical grooves on the anterior edge of the cnemial crest are presumably for attachment of the femorotibialis muscle. Distal to the head, but clearly separated from it, there is a pronounced ridge (crista fibularis) on the lateral margin of the extensor surface for attachment of the proximal interosseum tibiofibulare ligaments and contact with the fibula. This ridge is strong in abelisaurids, allosaurids, carcharodontosaurids, coelurosaurs and *Piatnitzkysaurus*, but is not as pronounced in *Ceratosaurus* and *Dilophosaurus*. As in most theropods, there is a foramen in the shaft posteromedial to the fibular crest. The anterior surface of the shaft is flattened, whereas the other surfaces are evenly rounded in cross-section. A ridge 150 mm from the distal end of the bone marks the dorsal limit of the ascending process of the astragalus. This suggests the ascending process was about 22% the length of the tibia compared with 20% in allosaurids and up to 33% in tyrannosaurids. The distal end expands medially and especially laterally from the shaft of the tibia, and the anterior surface is flat for the astragalus, calcaneum and distal end of the fibula. On the posterior surface, there is a flat distolateral extension that forms the distolateral margin of a groove that wraps around it onto the thin, lateral edge. The distal end slopes distolaterally at an angle of about 15 degrees.

**Fig 27:**
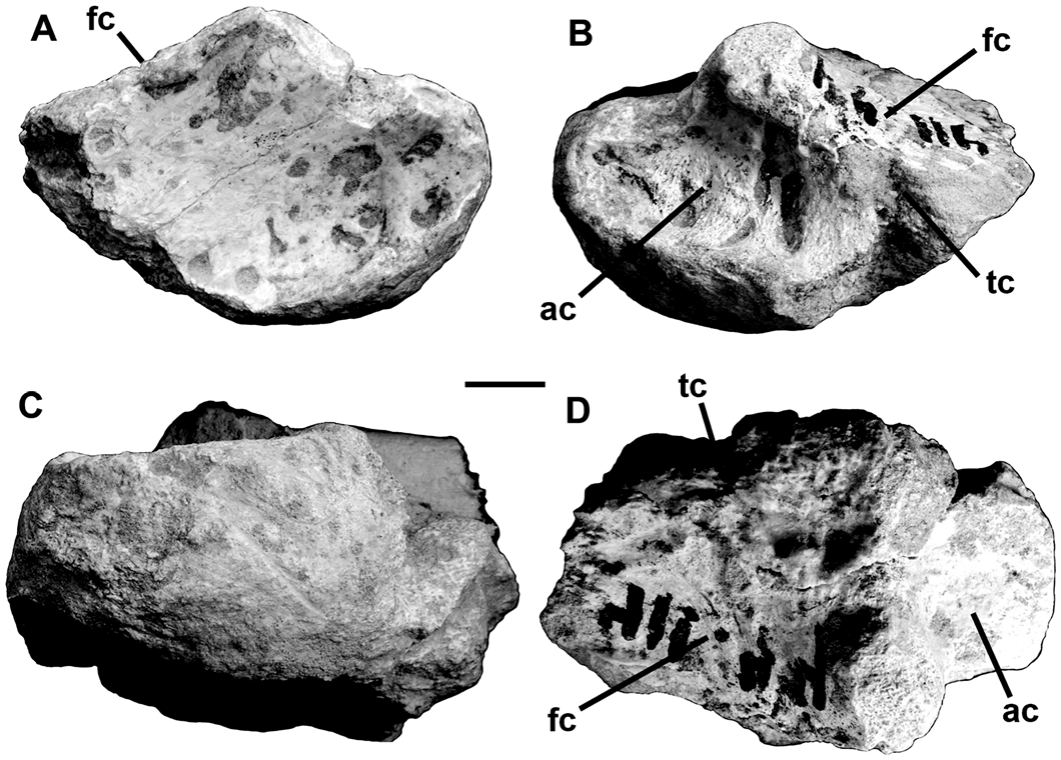
Calcaneum of *Murusraptor barrosaensis*, holotype, MCF-PVPH-411 in lateral (A), medial (B), distal (C) and proximal (D) views. Abbreviations: ac, astragalar contact; fc, fibular contact; tc, tibial contact. Scale bar: 1 cm.

A calcaneum was recovered from the quarry near the distal end of the right tibia (Fig 27).

The ventral edge of the calcaneum was damaged before burial but most of the bone was positioned anterior to the tibia. The contact surface for the tibia is rather rough, indicating that there was no movement between the two bones. The dorsal surface is excavated for a smoothly concave articulation for the fibula, however. The medial surface is heavily sculptured, and as in allosaurids and sinraptorids [53] there is a peg-like process posteromedial to the fibular articulation that would have plugged into a socket in the astragalus. The lateral surface is concave as in most theropods, but there is a lip of bone supporting the lateral edge of the tarsometatarsal articulation. Although the astragalus is not present, its width can be measured as 175 mm when the tibia and calcaneum are in articulation. The maximum width of the calcaneum is 28 mm, and therefore is only 16% the width of the astragalus. It is a considerably thinner element than the calcanea of allosauroids [53], and compares better with coelurosaurs.

## Discussion

### Comparison with *Megaraptor*

The three known associated skeletons of *Megaraptor* are from the older Portezuelo Formation, which is separated from the Sierra Barrosa Formation by Los Bastos Formation [41]. The holotype of *Megaraptor namunhuaiquii* (MCF-PVPH-079) was collected from Sierra del Portezuelo [27], which is 50 km to the west of where the skeleton of MCF-PVPH-411 was collected.

There is no overlap of elements between the fossils that were described as the holotype of *Megaraptor namunhuaiquii* [27] and *Murusraptor barrosaensis* (MCF-PVPH-411). However, in addition to the holotype material described, there are a number of unprepared and undescribed bones (MCF-PVPH-80) that were collected in association with MCF-PVPH-079, including four caudal vertebrae, two ribs (one almost complete), and gastralia. This cluster of fossils was found far enough from the material described as the holotype of *Megaraptor namunhuaiquii* that the collectors could not be sure whether or not they belonged to the same specimen (Novas, personal communication 2004). One almost complete dorsal rib would have been found associated with MCF-PVPH 79 (Novas, personal communication 2006). The centra of the caudal vertebrae are about 9.5 cm long, which is about the same size as the sacral vertebra of MCF-PVPH-411. Furthermore, the undescribed centra have large pleurocoels, just like those of another *Megaraptor* specimen (MUCPv 341, [8]. However, the neural arches have not been well enough prepared for comparison with MCF-PVPH-411.

The centra of the anterior pair of caudal vertebrae of MCF-PVPH-079 are each about 10 cm long, which is about the same size as the first sacral centrum of MCF-PVPH-411. Comparing centrum length between the first sacral of MCF-PVPH-411 and the undamaged right side of the first caudal of MCF-PVPH-079 suggests that the two animals are about the same size. However, comparison of the anterior centrum width and anterior centrum height of the same two specimens produces dimensions that are about 30% smaller, which suggests that MCF-PVPH-411 is a more lightly built animal than MCF-PVPH-079. Whereas the centrum of MCF-PVPH-411 is narrowly waisted (maximum central width at midlength is 52 mm), the midlength width of the first caudal of MCF-PVPH-079 is much thicker (91 mm). The neurocentral sutures of the MCF-PVPH-079 are fused, which indicates that the holotype was a more mature animal, in keeping with the fact that the size differences suggest it was a larger animal. Although these measurements compare vertebrae that are separated by four positions in the vertebral column, they do suggest that the holotype of *Megaraptor namunhuaiquii* was a slightly longer, more massive and more mature animal. Regardless of slight differences in size and robustness, there are some fundamental similarities in the two specimens that are only rarely found in other theropods. Like the first sacral vertebra of MCF-PVPH-411, the undescribed centra of MCF-PVPH-079 have large pleurocoels (as they do in the second *Megaraptor* specimen described by Calvo et al., [8]. The neural arches of MCF-PVPH-079 are neither well-preserved nor well-prepared, but again show the same fundamental features as MCF-PVPH-411. The internal cancellous structure is widely open and suggestive of its pneumatic nature. The anterolateral surface of the base of the transverse process is concave ventral to the base of the prezygapophysis. On the right side of MCF-PVPH-079, the concavity is pierced by three pneumatopores, the largest of which has a diameter of 11 mm. The transverse processes of MCF-PVPH-079, like those of MCF-PVPH-411, extend as much posteriorly as they do laterally, so that the distal ends would have terminated at a level well behind the back of the postzygapophyses and centrum. The neural spines are long anteroposteriorly, flat lateromedially, and are deeply excavated on the midline both anteriorly and posteriorly. The other two caudals of MCF-PVPH-079 are similar in length to the first two caudals, but the centra are lower and narrower, which suggests that they were separated in the living animal by several vertebral positions.

Two left ribs with heads of MCF-PVPH-079 are similar in morphology and size to the anterior dorsal ribs of MCF-PVPH-411. The larger of the two is 90 cm long on the outside curve (between tuberculum and distal end), compared with an 82 cm length of the fifth dorsal rib of MCF-PVPH-411. The rib is broken in two places, and one of the two fractures appears to have been partly healed before death, whereas the other break seems to be post-mortem. The distance between the tuberculum and capitulum is 165 mm in MCF-PVPH-079, and 160 mm in MCF-PVPH-411. The tuberculum is offset posteriorly in both specimens, whereas the capitulum widens near the articular end. In both cases the shafts curve continuously throughout their length, but flatten out distally (the cross-section of MCF-PVPH-079 is 15 x 35 mm, whereas that of MCF-PVPH-411 is 10 x 33 mm). The second rib with a head from MCF-PVPH-079 is incomplete ventrally, but the head and proximal shaft are comparable with the eighth dorsal rib of MCF-PVPH-411. The maximum distance between the edges of the tuberculum and capitulum is 175 mm in MCF-PVPH-079, and 160 mm in MCF-PVPH-411.

Several nearly complete gastralia are preserved in MCF-PVPH-079, in addition to numerous fragments. The most complete right medial gastralium has a straight-line, lateromedial width of 51 cm, but is 56 cm when measured on the outside curve. Taking into account the curvature of the gastralium and the position of the midline of the body, the minimum width of the chest can be estimated as 80 cm. Lateral to the ventromedial, tapering section near the midline, the gastralium expands by means of thin anterior and posterior flanges to an anteroposterior length of 4 cm. This is precisely the situation seen in MCF-PVPH-411, although there is clearly fusion between right and left medial gastralia and the anteroposterior length of the expansion is somewhat greater (up to 44 mm in an anterior medial pair of gastralia). These and other clues suggest that the medial gastralium of MCF-PVPH-079 was from a more posterior position than those of MCF-PVPH-411. A pair of fused gastralia of MCF-PVPH-079 seem to represent an even more posterior position in the abdomen. It includes a complete, 22 cm long lateral gastralium that contacts 17.5 cm of the distal tapering end of the medial gastralium, which is incomplete proximally (medially). The distal (dorsolateral) end of the lateral gastralium is T-shaped in cross section where it would have overlapped the ventral end of the dorsal rib in life. The two gastralia are not fused for most of the overlapping region, but are indistinguishably fused at the proximal (ventromedial) end of the lateral gastralium. The gastralia are similar to those of MCF-PVPH-411, and although many features are still covered by matrix, the shape of the dorsal rib head is almost identical to the fifth dorsal rib of MCF-PVPH-411.

A medial fragment of the distal end of the right tibia of suggests that the tibia of *Megaraptor* has the same morphology as that of MCF-PVPH-411. The distal surface of the tibia of MCF-PVPH-079 is deeply furrowed, suggesting that it is from a more mature individual than MCF-PVPH-411 in which the distal surface is smooth. An anteroposterior measurement of the distal end of MCF-PVPH-411 is 72% the same dimension in the holotype.

A second partial skeleton of *Megaraptor* (MUCPv 341) was collected on the north shore of Los Barreales Lake [8,75], which is 45 km north of where the skeleton of *Murusraptor barrosaensis* was collected. There is considerable overlap of elements in the two *Megaraptor* skeletons (MCF-PVPH 79, MUCPv 341), although the Barreales specimen is 92% the size of the holotype (based on five measurements of the ulna, manual phalanx I-1 and manual ungual I-2).

MUCPv 341 includes a cervical vertebra, several caudal vertebrae, dorsal ribs, two haemal arches, and a pubis that can be compared with MCF-PVPH-411. The neural arch of the cervical vertebra of MUCPv 341 can only be compared indirectly with that of the anterior dorsal of MCF-PVPH-411. In the presacral column, these two vertebrae were separated by at least three positions and it is not surprising that there are many significant differences. The diapopostzygaphophyseal lamina is well developed in both specimens, but MUCPv 341 is different in that a pair of pneumatopores are present in each side of the neural arch [8]. The prezygapophyses are well-separated in both specimens, and the articular surfaces are mounted on short pedicels. The distal ends of the diapophyses are triangular in section, and are supported by laminae that bound depressions with asymmetrically distributed pneumatopores. Not surprisingly, the epipophysis of the cervical of MUCPv 341 is more pronounced than that of the anterior dorsal of MCF-PVPH-411. There is no ridge connecting the epipophysis with the prezygapophysis in the dorsal vertebra. Both specimens have strong interspinous ligament scars along the entire heights of both the fronts and backs of the neural spines. As pointed out by Calvo et al. [8], this is different from the conditions in many other theropods where the interspinous ligament scars are more strongly developed distally. The cervical of MUCPv 341 is relatively smaller than the anterior dorsal of MCF-PVPH-411.

The two caudal vertebrae of MUCPv 341 are pathologic in that they are fused to each other and to the haemal arch between them. Together the fused centra are 152 mm long. Calvo et al. [75] postulated that they were proximal to caudal 8 in position. However, based on the size of the transverse processes compared with the transition point in other theropods, they could be as far back as the twelfth. There are no caudal centra associated with MCF-PVPH-411, but three neural spines of anterior caudals can be compared with MUCPv 341. The neural spine heights of the fused caudals of MUCPv 341 are no more than 154 mm (from the top of the canal to the top of the spine), which lower than any of the three preserved spines (213, 183, 156 mm respectively) of *Murusraptor*. The fact that morphologically the closest of the three to the fused two caudals of MUCPv 341 appears to be the mid-sized one suggests that the Barrosa theropod was larger than the Barreales *Megaraptor*. Morphologically, the caudal neural arches of both animals are very similar. The neural spines of MUCPv 341 are slightly taller than their corresponding centra. Although there are no associated centra in MCF-PVPH-411, the caudal neural spines are all taller than the sacral centrum, which is similar to MUCPv 341 in which the caudal neural arches are taller than the centra. The presence of pneumatopores in the sacral centrum of *Murusraptor* (MCF-PVPH-411) suggests that pneumatopores might have been present in the caudal vertebrae of the same specimen, just as they are in MUCPv 341 and the holotype of *Megaraptor namunhuaiquii*. The scarring for intraspinous ligaments is well developed on the front and back margins of the neural spines in both specimens. The elongate transverse processes of MUCPv 341 and the more posterior two caudal vertebrae of MCF-PVPH-411 are plate-like and horizontal, and are supported ventrally by a pair of converging buttresses that bound pneumatic recesses and/or pneumatopores. This is a particularly strong indication that these animals are closely related. As pointed out by Calvo et al. [8], *Torvosaurus* may be the only other theropod with similar buttressing.

The haemal arches of both specimens are quite similar in shape, size and inclination (as determined by the proximal intervertebral articulation and the length of the trough extending down the back of the spine from the haemal canal). One haemal arch of MUCPv 341 is fused to the coossified caudal centra, and presumably therefore preserves its natural position. This spine has an axial length of 185 mm, although the distal end extends only 140 mm below the level of the ventral surface of the vertebrae. A second, unattached haemal arch is 151 mm long, and is clearly from further back in the tail. Only one haemal arch was recovered with MCF-PVPH-411, and it is taller (238 mm) than either of the two chevrons of MUCPv 341. Both the absolute and relative size of the haemal canal is larger in *Murusraptor*, showing that it is from a position more anterior in the tail. Nevertheless, the haemal arches of both specimens are fundamentally the same posteriorly curving, bladelike structures with dorsally closed haemal arches. Distal to the haemal canal on the dorsoposterior surface of the haemal spine, there is no process in MUCPv 341. This slight difference might just have something to do with the chevrons of this specimen being more posterior in the tail.

The single ungual recovered of *Murusraptor* is slightly smaller than the third ungual of the hand of MUCPv 341, even though the former specimen appears to be larger than the latter. It lacks a distinct flexor tubercle, which could represent a real anatomical difference between the two specimens.

Only the proximal ends of both pubes were recovered with *Murusraptor*, whereas most of the right pubis is known for MUCPv 341 [8] (Fig 24B–24D). In both specimens the iliac suture is anteroposteriorly elongate, and the acetabular region of the bone is reduced. The ischial suture is high but narrow in *Murusraptor*, as it is in MUCPv 341, which Calvo et al. [8] found comparable with basal tetanurans like *Torvosaurus* and *Piatnitzkysaurus*. The overall length of the proximal head of the pubis is bigger in *Murusraptor*, but is more robust in the Barreales *Megaraptor*. The differences do not seem to be related to postmortem distortion, and are accentuated by morphological differences. The anterior edge of the pubis just below the iliac suture is more sharply defined in MCF-PVPH-411 because the lateral (and to a lesser extent also the medial) surface(s) posterior to the edge are concave, whereas they are convex in MUCPv 341. The iliac sutural surface is flatter dorsally in MUCPv 341, lacks pneumatopores and is inclined to face dorsomedially rather than dorsally. In both specimens, the pubis is advanced in that the obturator foramen has been opened up, which is not true in basal tetanurans like *Torvosaurus* and *Piatnitzkysaurus*. The iliac and ischial sutural surfaces are almost perpendicular to each other in both specimens, which is something that is not common in other theropods.

Of the several types of theropod teeth found at Los Barreales [76–77], all have been difficult to associate with the *Megaraptor* skeletal material because of the nature of bonebeds [8]. Nevertheless, some of the teeth from Los Barreales are very similar to those associated with *Murusraptor* and can be assigned with confidence to Megaraptora. MUCPv 723, for example is a rooted tooth with a total height of 49 mm, a Fore-Aft Base Length of 13 mm, and sixteen denticles per five millimetres. There are only five serrations at the tip of the anterior carina. The shapes of the tooth and its denticles, the positions of the carinae, and the measurements all fall within the range of the teeth found with the Barreales theropod.

Recently, a juvenile specimen of *Megaraptor namunhuaquii* (MUCPv 595) was communicated with skull and postcranials elements preserved [35]. The specimen includes a partial skull and an articulated vertebral sequence of nine cervicals (including the axis) and twelve dorsals. The circular foramen magnum of MUCPv 595 is rimmed by basioccipital, exoccipitals and supraoccipital as in *Murusraptor*. Both specimens show a quadrangular frontal, although MUCPv 595 seens to be slightly narrower posteriorly. The exoccipital-opisthotic complex is separated from each other by the supraoccipital in both forms as well as the presence of a deep excavation of the dorsal surface of the occipital condylar neck and a subcondylar recess on its ventral surface. MUCPv 595 and *Murusraptor* show basal tubera separated by a wider space than the diameter of the occipital condyle. Although postranial elements are briefly described in that contribution, some comparisons can be done upon the figured bones (Figure 8 in [35]). All the available vertebrae of *Murusraptor* show, despite the size diference and possible a more mature ontogenic stage, a lighter arquitecture on the neural arch laminae. In general, the neural arches of *Murusraptor* seem to be relatively shorter anteroposteriorly and taller dorsoventrally than those from MUCPv 595, likely due the different ontogenic stages of each specimen. In lateral view, the centrodiapophyseal laminae of *Murusraptor* are forked in anterior and posterior branches at mid-high of the laminae, whereas in the juvenile specimen MUCPv 595 of *Megaraptor* seem to be separated up to its contact with the diapophysis (Fig 17C, Figure 8D in [35]).

The only dorsal centrum preserved in *Murusraptor* bears a deep lateral pleurocoel with not a well marked dorsal border, unlike the specimen MUCPv 595 that has lateral pleurocoels with well defined enclosing borders (Figure 8C-D in [35]).

### Comparison with *Aerosteon*

The holotype material of *Aerosteon* (MCNA-PV-3137) from the Anacleto Formation of Mendoza [26] includes a single associated tooth crown with three denticles per millimeter on both anterior and posterior carinae. The tooth is relatively small considering how large the animal was, but nevertheless is about ten percent larger than the largest known tooth of *Murusraptor*. Anterior serrations extend along most of the height of the crown of the supposed *Aerosteon* tooth, which is considerably different than the condition in *Murusraptor* in which the anterior denticles are restricted to the tip of the tooth. Because the tooth closely resembles the teeth of abelisaurids [37,78], there is a strong possibility that the tooth identified as belonging to *Aerosteon* is incorrectly associated with this animal; it may in fact have been from an abelisaurid that was scavenging the *Aerosteon* carcass.

The prefrontal of *Aerosteon* is anteroposteriorly long but low [26] as in *Murusraptor*. The postorbitals of the two animals are virtually identical in morphology and size, although the specimen from Mendoza has a weakly developed orbital rugosity [26] that is not present in *Murusraptor*. The facet identified on the medial surface of MCNA-PV-3137 as the suture for the laterosphenoid [26] was misidentified; it does not have a sutural surface, it is too large, extends too far ventrally, and should be continuous with the posteroventral surface of the frontal suture as in all other theropods. The quadrates of *Aerosteon* and *Murusraptor* are the same in major features and size, but have differences in details. The most diagnostic features that they share are the presence of a large quadratic foramen that is entirely enclosed within the quadrate, and is positioned dorsal to the large pneumatic foramen.

The holotype of *Aerosteon* includes much of the vertebral column, including cervicals (Ce1, Ce4, Ce6, Ce8), dorsals (D1, D4-D11, D14), sacrals (S2-S5) and one caudal (Ca1). Presacrals 17 and 20, many of the sacrals and the first caudal are known for both MCNA-PV-3137 (*Aerosteon*) and MCF-PVPH-411 (*Murusraptor*), and overlapping dimensions suggest that the former is no more than 10% larger than the latter. The lengths, widths and heights of centra and neural arches are closely comparable for vertebrae from similar parts of the column. All diagnostic characters, including the presence and absence of pneumatic ones, are the same in the two specimens.

**Fig 28:**
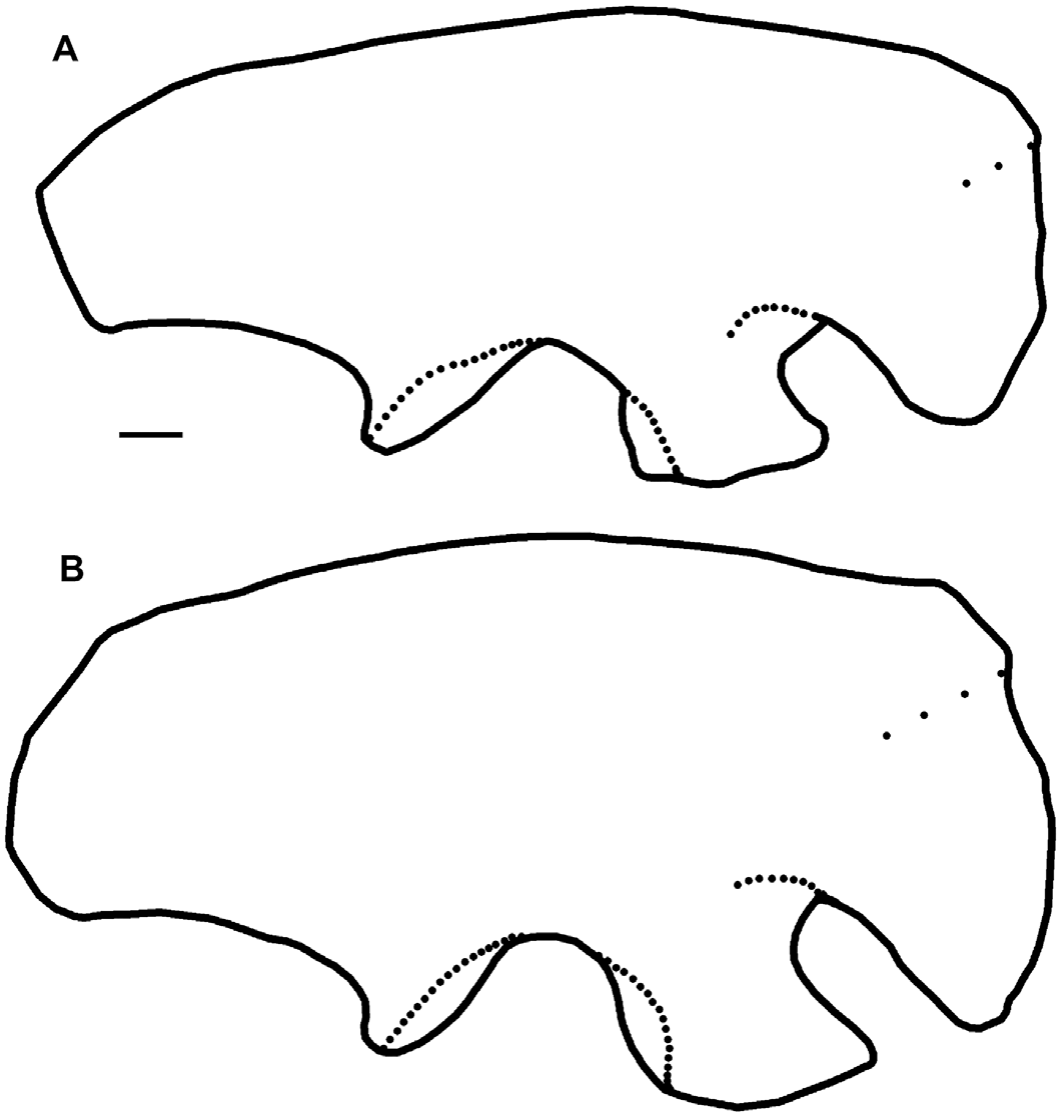
Comparison of right ilia of *Murusraptor* (A) and *Aerosteon* (B) in lateral views. Scale bar: 10 cm.

The ilia of MCNA-PV-3137 (*Aerosteon*) and MCF-PVPH-411 (*Murusraptor*) are anatomically indistinguishable, although there are differences in proportions (Fig 28).

For example, the ilium of MCNA-PV-3137 is only 2.4% longer than that of *Murusraptor* (MCF-PVPH-411), but is 17.5% taller above the acetabulum (Fig 28 and Table 2). The antiliac blade is about the same length in the known specimens of *Aerosteon* and *Murusraptor*, but the postiliac blade is slightly longer in the former. There are also slight differences in the positions of pneumatopores on the medial surface of the ilium. The proximal ends of the pubes are present in both specimens, and show that no significant anatomical differences between the orientations of these bones in the two taxa, nor in the proportions of the iliac and ischial peduncles.

**Table 2:** Comparison of the iliac and ischial sutural surfaces of the pubis, showing that *Murusraptor* (MCF-PVPH-411) is larger than *Megaraptor* (MUCPv 341), but more gracile.

		Ant-post L	Lat-med W	Dors-vent H
MCF-PVPH-411	Iliac suture	176	70	--
MCF-PVPH-411	Ischial suture	--	40	90
MCNA-PV-3137	Iliac suture	169	81	
MUCPv 341	Iliac suture	139	86	
MUCPv 341	Ischial suture	--	57	72

Measurements for *Aerosteon riocoloradensis* (MCNA-PV-3137) taken from [16] Abbreviations: Ant-post L, anterior-posterior length; Dors-vent H; dorsoventral height; Lat-med W, lateromedial width. All measurements are in millimeters.

### Comparison with *Orkoraptor*

*Orkoraptor burkei* [28] is another megaraptoran with clear affinities to *Murusraptor* (MCF-PVPH-411). Collected at Cerro Los Hornos from Cenomanian-Santonian rocks in the province of Santa Cruz in southern Patagonia, the specimen includes cranial (nasal, postorbital, quadratojugal, teeth) and postcranial (vertebrae, chevrons, ribs, tibia) material, and was clearly an unusual theropod. The incomplete tibia was estimated to have been about 60 cm in length [28], which is only slightly shorter than the tibia of MCF-PVPH-411. The teeth and postorbital are similar to those of *Murusraptor* (MCF-PVPH-411). Like *Megaraptor* [8] and probably MCF-PVPH-411, the caudals have pneumatopores. The differences in preservation and the incompleteness of this specimen make it difficult to determine its precise relationships with *Murusraptor*. Lack of an anterior carina was scored for lateral teeth of *Orkoraptor* [28]. However, in *Murusraptor*, the anterior carina is restricted to the distal tip of each lateral tooth. Because this region cannot be seen in any specimen of *Orkoraptor*, this character has been rescored from “1” to “?”.

## Phylogenetic Analysis

The phylogenetic relationships of Megaraptoridae are currently the focus of intense debate. This interesting clade of South American theropods is, at present, considered alternatively as member of the Allosauroidea [25,38] or as the sister group of the Tyrannosauroidea [35,37].

Thus, in order to establish the phylogenetic relationships of *Murusraptor*, two alternative data matrices were considered. First, we tested the data set proposed by Carrano et al. [38], plus modifications proposed by Zanno and Makovicky [39] and three new characters (Analysis A, see S1 and S2 Files). Second, we tested the data set proposed by Novas et al. [37] plus the modifications from Porfiri et al. [35] (Analysis B, see S1 and S2 Files). In order to facilitate the reading of each analysis, the character numbers from Analysis A are in regular type, wheras the character numbers from Analysis B are shown in **bold**. Both analyses were processed by using the TNT software [42] with the application of Traditional Search analysis.

**Fig 29:**
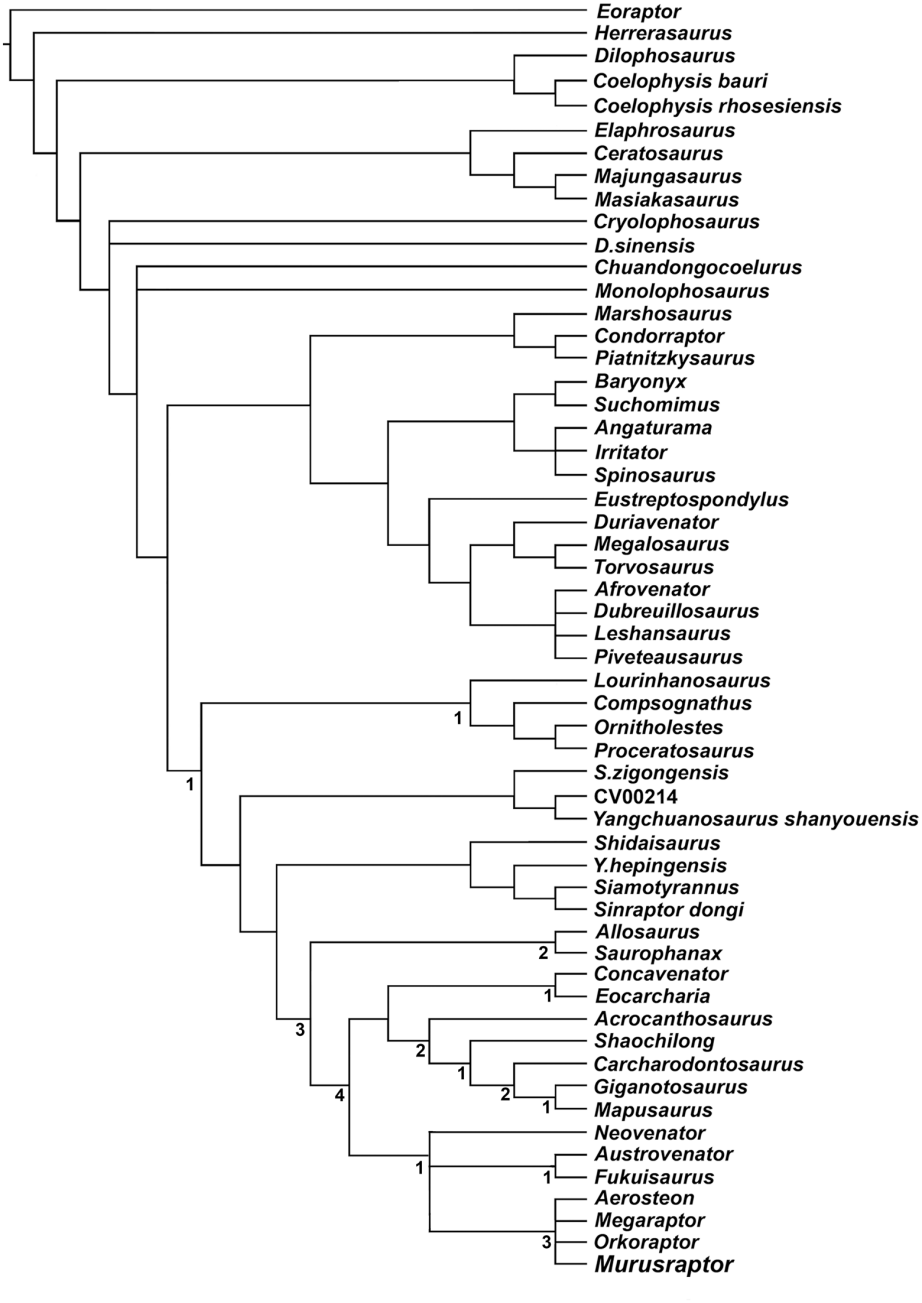
Cladogram depicting the phylogenetic position of *Murusraptor* according Analysis A. Numbers in nodes indicate Bremer support.

The results obtained from Analysis A consists in 358 cranial and postcranial characters distributed among 64 theropod taxa. The Traditional Search option provided 4650 most parsimonious trees (MPTs) of 1097 steps with a Consistency Index of 0.392 and Retention Index of 0.680. The strict consensus showed a monophyletic Neovenatoridae [25] that includes an unsolved politomy among *Neovenator*, *Siats meekorum*, *Chilantaisaurus* and the *Megaraptora* [25] (Fig 29).

*Murusraptor* is nested within the Megaraptora (sensu [25]) as a member of a group formed exclusively by the South American taxa (that includes *Aerosteon*, *Megaraptor*, and *Orkoraptor*) that here are identified as members of the Megaraptoridae [37], which also includes *Australovenator* and *Fukuiraptor*. The Pruned Trees option shows the mostly incomplete taxa *Metriacanthosaurus*, *Tyrannotitan*, *Streptospondylus*, *Poekilopleuron*, *Magnosaurus*, *Chilantaisaurus* and *Siats meektorum* as “wildcards”. When these seven taxa were inactivated, the new analysis resulted in only 7 MPTs of 1073 steps, with CI = 0.400 and RI = 0.691. The reduced consensus recovered *Neovenator* as the sister taxon of Megaraptora, with no changes in the topology previously obtained for *Murusraptor* and other megaraptorids.

*Murusraptor* shares with all Megaraptoridae two unambiguous synapomorphies: teeth with no enamel wrinkles (interpreted as a reversion to primitive condition in Theropoda) (character 143); and anterior caudal vertebrae with neural arch bearing prominent centrodiapophysial laminae that define a deep infradiapophysial fossa (character 205). On the other hand, due incompleteness of available evidence of some of the members of the clade, *Murusraptor* shares other features with some members of the Megaraptoridae: dorsal vertebrae with distinct step-like ridge lateral to hyposphene (character 189) and medial gastralia fused on midline (character 356) (unknown in *Orkoraptor*); maxillary and dentary teeth with anterior carina restricted to the dental tip (10 or fewer denticles) (character 147, unknown in *Aerosteon*). *Murusraptor* and *Megaraptor* share the presence of a basioccipital with a sharp dorsoventrally oriented laminae situated immediately ventral to the occipital condyle, proximal caudals neural spines with distal squared thickeness, and lateral borders of frontals parallel to midline (character 91, 357, 358; unknown in *Orkoraptor* and *Aerosteon*), and dorsal vertebrae with pneumaticity/webbing at the base of the neural spines (character 181, unknown in *Orkoraptor* and primitive stage in *Aerosteon*). *Murusraptor* shares with *Orkoraptor* a postorbital with a ventral process with U-shaped cross-section (character 57, unknown in *Megaraptor* and primitive stage in *Aerosteon*) and with *Aerosteon* an ilium highy pneumatized brevis fossa and sacral attachments (character 262, unknown in *Orkoraptor* and *Megaraptor*).

*Murusraptor* also shares with *Neovenator* and megaraptorids (unknown in *Orkoraptor*) the presence of middle and posterior dorsal vertebrae with postzygapophyses bearing tab-like lateral extensions of articular facets (character 190), ilium with string developed ridge on medial surface adjacent to preacetabular notch (character 273, unknown in *Megaraptor* and *Orkoraptor*) and tibia with anterolateral condyle prominent and ventrally curved (character 321, unknown in *Megaraptor* and *Aerosteon*). The presence of transversely narrow manual ungueals is shared by *Murusraptor*, *Megaraptor*, *Fukuiraptor* and *Australovenator* (character 260, unknown in *Neovenator*, *Orkoraptor* and *Aeosteon*), whereas *Murusraptor*, *Australovenator* and *Aerosteon* share a tibia with anteromedial buttress for astragalus bearing bluntly rounded vertical ridge on medial side (character 322, unknown in *Fukuiraptor*, *Orkoraptor* and *Megaraptor*).

**Fig 30:**
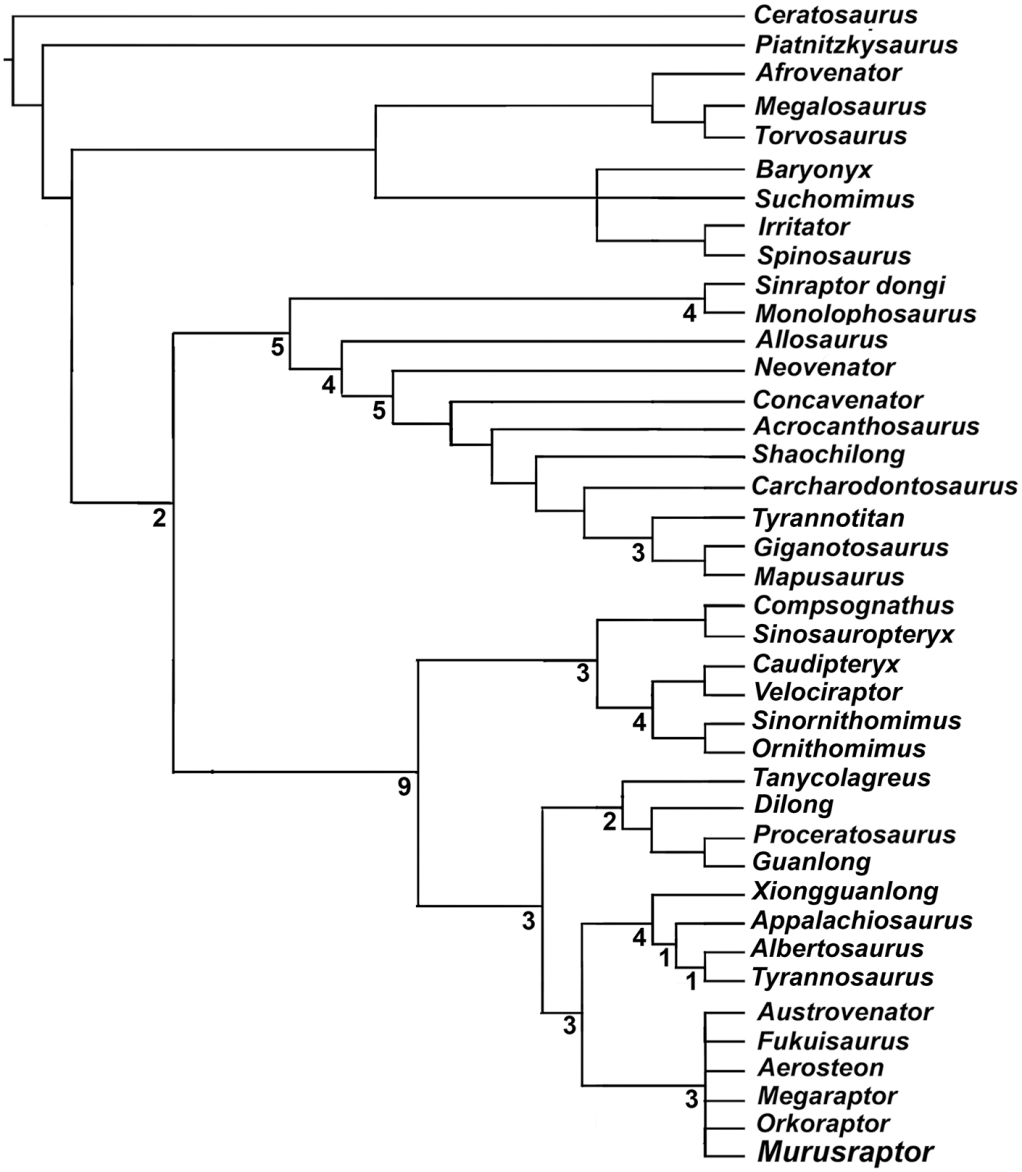
Cladogram depicting the phylogenetic position of *Murusraptor* according Analysis B. Numbers in nodes indicate Bremer support.

In the results from Analysis B were obtained from a matrix of 284 characters distributed among 46 theropod taxa, which included the character scoring for *Murusraptor* (see S1 and S2 Files). The analysis results in a best score of 934 steps and 214 MPTs., with a CI = 0.370 and a RI = 0.653. The analysis is rooted in *Ceratosaurus* as the outgroup and the consensus tree shows a unsolved politomy at the level of all Neotetanurae. Some taxa are indicated as conflictive according the Pruning option of the software. Accordingly, *Carcharondontosaurus iguidensis*, *Eotyrannus*, *Chilantaisaurus*, *Kileskus*, *Eocarcharia* and *Santanaraptor* were inactivated for a second running. Unlike Porfiri et al. [35], *Orkoraptor* was kept among the active taxa because its importance within the megaraptors and its comparison with *Murusraptor*. In this second analysis, 4 MPTs of 902 steps were obtained, with a CI = 0.384 and RI: 0.672. *Murusraptor* is recovered as a megaraptorid in the Analysis B (Fig 30) and a monophyletic clade formed by all South American taxa plus *Fukuiraptor* and *Australovenator* is also recovered as a unsolved politomy.

On the basis of the analysis carried on Analysis B, *Murusraptor* shares with all known megaraptorids the presence of proximal caudal vertebrae with centrodiapophyseal laminae comparable in prominence or more developed than those of the dorsal vertebrae (character **115**, also present in some spinosaurids). Shared characters with some megaraptorids but unknown in others involve teeth without mesial denticles (character **2**, unknown in *Aerosteon* but also present in *Sinosauropteryx* and *Compsognathus*), dorsal ribs proximally pierced by foramina (character **116**, also present in some allosaurids) and more than a pair of gastralia proximally fused at midline with expanded club-shaped proximal ends (character **117**, also present in tyrannosauroids and neovenatorids) (both characters unknown in *Orkoraptor* but also present in *Australovenator*); pubis with pubic tubercle present as a convexity on the anterior margin of the pubis (character **158**, unknown in *Orkoraptor* but also present in *Velociraptor* and *Guanlong*). Finally, *Murusraptor* and *Aerosteon* share the lateral surface of ilium with large external foramina and internal (character **245**, unknown in *Orkoraptor* and *Megaraptor*).

According this analysis, the nesting of *Murusraptor* within Tyrannosauroidea is supported by sixteen sinapomorphies: maxillary and dentary teeth crowns with mesial and distal margins strongly curved, with the apex positioned well distally from the distal margin (character **8**); reduced or absent prefrontal (character **39**); quadrate with a deep recess on the anterior surface where the pterygoid wing and condyles meet (character **67**, also in *Giganotosaurus* and *Mapusaurus*); supraacetabular crest of the ilium with a maximum lateral projection subequal relative to ischial peduncle (character **151**); tibia with the medial malleolus oriented almost medially, 'shoulder' absent (character **187**, also present in *Neovenator*); distal medial malleolus of the tibia expanded 40% or more than tibial mid-shaft width (character **190**, also present in allosauroids); lateral malleolus of the tibia extended distally beyond the medial malleolous more than 5% of tibial total length with respect to medial malleolus (character **191**, also present in *Neovenator* and carcharodontosaurids); tibia with lateral condyle of proximal end curving ventrally as a pointed process (character **246**, also present in *Neovenator*); ratio of anteroposterior length a single frontal exposed on skull roof to mediolateral width at midpoint less than 2.0 (character **261**, also present in *Allosaurus* and *Shaochilong*); ventral ramus of the postorbital substantially anteroposteriorly wider than ventral ramus of the lacrimal at midpoint, parietal with the skull table between supratemporal fosase extremely reduced, sagittal crest or crests pinched between opposing fosae and basisphenoid recess of the basipterygoid oriented posteroventrally, recess partially or widely visible in posterior view (characters **263**, **266** and **269**, also present in carcharodontosaurids); and basisphenoid with pronounced muscle scars flanking basisphenoid recess.

On the other hand, *Murusraptor* exhibits the plesiomorphic stage among Tyrannosauroidea by having premaxillary teeth subequal in size to rostral maxillary teeth, premaxillary teeth with recurved posterior crowns, and premaxillary teeth without median vertical ridge on lingual surface (characters **3**, **5**, **6**, also present in *Megaraptor* and *Fukuiraptor*); an orbit with a rounded contour, lacrimal-postorbital contact absent, and postorbital with dorsally oriented anterior process (characters **16**, **44**, **51**, also present in *Orkoraptor* and *Aerosteon*); size of the external mandibular fenestra more than 10% total mandibular length, dorsoventral depth of the surangular less than half the maximum width of the mandible above the mandibular fenestra, tibia with a maximum length equal to or less than 12 times the anteroposterior width at mid-length, a roughly symmetric calcaneum with wide angles in the posterior border, a lateral condyle of the at the posterior rear with its posterior margin located at the same than the posterior margin of the medial condyle, absence of a vertical ridge on iliac blade above acetabulum (characters **85**, **87**, **186**, **201**, **221**, **242**, also present in *Aerosteon*); articular with the mediolateral width of jaw muscle attachment site less than the width of glenoid for articulation with quadrate, and a smooth non-articular region between glenoid and attachment site for depressor mandibular muscles (characters **275** and **276**).

Interestingly, according the results obtained by running the data set from Analysis B and the position of Megaraptora among tyrannosauroids, *Murusraptor* exhibits seventeen characters that are interpreted as convergencies of this taxon with non-tyrannosauroid theropods: spiral suture postorbital-squamosal articulation with a wide medial process (character **45**, also present in carcharodontosaurids); lacrimal with a small pneumatic recess (character **54**, also present in allosauroids); lacrimal with no lacrimal horn (character **55**, also in some basal tetanurans); quadrate foramen present, surrounded by the quadrate (character **66**, also present in *Aerosteon* and some basal allosauroids); a highly pneumatic braincase (character **69**, also present in *Megaraptor* and carcharodontosaurids); basal tubera formed by basioccipital and basisphenoid, not subdivided (basioccipital located posteriorly and basisphenoid located anteriorly) (character **70**, also present in basal allosauroids); paroccipital processes strongly tilted ventrally, with its distal end entirely located under the ventral level of the foramen magnum (character **72**, also present in Allosauroidea); laterosphenoid with the opening for V cranial nerve located posterior to the nuchal crest (character **73**, also present in carcharodontosaurids); presence of a deep and funnel-shaped fossa in the basisphenoid (character **78**, also present in *Megaraptor* and carcharodontosaurids); dorsal expansion width of the supraoccipital at least twice the width of foramen magnum (character **81**, also present in *Xiongguanlong* and carcharodontosaurids); ectopterygoid with a pneumatic recess as a deep and subcircular depression (character **84**, also present in allosauroids); posterior dorsal vertebrae centra shorter than its depth (character **107**, also present in *Albertosaurus*, *Neovenator* and *Acrocanthosaurus*); anteriorly oriented neural spines of posterior dorsals (character **108**, also present in aerosteon and some allosauroids); sacral vertebrae with pleurocoels (character **109**, also present in *Megaraptor*, some carcharodontosaurids and *Tyrannosaurus*); caudal vertebrae with, hyposphene-hypantrum accesory articulationswell-developed and extended approximately along the first third of the tail (character **111**, also present in *Tyrannosaurus* and basal allosauroids); ischium with a distal expansión (character **169**, also present in *Giganotosaurus* and *Mapusaurus*); and a lacrimal with a suborbital process (character **230**, also present in some allosauroids).

The phylogenetic relationships of *Murusraptor barrosaensis* are, beyond any reasonable doubt, sufficiently supported as a Megaraptoridae and, even more importantly, probably a member of a more inclusive taxon representing a South American diversification of megaraptorids together with *Megaraptor*, *Aerosteon* and *Orkoraptor*.

The polarization between the hypotheses involving the phylogenetic relationships of the Megaraptoridae, is probably due the utilization of certain different selection criteria in the comparative taxomomic universe (including the outgroup taxa chosen to check the plesiopmorphic stage of the characters) and the relative consideration of the phylogenetic relevance of the chosen morphological features.

Eventually, further discoveries of more elecuent specimens in older rocks from both South America and Australia will help to a better understanding of the early evolution of the clade and, therefore, clarify its basal phylogenetic affinities.

## Supporting Information

S1 File Data Matrices A and B. (TXT)

S2 File New characters scores and new characters. (DOC)

S3 File Body length estimations. (DOC)

S4 File Tables with comparative sacrum and tibia lengths. (XLS)
