# Supplementary material for: A New Megaraptoran Dinosaur (Dinosauria, Theropoda, Megaraptoridae) from the Late Cretaceous of Patagonia
Source: PLoS One. 2016 Jul 20;11(7):e0157973. doi: 10.1371/journal.pone.0157973 (PMC4954680; doi:10.1371/journal.pone.0157973)
Supplement: S2 File — (DOC) [file pone.0157973.s002.doc]

Supplementary information 2

Revised scores for Carrano et al. (2012)

Character 61 coding was modified for *Majungasaurus* (recoded to “1”)

Character 81 has been modified to code the quadrate foramen as; 0, between the quadrate and quadratojugal; 1, reduced or absent; 2, enclosed entirely by the quadrate (modified from Carrano et al. 2002; Rauhut 2003; Tykoski 2005; Smith et al. 2007). Even though the quadrate is not preserved in *Orkoraptor*, the quadratojugal shows that it had a complete sutural contact with the quadrate and did not take part in the margin of the quadrate foramen.

Character 143. The character has therefore been recoded as “0” for *Aerosteon*. *Aerosteon* teeth are coded in Benson et al. (2009) as having wrinkles in the enamel, but these are not evident in tooth figured by Sereno et al. (2008, fig. 2).

Character 147 has been modified to code the extent of the anterior carinae in maxillary and dentary teeth as: to the base of crown (0), at least a third of the way down the crown (1), restricted to tip (10 or fewer denticles) (2).

Character 191 has been recoded as “1” in *Aerosteon* and *Neovenator*.

Character 192 has been recoded as “0” in *Aerosteon* and *Neovenator*.

Characters 201 deals with the degree of pneumaticity in dorsal and sacral neural arches. This character, which is present in most theropods, was inactivated because it is subjective and difficult to quantify. Furthermore, abelisaurids, which were coded as a family, show multiple states.

Character 262 was modified to recognize that *Aerosteon* and *Murusraptor* are unique in that the iliac blade is pneumatized through sacral attachments on the medial surface. The character now reads “Ilium, pneumatization; 0, not present; 1, pneumatized through brevis fossa; 2, pneumatized through brevis fossa and sacrum.”

Character 264 coding was modified for *Fukuiraptor* (recoded to “1”).

Character 270 coding was modified for *Fukuiraptor* (recoded to “?”).

Character 282 coding was modified for *Australovenator* (recoded to “0”), *Mapusaurus* (recoded to “1”).

Character 296 has been recorded as “0” in *Neovenator* (the character also needs to be rewritten as it reads “between…or”.

New characters

Character 356 (new). Gastralia, medial gastralia; 0, most unfused; 1, fused on midline.

Character 357 (new). Proximal caudals neural spines: lateral sides flat, 0; distal squared thickeness, 1.

Character 358 (new). Lateral borders of frontals: diverging from midline, 0; parallel to midline, 1.

Character scores for *Murusraptor*, *Orkoraptor* and *Megaraptor* in Zano and Makovicky (2013) plus three new characters (this contribution).

*Murusraptor* 0?????????????????????????????????????????210100000??????11?201000110110?????1?1002111100101010210101001200?0?????00111????????????10111?1???000000210???????1012????????????????????101??0011100011210001?1?1????1?1000000?????????????????????????????????????????1?201111011022111011020????????0?00??000?????????????????10011402??????11???????????????????1??1111

*Orkoraptor* 0????????????????????????????????????????????????????????11?2010????????????0????????????????????????????????????????????????????????????????010000210????????????????????????????????????????????????????11110????????????????????????????????????????????????????????????????????????????????????????????????????????????????011?02???????????????????????????????

*Megaraptor*

0??????????????????????????????????????????????????????????????????????????????????????????????????????????????????????????????????????????????????2????????11012????????11?0100121?0????????1??????????????110?0?1?????1?111011?0121????????????0101011?2?01011111110??????????????????????2????????????????????????????????????????????????????????2?11???????????111

Character scores for *Murusraptor* in Porfiri et al. (2014)

020?0001???????0??????????????????????10000020?0?000021??????????2112101100001011??2010????????????????1??101010??111????????????????????????????102??11012??1?????0???01????????????????020111????????00??????2?????2?1??0?0??0?0???100?????0???0?111????????10???0101??11111???200?????0?0

Bibliography

Carrano, M. T., Benson, R. B. J., and Sampson, S. D. 2012. The phylogeny of Tetanurae (Dinosauria: Theropoda). Journal of Systematic Palaeontology 10: 211–300.

Porfiri, J.D., Novas, F.E., Calvo, J.O., Agnolín, F.L., Ezcurra, M.D. and Cerda, I.A. 2014. Juvenile specimen of *Megaraptor* (Dinosauria, Theropoda) sheds light about tyrannosauroid radiation. Cretaceous Research 51:35-55.

Rauhut, O.W.M. 2003. Special Papers in Palaeontology, The Interrelationships and Evolution of Basal Theropod Dinosaurs (No. 69). Blackwell Publishing.

Sereno, P. C., R.N. Martinez, J. A. Wilson, D. J. Varrichio, O.A. Alcober, and H.C.E. Larsson. 2008. Evidence for Avian intrathoracic air sacs in a new predatory dinosaur from Argentina. PLoS One 3(9): e3303. doi:10.1031/journal.pone.0003303

Smith, N. D., Makovicky, P. J., Hammer, W. R., & Currie, P. J. 2007. Osteology of Cryolophosaurus ellioti (Dinosauria: Theropoda) from the Early Jurassic of Antarctica and implications for early theropod evolution. Zoological Journal of the Linnean Society, 151(2), 377-421.

Tykoski, R. 2005. Osteology, ontogeny, and relationships of the coelophysoid theropods. Unpublished PhD thesis, University of Texas at Austin.

Zanno, L.E., and Makovicky, P.J. 2013. Neovenatorid theropods are apex predators in the Late Cretaceous of North America. Nature Communications 4: 2827, doi:10.1038/ncomms3827.
