# Supplementary material for: A New Megaraptoran Dinosaur (Dinosauria, Theropoda, Megaraptoridae) from the Late Cretaceous of Patagonia
Source: PLoS One. 2016 Jul 20;11(7):e0157973. doi: 10.1371/journal.pone.0157973 (PMC4954680; doi:10.1371/journal.pone.0157973)
Supplement: S3 File — (DOC) [file pone.0157973.s003.doc]

**Body size**

*Murusraptor* is a member of the recently defined Megaraptora (Benson et al. 2010), which is the most inclusive clade comprising *Aerosteon riocoloradensis*, *Megaraptor namunhuaiquii* and *Orkoraptor burkei*, all of which come from Argentina. *Chilantaisaurus tashuikouensis*, *Neovenator salerii* and Carcharodontosauridae are progressively more plesiomorphic sister taxa. The holotype of *Megaraptor* comes from 50 km to the west of *Murusraptor*, and a second specimen comes from 20 km north, whereas *Aerosteon* comes from 250 km to the north in Mendoza Province. *Orkoraptor* also appears to be a related form, but comes from 1000 km south from Chubut Province. All of the megaraptorans are from different formations.

Assuming that MCF-PVPH-411 is a theropod with relatively normal theropod body proportions, then its total body length at the time of death can be estimated by comparison with other theropods. Of the skeletal elements recovered for this specimen, the sacrum, ilium and tibia were chosen as relatively consistent elements for estimating body length. The sacrum of MCF-PVPH-411 is not complete, but the length of the one recovered sacral vertebra was multiplied by five to produce an estimated length of 525 mm. A bivariate analysis was done of 59 theropod skeletons in which both sacral length and a good estimate of body length was available (Fig.1). The specimens ranged in size from a 0.75 m *Compsognathus* to a 12 metre *Giganotosaurus* skeleton, and the logarithms produced a regression (y = 0.89x + 1.38) with a correlation coefficient of 0.97. This estimate suggests that MCF-PVPH-411 was an animal with a total body length of 6.4 metres.


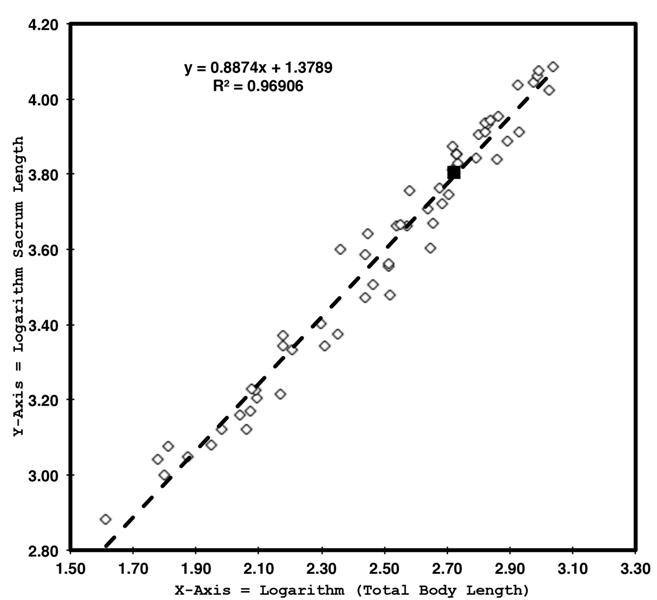


Figure 1. Bivariate analysis for total body length of *Murusraptor barrosaensis*, specimen MCF-PVPH-411. Estimation based on sacrum length. Black square shows *Murusraptor*. Data in Supplementary information file 4.

Similarly, comparing tibia length to estimated body length in 126 theropods produced a regression (y = 1.18x + 0.44, r^2^ = 0.98) that in turn came up with the same estimate of body length for MCF-PVPH-411 (Fig.2).


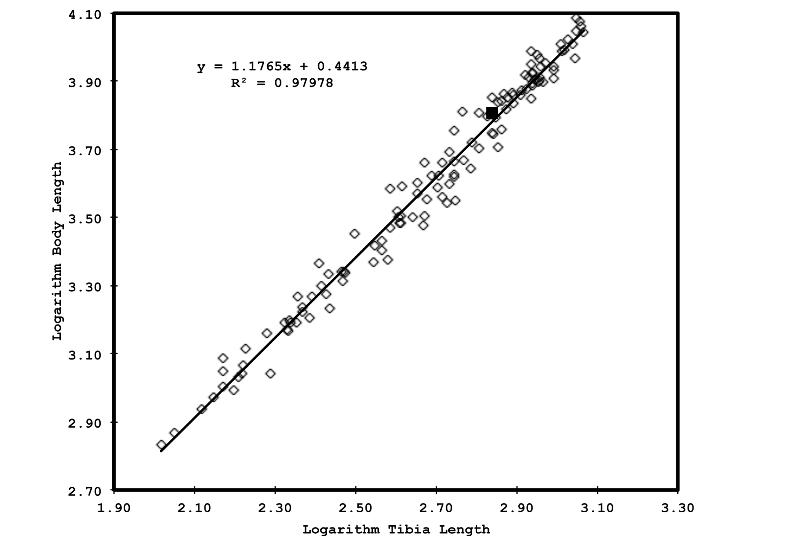


Figure 2. Bivariate analysis for total body length of *Murusraptor barrosaensis*, specimen MCF-PVPH-411. Estimation based on tibia length. Black square shows *Murusraptor*. Data in Supplementary information file 4.

Finally, comparative body length to ilium length available for 95 theropod skeletons produced a regression (y = 0.85x + 1.34, r^2^ = 0.97) that suggests MCF-PVPH-411 was 6.2 meters long. These three measurements, considering how little we know about skull and tail length in this animal, can only give us an approximation of total body length. However, it is reasonable to assume that the animal was somewhere between 6.0 and 6.5 meters in length.

In spite of its relatively large size, this individual was not full grown at the time of death. The bones that form the braincase are tightly connected but the sutures are still visible. Furthermore, the neurocentral sutures were not fused in dorsal and caudal vertebrae, the neurocentral suture was open in the dorsosacral, the neural arch of the dorsosacral was still separate from the neural arch of the first true sacral, and the cervical ribs had not coossified with their vertebrae. Nevertheless, MCF-PVPH-411 must have been approaching maturity because the sphenethmoid had ossified, the braincase bones are tightly knit together, the sacrals are coossified to each other between centra and neural arches, and the distal ends of the pubes were probably fused.

It is interesting to speculate on the possible lifestyles of *Megaraptor* and *Murusraptor*. Although not related to baryonychines and other spinosaurids, these animals had many similarities in body form that suggest similar adaptations for semi-aquatic lifestyles. The elongate, narrow skull is reminiscent of many modern aquatic, piscivorous animals including gavialid crocodilians. These comparisons have already been discussed for spinosaurids and baryonychines, which also include specimens with preserved stomach contents of fish scales (Charig and Milner 1997). Like these animals, *Megaraptor* had large, powerful arms with disproportionately large, highly trenchant claws on their first fingers. Presumably this was used for hooking fish. Almost as noteworthy as the large size of the hands is the relatively short hind limbs of *Megaraptor*, spinosaurids and presumably *Baryonyx*.

Although theropods, especially birds, are characterized by heavily pneumatized skeletons, no large theropods comparable in size with *Murusraptor* are so pneumatic. The degree of pneumatism in the posterior vertebrae (dorsals, sacrals and caudals), most of the anterior ribs and the ilium is consistent with the interpretation that this animal developed a highly specialized metabolism.

Bibliography

Benson, R.B.J., Carrano, M.T., and Brusatte, S.L. 2010. A new clade of archaic large-

bodied predatory dinosaurs (Theropoda: Allosauroidea) that survived to the latest Mesozoic. Naturwissenschaften 97: 71-78, doi:10.1007/s00114-009-0614-x.

Charig, A.J., and Milner, A. C. 1997. *Baryonyx walkeri*, a fish-eating dinosaur from the

Wealden of Surrey. Bulletin of the Natural History Museum, Geology series 53 (1): 11-70.
